# Prediction of Stroke Outcome in Mice Based on Noninvasive MRI and Behavioral Testing

**DOI:** 10.1161/STROKEAHA.123.043897

**Published:** 2023-09-25

**Authors:** Felix Knab, Stefan Paul Koch, Sebastian Major, Tracy D. Farr, Susanne Mueller, Philipp Euskirchen, Moritz Eggers, Melanie T.C. Kuffner, Josefine Walter, Daniel Berchtold, Samuel Knauss, Jens P. Dreier, Andreas Meisel, Matthias Endres, Ulrich Dirnagl, Nikolaus Wenger, Christian J. Hoffmann, Philipp Boehm-Sturm, Christoph Harms

**Affiliations:** Charité Universitätsmedizin Berlin, Freie Universität Berlin and Humboldt-Universität zu Berlin, Klinik und Hochschulambulanz für Neurologie, Department of Experimental Neurology, Germany (F.K., S.P.K., S. Major, T.D.F., S. Mueller, P.E., M. Eggers, M.T.C.K., J.W., D.B., S.K., J.P.D., A.M., M. Endres, U.D., N.W., C.J.H., P.B.-S., C.H.).; Charité Universitätsmedizin Berlin, Freie Universität Berlin and Humboldt-Universität zu Berlin, Center for Stroke Research Berlin, Charité Universitätsmedizin Berlin, Germany (F.K., S.P.K., S. Major, S. Mueller., M. Eggers, M.T.C.K., J.W., D.B., J.P.D., A.M., M. Endres, U.D., N.W., C.J.H., P.B.-S., C.H.).; Charité Universitätsmedizin Berlin, Freie Universität Berlin and Humboldt-Universität zu Berlin, NeuroCure Cluster of Excellence and Charité Core Facility, 7T Experimental MRIs, Germany (S.P.K., T.D.F., S. Mueller, P.B.-S.).; School of Life Sciences, University of Nottingham, United Kingdom (T.D.F.).; Berlin Institute of Health at Charité – Universitätsmedizin Berlin, QUEST Center for Transforming Biomedical Research, Germany (J.W.).; Berlin Institute of Health (BIH), Germany (S.K., N.W., C.J.H., C.H.).; Einstein Center for Neuroscience, Berlin, Germany (J.P.D., M. Endres, U.D., N.W., C.H.).; Bernstein Center for Computational Neuroscience (J.P.D.).; German Center for Cardiovascular Research (DZHK), partner site Berlin (M. Endres, U.D., C.H.).; NeuroCure Clinical Research Center, Charité-Universitätsmedizin Berlin, Berlin, Germany (M. Endres., U.D.).; German Center for Neurodegenerative Diseases (DZNE) (M. Endres, U.D.).

**Keywords:** forecasting, infarction, middle cerebral artery, mice, Inbred C57BL, precision medicine, random forest, sensory motor performance, topographic brain mapping

## Abstract

**BACKGROUND:**

Prediction of poststroke outcome using the degree of subacute deficit or magnetic resonance imaging is well studied in humans. While mice are the most commonly used animals in preclinical stroke research, systematic analysis of outcome predictors is lacking.

**METHODS:**

We intended to incorporate heterogeneity into our retrospective study to broaden the applicability of our findings and prediction tools. We therefore analyzed the effect of 30, 45, and 60 minutes of arterial occlusion on the variance of stroke volumes. Next, we built a heterogeneous cohort of 215 mice using data from 15 studies that included 45 minutes of middle cerebral artery occlusion and various genotypes. Motor function was measured using a modified protocol for the staircase test of skilled reaching. Phases of subacute and residual deficit were defined. Magnetic resonance images of stroke lesions were coregistered on the Allen Mouse Brain Atlas to characterize stroke topology. Different random forest prediction models that either used motor-functional deficit or imaging parameters were generated for the subacute and residual deficits.

**RESULTS:**

Variance of stroke volumes was increased by 45 minutes of arterial occlusion compared with 60 minutes. The inclusion of various genotypes enhanced heterogeneity further. We detected both a subacute and residual motor-functional deficit after stroke in mice and different recovery trajectories could be observed. In mice with small cortical lesions, lesion volume was the best predictor of the subacute deficit. The residual deficit could be predicted most accurately by the degree of the subacute deficit. When using imaging parameters for the prediction of the residual deficit, including information about the lesion topology increased prediction accuracy. A subset of anatomic regions within the ischemic lesion had particular impact on the prediction of long-term outcomes. Prediction accuracy depended on the degree of functional impairment.

**CONCLUSIONS:**

For the first time, we developed and validated a robust tool for the prediction of functional outcomes after experimental stroke in mice using a large and genetically heterogeneous cohort. These results are discussed in light of study design and imaging limitations. In the future, using outcome prediction can improve the design of preclinical studies and guide intervention decisions.

## Teaser

Summary: Systematic analysis of stroke outcome predictors in mice is lacking. We developed a tool to predict late pellet reaching performance based on early stroke localization in a diverse cohort of 215 mice.

Stroke remains a major public health challenge, as its occurrence rises in aging societies.^1^ With decreasing mortality rates due to progress in acute stroke therapy, the need to develop treatments that improve functional outcomes has become more pressing.^2^ Understanding predictors of functional outcome after stroke both in humans and rodents is an essential step towards defining adequate treatment decisions and improving the design of preclinical studies.^3^ Prediction of expected motor-functional outcomes in mice could potentially guide researchers in their treatment decisions in preclinical stroke intervention studies and support outcome-dependent stratifications. In humans, magnetic resonance imaging (MRI) is used to assess stroke volume and topology.^4^ Lesion volume is most commonly used to analyze stroke severity and predict functional outcome, but the prognostic value has been shown to be better when including lesion topology.^5–11^ This was also shown in an exploratory preclinical study using a porcine stroke model.^12^ Additionally, the degree of the subacute deficit has been shown to be a reliable predictor of long-term outcomes, especially for mild functional deficits.^13,14^ In contrast to humans, little is known about predictors of functional outcomes after stroke in mice, although they are commonly used in preclinical research. Detecting motor-functional deficits in rodents following experimental stroke is difficult as they show impressive recovery and have subtle, if any, long-term motor deficits in common behavioral tests.^15^ The stroke preclinical assessment network recently completed the largest preclinical stroke study on mice and provided examples of how the middle cerebral artery occlusion (MCAO) model can be used to conduct studies that are similar to those performed in humans.^16^ Various efforts to standardize preclinical stroke models have been made, including standardization of operation procedures and monitoring of cerebral blood flow.^17,18^ Today, heterogeneity of the MCAO model has been reduced but still largely depends on factors such as the experience of the surgeon or filament choice.^19^ Additionally, other factors such as the natural microbiome, comorbidities, age, or sex are likely contributing to the observable heterogeneity in preclinical stroke studies. In their most recent publication, the stroke preclinical assessment network consortium has suggested to embrace and systematically integrate this heterogeneity of outcomes into preclinical research, to more accurately simulate the reality of clinical studies.^19^

In a previous study, we have investigated the accuracy of early perfusion/diffusion MRI for final infarct prediction.^20^ The main goal of the present retrospective study was to identify and compare different predictors of functional outcome following brain ischemia in mice and to provide researchers with a tool, which can help to cope with the heterogeneity of stroke volumes. Our work opens new avenues toward a better understanding of poststroke recovery and impairment in mice and paves the way to using the prediction of functional outcomes for the optimization of preclinical stroke studies. The provided prediction tool gives laboratories, in particular, those that are just beginning to gradually increase standardization of the MCAO model in an iterative process, the option to cope with heterogeneity of stroke volumes from various sources.

## METHODS

Data are reported according to the ARRIVE guidelines (Animal Research: Reporting of In Vivo Experiments). Data are openly accessible on zenodo (https://doi.org/10.5281/zenodo.6534690). The repository contains all raw MRI T2w images, lesion masks, and the registered atlases, the input for machine learning algorithms (lesion volume, segmented MRI, behavioral scores), the trained classifiers, and their output (predicted behavioral scores).

### Heterogeneity of Stroke Volumes

**Figure 1.**
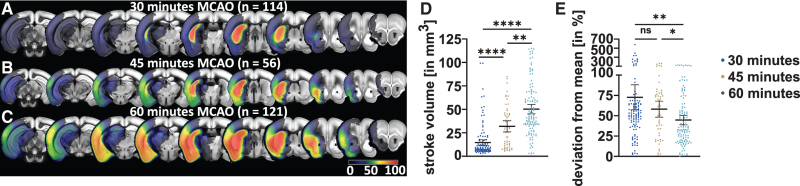
**Heterogeneity of stroke volumes by occlusion time. A**, Lesion incidence maps of mice with 30, **B**, 45, or **C**, 60 min of middle cerebral artery occlusion (MCAO). Notably, an increasing percentage of the mice show affection of cortical regions and, therefore, of the entire MCA territory. **D**, Stroke volumes resulting from the 3 different arterial occlusion times. Lesion volumes became significantly larger with longer occlusion times. Error bars indicate 95% CI; black line indicates mean. **E**, Individual deviation from group mean was expressed in percent. Interestingly, the 60 min of MCAO provided significantly smaller deviations, indicating more homogeneity of stroke volumes at longer occlusion times.

We compared the degree of stroke volume heterogeneity caused by different arterial occlusion times using imaging data from a total of 291 C57Bl/6 mice undergoing either 30 (n=114), 45 (n=56), or 60 (n=121) minutes of MCAO. Mean lesion volumes and deviation from group mean in percent were assessed (Figure 1). We additionally performed a covariate analysis using a multiple linear regression model assessing the effect of surgeon and occlusion time on stroke lesion volume. Subsequently, imaging and behavioral data from 15 studies performed between 2015 and 2019 applying an occlusion time of 45 minutes and 15 genotypes were collected (Figure S1). For this cohort, which was later divided into a prediction cohort and a replication cohort, we analyzed the effect of the covariates genotype and surgeon (n=2) on the heterogeneity of stroke lesion volumes.

### Experimental Design and Selection Process

**Figure 2.**
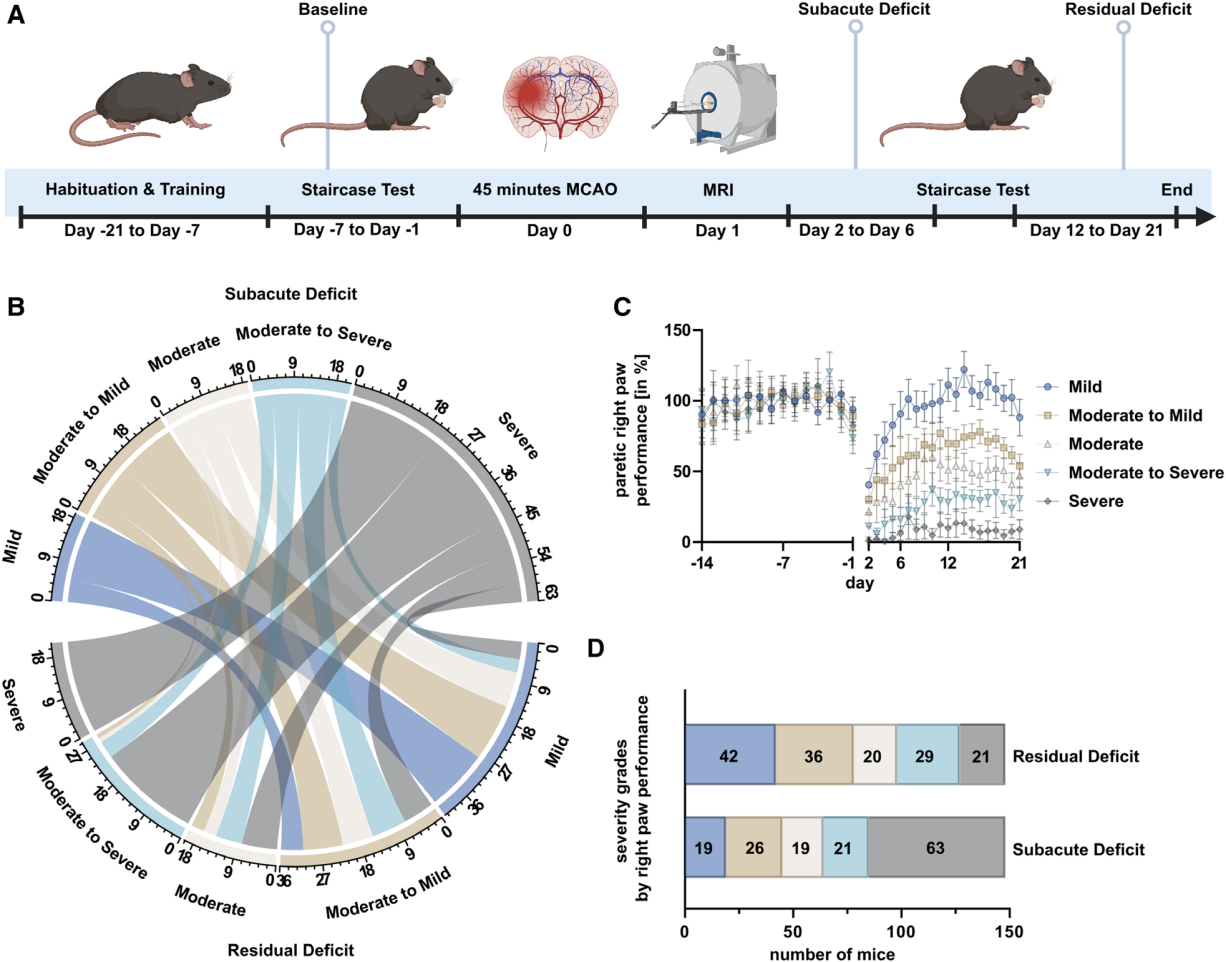
**Experimental design and assessment of functional subgroups. A**, Mice were trained in pellet reaching for 21 d. Average percentage of retrieved pellets during the last week before middle cerebral artery occlusion (MCAO) was used to normalize performance (baseline). Mice received either 45 min of MCAO or sham surgery. Magnetic resonance imaging was performed on day 1 and mice were then tested for another 21 d. **B**, Chord diagram displaying the different trajectories of recovery. Groups on top display mice stratified by degree of subacute deficit, while groups on bottom display mice stratified by residual deficit. Numbers indicate group sizes. Interconnections visualize how many mice with a given subacute deficit recovered to have a given residual deficit, that is, roughly a third of the mice with a severe subacute deficit displayed no recovery and showed a severe residual deficit. **C**, Performance of the paretic right paw over time for each severity grade. Error bars indicate 95% CI. Severity grades here were based on the degree of the residual deficit. **D**, Overview over sizes of functional subgroups. Groups are either based on the degree of subacute or residual deficit. While the majority of mice displayed a severe subacute deficit, due to their impressive capability for spontaneous recovery, the majority of mice displayed only mild residual deficits. Created with BioRender.com.

The prediction tools were developed using data from the prediction cohort and were tested again in the replication cohort. Skilled forepaw reaching was tested in the staircase test before and after MCAO surgery, followed by MRI after 24 hours (Figure 2A, details in Supplemental Material and Methods). Initially, 450 mice were considered for inclusion and 215 mice were included in this retrospective study (consort diagram, Figure S1B; Table S1). Basic characteristics of all animals are provided in Table S2 and Figure S2. A detailed report on the selection criteria and mortality can be found in the Supplemental Material and Methods.

### Staircase Test of Skilled Reaching

Behavioral testers were blinded to the intervention and MRI results. Mice were housed, food-restricted, and familiarized with the staircase test (details in Supplemental Material and Methods).^21^ The percentage of collected pellets per side was chosen as the experimental variable. Performance was expressed as a percentage of average prestroke performance (baseline). Based on the mean performance of the entire cohort after the stroke, 2 phases were defined: a subacute deficit phase (days, 2–6), during which performance appeared dynamic and increasing, and a residual deficit phase (days, 12–21), throughout which performance appeared stable. Average performance was calculated for each mouse for the respective period. A gap of 5 days was chosen between the subacute and residual deficit to ensure the dynamic phase had ended.

### Classification of Severity Grades for Subgroup Analysis

Alluding to the modified Rankin Scale, a score regularly used in patients with stroke, 5 different functional subgroups were defined based on the severity of deficit (Figure 2B through 2D; details in Supplemental Material and Methods).^22^

### Selection of Predictors

Two factors were analyzed for their predictive value for the degree of the subacute deficit after MCAO: (1) stroke lesion volume measured within the coordinates of the Allen Mouse Brain Atlas, which makes it identical to the volume of remaining brain tissue^23^ and (2) MRI lesion percentages measured in the 736 Allen Mouse Brain Atlas regions. Throughout the article, this predictor is referred to as segmented MRI. The dimensionality of the segmented MRI (736 initial regions) was reduced by discarding all regions that did not show a lesion in at least 1 animal. This left the segmented MRI with 536 regions. In addition to those 2 imaging-based parameters, for the prediction of the residual deficit, the degree of subacute deficit was included as a third factor.

### Development of Prediction Models With Machine Learning

For the development of machine learning–based prediction models, we divided the prediction cohort into training (two-thirds of the animals) and testing (one-third of the animals). For each predictor, we calculated the median absolute error and reported the average as prediction error (PE). Only PEs from the test cohort were used for accuracy analysis. PE measures the difference between predicted and actual performance. Small errors indicate high accuracy. To determine which anatomic regions affect MRI-based residual deficit prediction accuracy, the out-of-bag predictor importance for each anatomic region in the segmented MRI was calculated (details in Supplemental Material and Methods).^24^ The prediction models were uploaded and are available at github.com/major-s/mouse-mcao-outcome-predictor.

### Statistics

Methods for statistics are described in the Supplemental Material and Methods.

### Ethics Approval

All animal procedures were approved by and performed in concordance with local authorities (Landesamt für Gesundheit und Soziales) under license numbers G0197/12, G005/16, G0057/16, G0119/16, GG254/16, G0157/17, and G0343/17.

## RESULTS

### Occlusion Time, Surgeon, and Genotype Influence Heterogeneity of Stroke Volumes

Figure 1A through 1C show stroke incidence maps and edema-corrected lesion volumes for the 3 occlusion times. After 30 minutes, the mean lesion volume was 14.6 mm^3^ (95% CI, 11.6–17.6), 31.9 mm^3^ (26.0–37.9) after 45 minutes, and 50.2 mm^3^ (45.2–55.2) after 60 minutes (Figure 1D). Volumes differed across occlusion times (χ^2^=115.0; *P*<0.0001). The 30-minute MCAO group had a 72.7% (57.4–88.0) deviation from the group mean, the 45-minute group 58.1% (48.3–68.0), and the 60-minute group 44.7% (38.9–50.6; Figure 1E). Deviation from group mean differed significantly among the 3 groups (χ^2^=14.7; *P*=0.0006). The difference in deviation from group mean between the 30- and 60-minute MCAO groups (Z=3.5; *P*=0.002) and the 45- and 60-minute groups (Z=2.85; *P*=0.013) was statistically significant. Linear regression model demonstrated that the occlusion time (*F*=143.7; *P*<0.0001) but not the surgeon (*F*=0.8; *P*=0.5) affected stroke lesion volumes in 291 C57Bl/6 mice (Figure S3). We further examined the effects of genotype and surgeon on stroke lesion volumes in the prediction cohort. Linear regression model indicated that genotype (*F*=2.2; *P*=0.008) and surgeon (*F*=9.0; *P*=0.003) affected stroke volume.

### MRI Characterization and Lesion Topology in Prediction and Replication Cohort

T2-weighted MR images were coregistered on the Allen Mouse Brain Atlas to evaluate lesion topology (segmented MRI) and incidence maps were generated (Figure S4A and S4B). Quantitative evaluation of the hyperintense lesion was performed on 536 regions and lesion volume in each region was expressed in percent of the total region (Table S3). Lesion volumes and topological characteristics varied slightly between the prediction and replication cohort (Figure S4C and S4D). Further, incidence maps and lesion volumes were calculated for the functional subgroups (Figure S5) and more details can be found in the Supplemental Material and Methods.

### A Modified Staircase Test Can Assess Early and Long-Term Functional Outcome in Mice After Stroke

**Figure 3.**
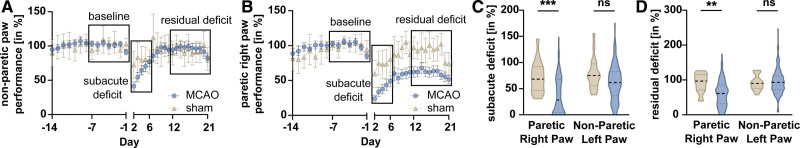
**Assessing the sensorimotor deficit after stroke. A**, Nonparetic left and **B**, paretic right paw performance over time, bars depict 95% CI. Performance on days −7 to −1 were averaged and taken as a baseline (first black rectangle). Data from days 2 to 6 were summarized and referred to as subacute deficit (second rectangle) while data from days 12 to 21 are referred to as residual deficit (third rectangle). **C**, Subacute and **D**, residual deficit of the nonparetic and paretic paw in sham (brown plot) and middle cerebral artery occlusion (MCAO; blue plot) animals. Dashed lines indicate medians, dotted line indicate quartiles. Mixed-effects model followed by Šidák post hoc tests for multiple comparison were used to test effect of group and side.

Performance in the prediction cohort peaked after ≈7 days (Figure 3A and 3B). Baseline performance of the prediction cohort is reported in the Supplemental Material and Methods. In the phase of the subacute deficit, the left (nonparetic) side showed 61.6% (56.1–67.1) performance, while the right (paretic) side showed 37.6% (31.9–43.3; Figure 3C). In sham animals, performance on nonparetic side was 78.4% (63.5–93.3) and 72.2 (57.3–87.2) on the paretic side. Mixed-effects analysis exhibited a side-group interaction (*F*[1164]=4.2; *P*=0.0423). MCAO mice performed significantly worse than sham on the paretic paw (least square mean difference [MD], 34.6pp; *t*=4.1; df=328; *P*=0.0001). Nonparetic sides did not differ between groups (least square MD, 16.8 pp; *t*=2.0; df=328; *P*=0.0937). To demonstrate side-specificity of the motor-functional deficit in MCAO mice, paired *t* tests were performed on paretic and nonparetic paws. The paretic paw performed significantly worse (MD, 24.0%; 18.3–29.7; *t*=8.4; df=147; *P*<0.0001; Figure S7A). In the period of residual deficit, the performance of nonparetic paw in MCAO animals was 95.4% (89.5–101.2) and 60.9% (54.9–66.8; Figure 3D) on the paretic side. Sham animals showed a performance of 92.4% (81.8–103.0) on the nonparetic side and 91.6% (78.5–104.7) on the paretic side. Mixed-effects analysis demonstrated side-group interaction (*F*[1164]=9.4; *P*=0.0026). MCAO animals showed worse paretic paw performance compared with sham (least square MD, 30.7 pp; *t*=3.5; df=328; *P*=0.0011). Nonparetic sides did not differ significantly (least square MD, 3.0 pp; *t*=0.4; df=328; *P*=0.9302). Finally, we analyzed the effects of the general well-being (neuroscore, weight loss on day 2) of the animals on functional outcome and found poor correlation to the degree of either subacute or residual deficit and worse prediction accuracies compared with imaging- or performance-based prediction (Figures S8 through S11).^25^

### Prediction of Subacute Deficit From Acute MR Imaging

**Figure 4.**
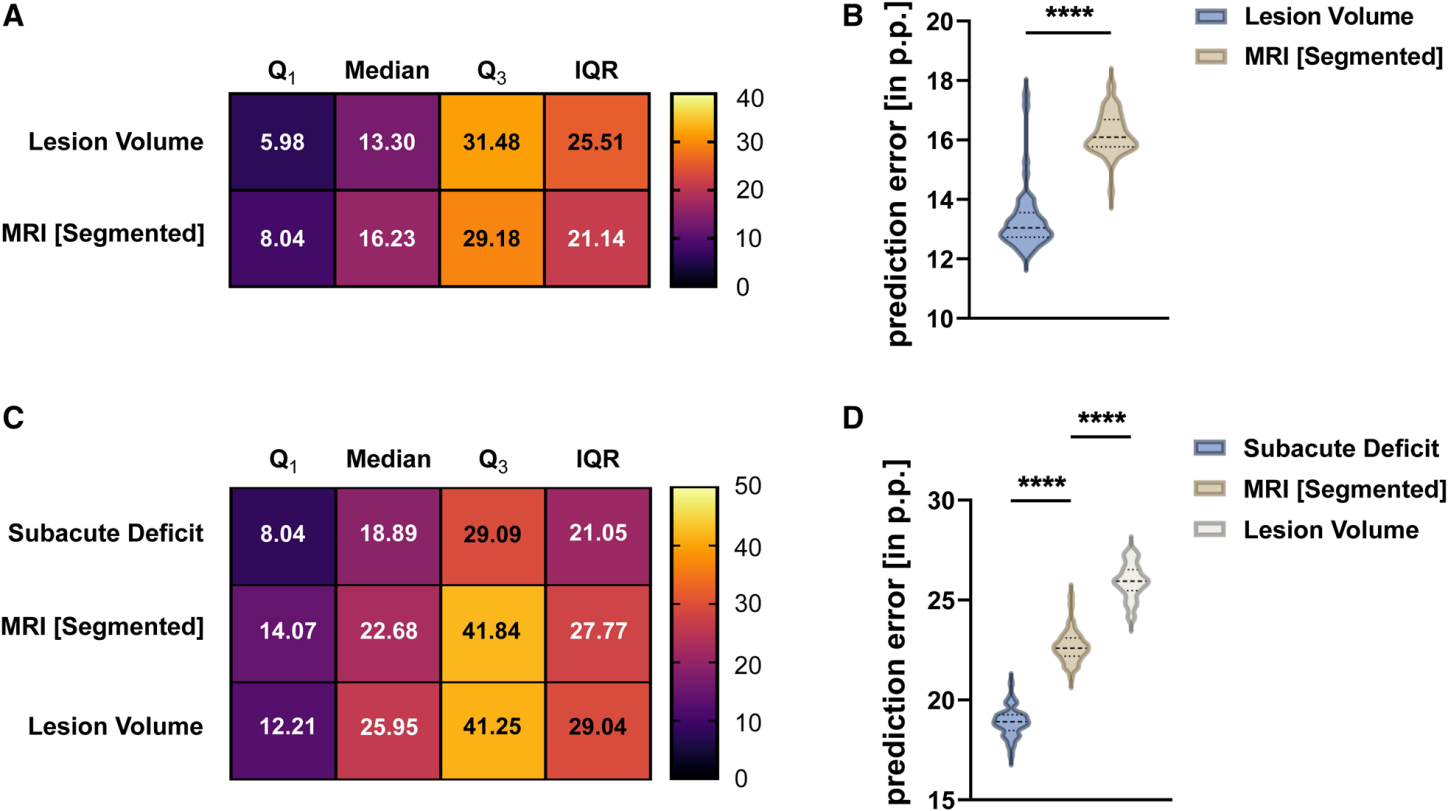
**Comparison of predictors of the subacute and residual deficit. A** and **B**, Comparison of the prediction accuracy of the 2 predictors of the subacute deficit. Averaged Q_1_, median, Q_3_, and interquartile range (IQR) were calculated using the 50 independent models for each predictor. Dashed lines indicate median, dotted lines indicate quartiles. Unpaired *t* test was used to compare prediction error (PEs). Small PE indicates small deviation between predicted and achieved performance. **C** and **D**, Comparison of prediction accuracy of the 2 imaging-based parameters and the subacute deficit for the prediction of the residual deficit. Again, 1-way ANOVA followed by Tukey test for multiple comparison was used. MRI indicates magnetic resonance imaging.

Using random forest and early noninvasive MRI data, subacute deficit prediction models were created. PE was 13.3 pp (13.0–13.7; Q_1_, 6.0; Q_3_, 31.5; interquartile range [IQR], 25.5) for the lesion volume and 16.2 pp (16.0–16.4; Q_1_, 8.0; Q_3_, 29.2; IQR, 21.1) for the segmented MRI (Figure 4A and 4B). Unpaired, two-tailed *t* test revealed that PE was significantly smaller when using the lesion volume as a predictor compared with the segmented MRI (MD, 2.9 pp [2.6–3.3]; *t*=16.6; df=98; *P*<0.0001).

### Prediction of Residual Deficit From Subacute Deficit and MRI

PE was 18.90 pp (18.70–19.09; Q_1_, 8.04; Q_3_, 29.09; IQR, 21.05) for the subacute deficit, 22.68 pp (22.46–22.90; Q_1_, 14.07; Q_3_, 41.84; IQR, 27.77) for the segmented MRI and 25.95 pp (25.71–26.29; Q_1_, 12.21; Q_3_, 41.25; IQR, 29.04) for the lesion volume (Figure 4C and 4D). One-way ANOVA showed predictors differed significantly (*F*[2147]=1032; *P*<0.0001). After multiple comparisons were corrected using Tukey test, the PE of the subacute deficit was significantly smaller than the PE of the segmented MRI (MD, 3.79 pp [4.15–3.42]; *P*<0.0001) and the lesion volume (MD, 7.06 pp [7.43–6.69]; *P*<0.0001). Segmented MRI outperformed the prediction accuracy of the lesion volume (MD, 3.28 pp [3.65–2.91]; *P*<0.0001).

### Identification of Anatomic Regions Associated With Long-Term Outcome

**Figure 5.**
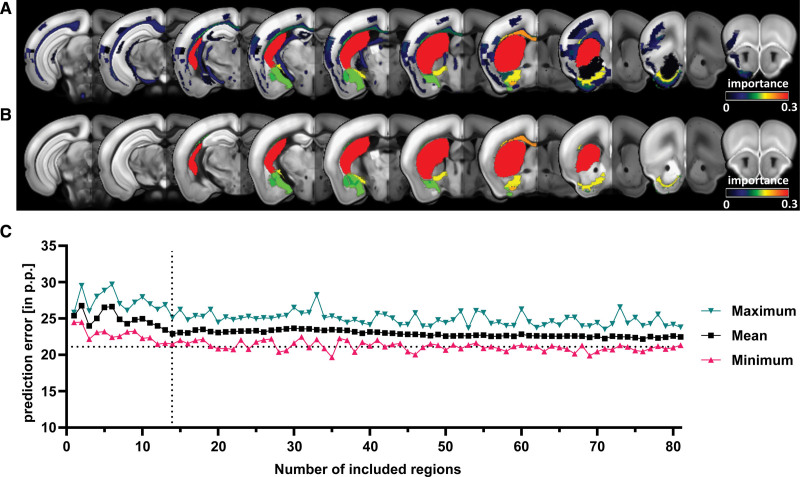
**Identification of anatomic regions that drive imaging-based prediction of functional outcome. A**, Coronal slices depicting the importance of the 81 regions calculated from our random forest models (RF). Only regions with an importance level >0 are shown **B**, Visualization of the importance level of the 14 anatomic regions that were included in the prediction model that resulted in a local minimum of prediction error (PE). **C**, Prediction of residual deficit was performed using an increasing number of regions in the segmented magnetic resonance imaging. PE as well as maximum and minimum PE are depicted. Dashed lines at x=14 and y=21.5 denote the first local minimum of PE when including only 14 regions.

Using out-of-bags, each MRI region’s contribution to long-term outcomes was quantified. 81 locations had above-zero out-of-bag predictor importance, contributing more than random data (Figure 5A and 5B; Table S4). Among those were the caudoputamen, primary somatosensory area, external capsule, and corticospinal tract. Next, random forest models were trained using increasing numbers of MRI regions, starting with the most important. The models’ PE gradually reduced with more MRI regions included (Figure 5C). However, PE rapidly plateaued, and adding more regions only minimally increased accuracy. After the inclusion of the first 14 anatomic regions, PE displayed a relevant local minimum (22.9 pp).

### Validation in an Independent Cohort

**Figure 6.**
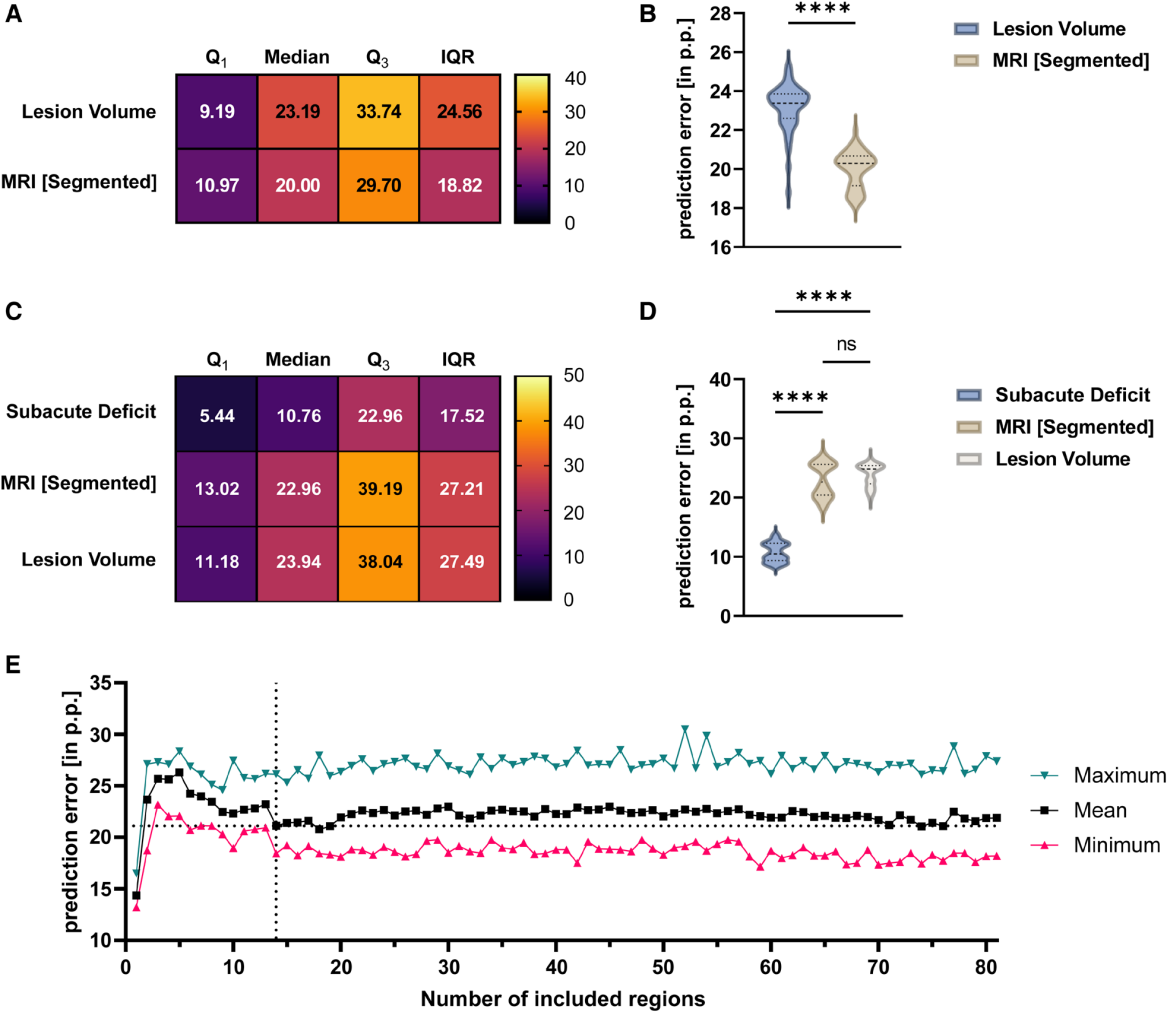
**Validation of prediction models in an independent cohort. A** and **B**, Comparison of the prediction accuracy of the 2 predictors of the subacute deficit. Dashed lines indicate median, and dotted lines indicate quartiles. Unpaired *t* test was used to compare prediction errors (PEs). While PEs were comparable to the ones seen in our prediction cohort, subacute deficit was best predicted when using the segmented magnetic resonance imaging (MRI). **C** and **D**, Comparison of prediction accuracy of the 2 imaging-based parameters and the subacute deficit for the prediction of the residual deficit. Again, the subacute deficit was the best predictor of the residual deficit, followed by the segmented MRI and the lesion volume. **E**, Analogous to the prediction cohort, prediction of residual deficit was performed using an increasing number of regions in the segmented MRI. Dashed lines at x=14 and y=21.5 denote the local minimum of PE that had previously been observed in the prediction cohort.

Finally, we tested our prediction models on the independent replication cohort. Imaging and behavioral analyses are included in Supplemental Material and Methods. For prediction of subacute deficit using lesion volume, mean PE was 23.2 pp (22.9–23.5) and 20.0 pp (19.7–20.3) using segmented MRI (Figure 6A and 6B). In contrast to results in the prediction cohort, the segmented MRI showed significantly smaller PEs compared with lesion volume (MD, 3.2 pp [3.6–2.8]; *t*=14.9; df=98; *P*<0.0001). The PE for the residual deficit using the subacute deficit was 10.8 pp (10.3–11.2), 23.0 pp (22.2–23.8) for the segmented MRI, and 23.9 pp (23.4–24.5) for the lesion volume (Figure 6C and 6D). One-way ANOVA showed predictors differed significantly (*F*[2147]=19.0; *P*<0.0001). PE of the subacute deficit was significantly smaller than the PE of the segmented MRI (MD, 12.2 pp [13.2–11.2]; *P*<0.0001) and the lesion volume (MD, 13.2 pp [14.2–12.2]; *P*<0.0001). Segmented MRI provided slightly smaller PE than lesion volume (MD, 1.0 pp [2.0–0.1]; *P*=0.0668). Finally, we tested the prediction accuracy when including an increasing number of anatomic regions. Only regions we had identified to be relevant for the prediction of the residual deficit in our prediction cohort were included. Here, lesion percentage in the caudoputamen alone yielded lower PEs than the entire segmented MRI or lesion volume. When adding regions, PEs spiked and then slowly decreased. After incorporating 14 regions, we again found a local minimum of PE (Figure 6E), replicating our finding from the prediction cohort.

## DISCUSSION

In this retrospective study, we systematically compared different predictors of subacute and long-term functional outcomes after stroke in a large cohort of mice. We worked with a cohort that underwent 45 minutes of MCAO and included different genotypes and 2 different surgeons, thereby embracing the resulting heterogeneity of stroke volumes and functional outcomes. This step widens the dynamic range of our prediction tool and integrates translationally relevant levels of variability into our experimental design. Although these levels are not inherent to the MCAO model if performed in young adult, male C57Bl/6 wildtype mice, the selected approach expands the applicability in studies with heterogeneity of various sources. We used MRI-derived parameters such as the lesion volume and lesion topology, as well as early functional testing to predict poststroke performance in a pellet reaching task. Studies with large sample sizes have transformed preclinical MRI research and corresponding open data repositories have proven extremely valuable, for example, in the context of robust functional networks measured by resting-state fMRI.^26^ Predictors of poststroke outcomes, such as imaging-derived parameters or functional data, have previously been extensively studied in humans.^2–6,8^ While 1 preclinical study on predictors of stroke outcome in a porcine MCAO model exists, evidence on prediction of stroke outcome in mice is scarce, despite rodents being used most commonly as model animals in stroke studies.^12,27^

A side-specific disability of the forepaw was detected over 3 weeks post-surgery. This indicates that performance was governed by the stroke and not by general sickness behavior. We observed distinct trajectories of functional recovery. The differentiation between recoverees and nonrecoverees is comparable to what can be observed in humans.^14^ The resemblance to the human recovery process supports the predictive value of stroke modeling in mice and the case for implementing sophisticated behavioral tests that involve a longer phase of training before stroke for assessing long-term impairments.^28^ One should consider that food restriction during staircase training and testing may affect mortality and lesion volume.^29^

The lesion volume was the best predictor for the subacute deficit after stroke in mice as measured by PE in our prediction cohort. In the replication cohort, it was outperformed by the segmented MRI, which included information about the lesion topology. While PEs remained low, this is clearly a difference between the 2 cohorts. However, we also observed differences in stroke size and topology between the 2 cohorts. In our replication cohort lesion volumes were bigger and cortical regions accounted for higher proportions of the lesion volume compared with the prediction cohort. While it has previously been stated that lesion volume is a good predictor for acute stroke outcome, our data suggest that in mice with greater cortical lesions taking into account topological information provides a more accurate prediction of early functional outcome.^6^ For the long-term outcome, in the prediction cohort, the segmented MRI provided significantly better prediction accuracies compared with the lesion volume. This indicates that for functional recovery, lesion topology is more relevant than lesion volume, which again is in line with clinical data.^11,12^ It should be noted that in the 45-minute MCAO model, lesion volume and lesion topology are not fully independent predictors. Larger ischemic lesions typically include cortical regions while smaller lesions are restricted to subcortical structures. This phenomenon can be observed in the results of our replication cohort, where the difference between the segmented MRI and the lesion volume was only marginal but trending toward significance.

Following up on the influence of lesion topology, we identified anatomic regions that drive imaging-based prediction of the residual deficit. We found that considering only a subset of anatomic regions can explain functional long-term outcomes. Prediction accuracy displayed a local maximum after 14 regions had been added to the prediction model. Among those regions were both, cortical and subcortical regions typically affected by a MCAO stroke, such as the caudoputamen and the primary somatosensory area. Evidently, the caudoputamen is part of the lesion core in the transient occlusion model but also serves as the structure that best predicts the performance. Further, white matter regions such as the corpus callosum and the fiber tracts were shown to be of particular importance for the prediction of long-term outcomes. This is in line with previous studies in humans suggesting that lesions of the corticospinal tract seem to be more important to both early and long-term functional outcome than lesion volume.^30,31^ These findings could be replicated in our independent replication cohort, where a local minimum of PE could be observed after including the same 14 regions. Again, lesions in the caudoputamen had a major impact on prediction accuracies, outperforming both cortical gray and white matter structures.

Imaging-based predictors offered good estimates of poststroke long-term outcome, but the best predictor of the residual deficit was the degree of the subacute deficit, again confirming studies on predictors of poststroke outcome in humans.^32^ Last, we found that independent of the chosen model, the prediction accuracy for both the subacute and residual deficits depended on the degree of the deficit. In line with clinical data, we found that severe residual deficits are more difficult to predict than moderate-to-mild deficits.^13,14^

### Study Limitations

We have restricted our prediction tools to 45 minutes of MCAO. Because mice from each study were only operated on by 1 surgeon, the fact that the surgeon was identified as an additional covariate must be considered with caution, as we cannot rule out experiment-specific factors like genetic drifts, time of year, or stress levels. We did not replicate our results using different behavioral readouts. MR imaging data acquired at a later time point might have improved prediction accuracy. However, since mice have a small window of opportunity for interventions, late MR imaging cannot be utilized to guide treatment decisions.^33^ Manually delineating lesion masks is a further limitation. Although manual lesion segmentation is still the gold standard, well-documented, and publically accessible stroke T1-weighted MRI datasets enable completely automated algorithms for MRI stroke lesion segmentation in humans.^34–36^ T2-weighted MRI in rodent stroke models shows good agreement between manual segmentation by multiple raters and fully automated techniques.^37^ These investigations advanced lesion segmentation automation.^37,38^ FLAIR (fluid attenuated inversion recovery) MRI fluid signal nulling might have enhanced CSF-ischemic lesion discrimination (cerebrospinal fluid). However, 24 hours after MCAO, CSF has a stronger RARE (rapid acquisition with relaxation enhancement) signal than the lesion and the lateral ventricles can be accurately identified.^23^ During the subacute deficit phase, animals’ unspecific sickness behavior may have affected performance. However, our behavioral paradigm revealed stroke-specific motor-functional impairments since we observed side-specific deficits between sham and MCAO, as well as paretic and nonparetic paw. There were no video recordings of the testing sessions to account for changes in kinematics, but the whole behavioral data set has been posted to the repository connected to this study. Finally, while larger data sets usually perform better when training machine learning models, the random forest method works well with sample sizes comparable to our data set, as shown when the method was first introduced and in later comparative studies.^24,39^

### Conclusions

We found and described mouse poststroke outcome predictors. The concordance with clinical data strengthens the usability of mice as model animals in preclinical studies. Prediction of outcomes in intervention studies using noninvasive MRI measurements or motor-functional data should be utilized to compare expected and achieved motor-function outcomes, select mice with particularly poor predicted outcomes, and guide treatment or intervention options. This will assist in bridging the translational gap and increase preclinical rodent stroke research reproducibility.

## ARTICLE INFORMATION

### Acknowledgments

Janet Lips coordinated experiments and contributed with magnetic resonance imaging (MRI), handling, and behavioral evaluation; Larissa Mosch (behavior, histology), Monika Dopatka (surgeries), and Marco Foddis (surgeries, behavioral assessment, and MRI) all provided excellent technical assistance. Figures were created with biorender.com.

### Sources of Funding

Funding was provided by the Deutsche Forschungsgemeinschaft (DFG, German Research Foundation) to Drs Hoffmann and Harms (project number, 417284923), to Dr Boehm-Sturm (project number, 428869206), to Drs Wenger, Endres, and Harms (project number 424778381–TRR 295) and NeuroCure (EXC−2049 to —390688087) and the German Federal Ministry of Education and Research (BMBF, Center for Stroke Research Berlin 01EO1301) to Drs Dirnagl, Boehm-Sturm, and Harms, and to Dr Boehm-Sturm by the BMBF under the ERA-NET NEURON scheme (01EW1811). Dr Kuffner received funding from the DFG Graduate School 203. This work was supported by Charité3^R^| Replace–Reduce–Refine and partly by the Fondation Leducq to Drs Endres and Harms. Drs Knab and Eggers received a scholarship from the Berlin Institute of Health, Berlin. Dr Euskirchen, Wenger, and Hoffmann are participants in the Charité Clinical Scientist Program funded by the Charité – Universitätsmedizin Berlin and the Berlin Institute of Health and Dr Wenger is a Freigeist Fellow with support from the Volkswagen Foundation. In addition, this work was supported by DFG (Project number 73500270 and 413848220) and ERA-NET NEURON EBio2, with funds from BMBF 01EW2004 to Dr Dreier. Dr Endres received funding from DFG under Germany´s Excellence Strategy—EXC-2049—390688087, Collaborative Research Center ReTune TRR 295- 424778381, BMBF, DZNE, DZHK, EU, Corona Foundation, and Fondation Leducq. am received funding from DFG and Fondation Leducq.

### Disclosures

Dr Endres reports grants from Bayer and fees paid to the Charité from Abbot, Amgen, AstraZeneca, Bayer Healthcare, Boehringer Ingelheim, BMS, Daiichi Sankyo, Sanofi, Novartis, Pfizer, all outside the submitted work. Dr Wenger holds patents on neuromodulation technologies that are unrelated to this work. All remaining authors certify that they have no affiliations with or involvement in any organization or entity with any financial interest or nonfinancial interest in the subject matter or materials discussed in this article. No funding was received to assist with the preparation of this article.

### Supplemental Material

ARRIVE Checklist

Expanded Materials and Methods

Supplemental Data

Figures S1–S15

Tables S1–S9

Reference 40

## Supplementary Material


