# Supplementary material for: Prediction of Stroke Outcome in Mice Based on Noninvasive MRI and Behavioral Testing
Source: Stroke. 2023 Sep 25;54(11):2895–905. doi: 10.1161/STROKEAHA.123.043897 (PMC10589430; doi:10.1161/STROKEAHA.123.043897)
Supplement: Supplementary file 1 [file str-54-2895-s001.pdf]

SUPPLEMENTAL MATERIAL

Prediction of Stroke Outcome in Mice Based on Non-Invasive MRI and Behavioral Testing

Felix Knab, Stefan Paul Koch, Sebastian Major, Tracy D. Farr, Susanne Mueller, Philipp Euskirchen, Moritz Eggers, Melanie T.C. Kuffner, Josefine Walter, Daniel Berchtold, Samuel Knauss, Jens P. Dreier, Andreas Meisel, Matthias Endres, Ulrich Dirnagl, Nikolaus Wenger, Christian J. Hoffmann, Philipp Boehm-Sturm, Christoph Harms

SUPPLEMENTAL METHODS

Experimental Design

In the *prediction cohort*, 148 mice received MCAO surgery (female: n = 40, male: n = 108). For comparison of functional outcome, 18 mice underwent sham surgery (female: n = 7, male: n = 11). In the *replication cohort*, 37 mice underwent MCAO surgery (female: n = 10, male: n = 27). For behavioral analysis, 12 mice received sham surgery (male: n = 12). We had initially only recruited data for the prediction cohort. To thoroughly test our prediction tools, we pooled data from two additional studies, making the data from the *replication cohort* entirely independent from the *prediction cohort.* A total of 15 different genotypes were used (Table S1). If activation of genotype was required (n = 50), either Tamoxifen (n = 43) or vehicle (n = 7) was used. A total of 19 mice were part of studies that included therapeutic treatment after the MCAO surgery of which 10 received an active agent and 9 received a placebo control (Table S1). Four exclusion criteria were pre-specified: **1)** In the third week of testing, mice performed below 20% of the average in the staircase test (n=45); **2)** no visible infarct in MRI (n=29); **3)** surgical death or humane euthanasia before study end (n=101) and **4)** incomplete behavioral data sets after the data merging process (n=47). Further, some mice were excluded because they had received 30 minutes of MCAO or had previously undergone surgery (n=11). Finally, in one study there was an exclusion criteria specific to the study context (misfolded histological slices, n=2). Following this exclusion process, 215 mice (*prediction cohort:* n=166, MCAO: n=148, sham: n=18; *replication cohort:* n=49, MCAO: n=37, sham: n=12) were included in the study. Mice were housed in groups of five in a 12 h day cycle and were food restricted but had free access to drinking water. Mice were weighed every day to control weight. They were familiarized with the pellets used in the behavioral task and with the test apparatus within the first week of handling by putting pellets in their cage. Eight days later, training sessions began (Figure 2A). Mice were initially food restricted for 20 h before training sessions; each session lasted 30 minutes. If weights stabilized around 85% of their free-feeding weights, food restriction was adjusted and increased to up to 22 h. After the training session, mice had individual access to standard lab chow diet to avoid food hoarding of the dominant mice. Mice were trained for fourteen days until average performance stabilized. We tested the mice for another seven consecutive days and the average performance during this period was used to normalize post-stroke pellet reaching. Performance was expressed as a percentage of average pre-stroke performance. Mice were randomized and allocated to either MCAO or sham surgery. 24 hours prior to the surgery mice had free access to food. After the surgery, mice had another two hours of access to food before the previously described cycle of food deprivation began. After MCAO lab chow diet pellets were homogenized using water to assist with feeding. 24 hours after the surgery MR imaging was performed. 48 hours after the surgery testing resumed for another twenty-one days at which point the animals were sacrificed.

Staircase Test of Skilled Reaching

The staircase test of skilled reaching in mice uses a testing box consisting of two stairs on the right and left side that were baited with up to two food pellets. The pellets consisted of sucrose (20 mg Dustless Precision Pellets®, Bio-Serv). Only one pellet was put onto the two highest stairs as mice would use their tongue to obtain these. In one study, number of pellets per side was 8 (n = 19), in one study it was 16 (n = 19) and in the remaining studies number of pellets per side was 14 (n = 147). For optimal comparability of motor-function, the individual performance was normalized to the pre-stroke performance. Therefore, average percentage of retrieved pellets was calculated for the period of baseline testing. Performance was then calculated as a percentage of the respective baseline performance. Subgroups were based on the severity of the of the paretic paw: mild (performance >80%), moderate-mild (60–79%), moderate (40–59%), moderate-severe (20–39%) and severe deficit (0–19%). Both *subacute* and *residual deficit* were used to define subgroups, depending on the analysis context (Figures 2B–D).

Effect of Study-Related Variables on Functional Outcome

The effect of study-related concomitant variables (genotype, activation of genotype, therapeutic treatment, experiment number, sex, age at surgery, time to MCA occlusion and surgeon) on performance were investigated using separate analysis of variance linear mixed-effects models for *subacute* and *residual deficit* (response variable), respectively. For *subacute deficit*, a mixed-effects ANOVA revealed no significant effect of study-related variables (genotype: F(11, 149) = 0.902, p = 0.540; activation of genotype: F(1, 149) = 0.849, p = 0.358; therapeutic treatment: F(4, 149) = 0.279, p = 0.891; experiment number: F(5, 149) = 0.955, p = 0.447; sex: F(1, 149) = 0.055, p = 0.814; age: F(1, 149) = 0.011, p = 0.917; time to MCA occlusion: F(1,149) = 0.919, p = 0.339). The surgeon proved to not be an independent variable as each experiment was performed by one surgeon. Therefore, experiment number and surgeon were identical variables. For *residual deficit*, no significant effect of study-related variables was found (genotype: F(11, 149) = 1.61, p = 0.102; activation of genotype: F(1, 149) = 3.503, p = 0.063; therapeutic treatment: F(4, 149) = 0.725, p = 0.576; experiment number: F(5, 149) = 1.60, p = 0.164; sex: F(1, 149) = 0.007, p = 0.931; age: F(1, 149) = 0.172, p = 0.679; time to MCA occlusion: F(1,149) = 0.227, p = 0.635).

MCAO Surgery

Mice were anaesthetized with 1.5-3.5 % isoflurane and maintained with 1.0 % to 2.5 % in a mixture of 30 % O_2 and 70 % N_2O. During the procedure, body temperature was measured and maintained at 36 to 37.5 °C using a heating blanket and rectal probe. After disinfection of the skin, a midline neck incision was made and the common carotid artery (CCA) was carefully dissected from the surrounding nerves and ligated using 7.0 suture thread. The external carotid artery (ECA) was separated and ligated and the internal carotid artery (ICA) and the pterygopalatine (PA) arteries were clipped. A 180 µm diameter Doccol® filament was introduced into the common carotid artery, secured with a suture, the clip opened, and the filament advanced. The mice were allowed to recover in a heated cage at thermoneutral temperature (30-31 °C) for the duration of the MCAO. After 45 minutes, the mice were re-anaesthetized and the filament was withdrawn. The skin was sutured. As for the sham procedure, the filament was inserted to occlude the MCA and withdrawn immediately. For pain relief, Bupivacaine gel was topically applied in the vicinity of the wound. Standardized operation procedures were followed to minimize differences in total surgery duration in between the mice (doi: 10.1038/npre.2010.3492.2). Humane endpoints were reached as described in the section “2.2.5 Humane endpoint criteria” by Mei et al. 2019 (doi:10.14573/altex.1812231).

MRI

T2-weighted (T2w) images were acquired 24 h post stroke surgery on a 7 T MR scanner (Bruker, Ettlingen, Germany) and a 20 mm inner diameter transmit/receive volume coil (RAPID Biomedical, Rimpar, Germany). Anesthesia was achieved using 1-2 % isoflurane in a 70:30 nitrous oxide:oxygen mixture, and body temperature and respiration rate were monitored with MRI compatible equipment (Small Animal Instruments, Inc., Stony Brook, NY, USA). A 2D RARE sequence was used (repetition time/echo spacing/effective echo time=4.2 s/12 ms/36 ms, RARE factor=8, slice thickness 0.5 mm, FOV = $(25.6\ \text{mm})^2$, image matrix=256x196 zerofilled to 256x256, 4 averages, acquisition time 6:43 min). The lesion was manually delineated by an experienced researcher using ANALYZE 5.0 software (AnalyzeDirect, Overland Park, KS, USA). MRI data were registered on the Allen Mouse Brain Atlas using the in-house developed MATLAB toolbox ANTx2 (https://github.com/ChariteExpMri/antx2). Incidence maps expressing percentage of animals with a lesion in a voxel were plotted for each group in atlas space and edema-corrected *lesion volume* was calculated as described previously.[21] Percentage of damage in a given region was calculated for each individual mouse using the delineated lesion masks and is referred to as *segmented MRI*. The rater of the MRI was blinded to both the type of intervention and the results of the staircase test.

Statistics

Behavioral data were analyzed using GraphPad Prism software, version 9.3.0. (La Jolla, CA, United States). A mixed-effects model followed by Šidák's post-hoc tests for multiple comparison was used to analyze the effect of side (right, paretic paw or left, non –paretic paw) and group (sham or MCAO) on performance. Alpha level was 0.05, mean difference and 95 % confidence intervals are reported. For the analysis of stroke volumes and deviation from group mean in percent when applying different occlusion times Kruskal-Wallis test was used, followed by Dunn's test to correct for multiple comparisons. For the comparison of lesion volumes and percentage of lesion within the caudoputamen in the *prediction* and *replication cohort*, Mann-Whitney test was used. For the comparison of *lesion volume* in different functional subgroups as well as comparison of different prediction models and their effect on the prediction error, either unpaired t-test or one-way ANOVA was used. ANOVA was followed by Tukey's test for multiple comparison. When the effects of the prediction model and the severity grades on the prediction error were analyzed, two-way-ANOVA followed by Tukey's test were performed.

Division of Prediction Cohort into Training and Testing Group

Since long-term outcome was the most important outcome measure in this study, we ensured a comparable distribution of the *residual deficit* in both groups. To achieve this, we first sorted the animals from the *prediction cohort* according to the *residual deficit*. Subsequently two animals from each consecutive triplet were randomly assigned to the training group and one to the test group, resulting in 98 animals in the training data set and 50 animals in the test data set. Analysis of behavioral data was done for the three groups (training cohort, *testing cohort,* sham animals) analogous to analysis of the entire cohort. Mean performance in the time period of *subacute deficit* was 58.56 % (51.81 to 65.30) on the non-paretic side and 36.50 % (29.29 to 43.71) on the paretic side of the *training cohort* and 67.56 % (58.24 to 76.89) on the non-paretic side and 39.73 % (30.60 to 48.85) on the paretic side of the *testing cohort* (Figure S12). Mixed-effects analysis revealed that the factors side and group had a significant impact on the performance (Side: $F(1, 164) = 30.29$, $p < 0.0001$; Group: $F(2, 164) = 7.09$, $p = 0.0011$). After correcting for multiple comparisons using

Šidák's test, performance of the paretic paw was significantly lower in the *training* (t = 4.128, DF = 328, p = 0.0003) and *testing cohort* (t = 3.501, DF = 328, p = 0.0032) when compared to sham. There was no significant difference between *training* and *testing cohort* (t = 0.5506, DF = 328, p = 0.9947). There was also no significant difference between *training* (t = 2.293, DF = 328, p = 0.1275) or *testing cohort* (t = 1.167, DF = 328, p = 0.8133) on the non-paretic side when compared to sham. Mean performance in the time period of *residual deficit* was 96.10 % (88.57 to 103.63) on the non-paretic side and 60.17 % (53.10 to 67.25) on the paretic side of the training cohort and 93.86 % (85.11 to 102.64) on the non-paretic side and 62.26 % (51.35 to 73.17) on the paretic side of the *testing cohort*. Mixed-effects analysis revealed that there was a statistically significant interaction between the effects side and group (F(2, 165) = 4.875, p = 0.0088). After correcting for multiple comparisons using Šidák's test, performance of the paretic paw was significantly lower in the *training* (t = 3.491, DF = 330, p = 0.0033) and *testing cohort* (t = 3.046, DF = 330, p = 0.0150) when compared to sham. There was no significant difference between *training* and *testing cohort* (t = 0.3449, DF = 330, p = 0.9996). There was also no significant difference between *training* (t = 0.4129, DF = 330, p = 0.9989) or *testing cohort* (t = 0.1542, DF = 330, p >0.9999) on the non-paretic side when compared to sham.

Development of Prediction Models with Machine Learning

For the development of prediction models with machine learning we divided the whole *prediction cohort* into a training (2/3 of the animals) and a testing cohort (1/3 of the animals). Using the training data, we tested several regression methods in the regression learner app in MATLAB (version 2021a) and chose random forest, which showed best performance on training data. We decided to train 50 independent models for each predictor additionally performing automated Bayesian hyperparameter optimization with 100 iterations for each single model.

Estimation of Importance of Anatomical Regions Using Out-of-Bag Observations

When training a random forest model, each tree is trained on a randomly selected subset of the training data, leaving out an average of one-third of the observations, which are then referred to as "out-of-bag" observations. These can be used to compute the importance of the variables, since they can serve as the test data set for the particular tree. First, the prediction error for each tree is computed using only the out-of-bag observations, then all values of one variable are permuted and the error is computed again for the out-of-bag observations, which now contain randomly permuted values for this particular variable. Subsequently, the difference between the errors before and after permutation is calculated. This procedure is repeated for each variable used to partition the nodes in the current tree. After each tree is processed, the mean and standard deviation of the error differences across all trees are calculated for each variable. Finally, the importance of each variable is calculated as the mean of the error differences divided by their standard deviation, which can also be considered as the signal-to-noise ratio. The out-of-bag predictor importance was estimated for each of the 50 random forest models trained on *segmented MRI* data. This was followed by calculation of the median value across all models and sorting of the regions by importance in descending order.

SUPPLEMENTAL RESULTS

Report On Mortality

Out of 408 mice that received MCAO, a total of 99 (24.3%) mice died or were euthanized at a humane endpoint before reaching the end of the study. 4/408 (1.0%) mice died before the surgery. 12/408 (2.9%) mice died during the surgery, and 5/408 mice (1.2%) died within 24 hours of the surgery. 19/408 (4.7%) mice died after more than 24 hours after the surgery, and 59/408 (14.7%) were euthanized at a humane endpoint (Figure S13). Out of the 42 mice that received a sham surgery, two mice died after the surgery. Mortality rates are in line with previous reports on longer occlusion times.[40]

MRI Characterization and Lesion Topology in Prediction and Replication Cohort

Incidence maps were generated for the *prediction* and *replication cohort* (Figure S4A and S4B). Average *lesion volume* was 25.00mm^3 (21.46–28.54) in the *prediction cohort* and 30.43mm^3 (23.39-37.46) in the *replication cohort* (Figure S4C). Mean lesion volumes trended to be significantly larger in the *replication cohort* (actual difference: 13.57 mm^3, U=2186, p=0.058). Lesion volume in the caudoputamen accounted for 51.58% (46.42-56.75) in the *prediction cohort* but for only 38.15% (29.75-46.55) in the *replication cohort* (Figure S4D). Lesion volume in the caudoputamen accounted for a significantly larger proportion of the total stroke volume in the *prediction cohort* compared to the *replication cohort* (actual difference: 22.26%, U=1989, p=0.011). Incidence maps and stroke volumes were also generated and calculated for the functional subgroups of the *prediction cohort* (Figure S5A). Classified by degree of residual deficit, mean lesion volume was 14.03 mm^3 (9.82–18.25) in mild, 17.17 mm^3 (12.52–21.82) in moderate to mild, 26.29 mm^3 (16.86–35.73) in moderate, 33.62 mm^3 (24.04–43.20) in moderate to severe, and 47.23 mm^3 (36.79–57.68) in severe residual deficit mice (Figure S5B). One-Way ANOVA showed a significant difference in mean lesion volumes between functional subgroups (F(4,143)=14.14, p<0.0001; full report in Table S5).

Baseline Performance of the Prediction Cohort

The average percentage of retrieved pellets during *baseline* testing was 49.3% (47.2–51.3) on the left (later non-paretic) side and 46.3% (44.2–48.4) on the right (later paretic) side in the MCAO group and 45.8% (37.4–54.2) and 47.1% (39.0–55.3) on the left and right sides in the sham group (Figure S6). Mixed effects analysis showed no side-group interaction (F(1,164)=1.464, p=0.2281).

Behavioral Analysis of the Replication Cohort

Behavioral and imaging data from two studies performed in 2015 and 2019 were pooled. After applying our exclusion criteria, 49 mice were included (MCAO: n = 37, sham: n = 12). Mean performance in the time period of *subacute deficit* was 70.98 % (60.93 to 81.02) on the non-paretic side and 44.21 % (35.78 to 52.65) on the paretic side (Figure S14A through S14C). In the sham animals, mean performance during the phase of *subacute deficit* was 101.15 % (93.17 to 109.13) on the non-paretic side and 103.96 % (97.89 to 110.02) on the paretic side. Mixed-effects analysis revealed that there was a statistically significant interaction between the effects side and group (F(1, 47) = 11.13, p = 0.0017). After correcting for multiple comparisons using Šidák's test, performance of the paretic paw was significantly lower in MCAO animals when compared to sham (least square mean difference = 59.74 p.p., t = 7.213, DF = 94, p < 0.0001). In the *replication cohort*, there was also a significant difference between groups on the non-paretic side (least square mean difference = 30.17 p.p., t = 3.643, DF = 94, p = 0.0009). Paired t-test was performed to test

for a difference between paretic and non-paretic paw of the MCAO. Performance in the paretic paw was significantly lower than in the non-paretic paw (MD: 26.77 %, 16.85-36.68, t = 5.473, df = 36, p<0.0001) (Figure S7B). Mean performance in the period of *residual deficit* was 85.42 % (78.26 to 92.57) on the non-paretic side and 71.44 % (61.85 to 81.04) on the paretic side. In the sham animals, mean performance during the phase of *residual deficit* was 92.39 % (81.80 to 102.98) on the non-paretic side and 91.56 % (78.45 to 104.66) on the paretic side (Figure S14A, S14B and S14D). Mixed-effects analysis showed that a significant impact of the factor group on the degree of the *residual deficit* (F(1, 47) = 30.10, p < 0.0001). After correcting for multiple comparisons using Šidák's test, performance of the paretic paw was significantly lower in MCAO animals when compared to sham (least square mean difference = 38.85 p.p., t = 5.101, DF = 94, p < 0.0001). In the *replication cohort*, there was also a significant difference between groups on the non-paretic side (least square mean difference = 23.31 p.p., t = 3.061, DF = 94, p = 0.0057).

Assessment of the Effects of General Well-Being of the Animals on Functional Outcome

To test what effect the general well-being of MCAO animals had on the functional outcome, we performed correlation analyses using weight loss on day 2 and the Modified DeSimoni Neuroscore (from here on referred to as *neuroscore*) for the assessment of general deficits and compared this to the *lesion volume*. First, using Pearson's correlation for normally distributed data and Spearman's correlation for non-parametric data was used to test if weight loss on day 2, *neuroscore* and the *lesion volume* significantly correlated to the degree of the *subacute deficit*. The correlation was statistically significant for all three factors (weight loss: r(183) = 0.253, p = 0.0005; R^2 = 0.064; *neuroscore*: r(172) = -0.327, p<0.0001, R^2 = 0.107; *lesion volume*: rho(183) = -0.618, p < 0.0001) (Figure S8). Notably, while significant, both the *neuroscore* and the weight loss on day 2 did not seem to explain much of the variation of the *subacute deficit* as indicated by small r and R^2. This became even more clear when comparing these parameters with the *lesion volume*, where rho was much higher and seemed to explain more of the variation observed in the *subacute deficit* data.
Next, we repeated these analyses for the *residual deficit*, now also including the *subacute deficit* as an independent variable. Again, the correlation was statistically significant for all four factors (weight loss: r(183) = 0.236, p = 0.0012, R^2 = 0.056; *neuroscore*: r(172) = -0.241, p = 0.0013, R^2 = 0.058,; *lesion volume*: rho(183) = -0.466, p < 0.0001; subacute deficit: r(183) = 0.736, p < 0.0001, R^2 = 0.543) (Figure S9). Again, with r and R^2 being small for both, w*eight loss* and *neuroscore*, these parameters did not seem to explain much of the variation observed in the residual deficit data. In conclusion, correlation analyses indicated that the general well-being of the animals explained only a small fraction of the variation seen in the *subacute* and *residual deficit* data.

General well-being parameters as predictors of subacute and residual deficit

Finally, we generated two new prediction models that were based on the *weight loss* on day 2 and the *neuroscore*. We compared these two new models to our previously described predictors, namely the *lesion volume*, the *segmented MRI,* and the *subacute deficit*. Notably, data on the *neuroscore* was only available for 94 % of our cohort (data available: total cohort: n = 174; training cohort: n = 99; *testing cohort*: n = 49; validation cohort: n = 37). In the *testing cohort, PE* was 23.33pp (23.09-23.57, Q_1: 13.95, Q_3: 36.58, IQR: 22.63) for the *neuroscore* and 28.26pp (28.06-28.46, Q_1: 10.66, Q_3: 42.03, IQR: 31.37) for the *weight loss* (Figure S10A and S10B) when used for the prediction of the *subacute deficit*. One-way ANOVA showed predictors differed significantly (F(3,196) = 3306, p<0.0001). After multiple comparisons were corrected using Tukey's test, the *PEs* of both the *weight loss* and the *neuroscore* were significantly higher than the *PEs* of the *segmented MRI* (MD = 12.04pp, 11.60-12.47, p<0.0001 and MD = 7.11pp, 6.67-7.54, p<0.0001,

respectively) and the *lesion volume* (MD = 14.96pp, 14.53-15.40, p<0.0001 and MD = 10.03pp, 9.60-10.47, p<0.0001, respectively). The *neuroscore* outperformed the prediction accuracy of the *weight loss* (MD = 4.93pp, 4.50-5.35, p<0.0001). In the *replication cohort*, *PE* was 20.65pp (20.47-20.82, Q_1: 13.30, Q_3: 28.18, IQR: 14.88) for the *neuroscore* and 21.98pp (21.79-22.16, Q_1: 10.33, Q_3: 35.64, IQR: 25.31) for the *weight loss* (Figure S10C and S10D) when used for the prediction of the *subacute deficit*. A One-way ANOVA showed predictors differed significantly (F(3,196) = 130.3, p<0.0001). After multiple comparisons were corrected using Tukey's test, again, the *PEs* of both the *weight loss* and the *neuroscore* were significantly higher than *PEs* of the *segmented MRI* (MD = 1.97pp, 1.52-2.43, p<0.0001 and MD = 0.64pp, 0.19-1.10, p = 0.0019, respectively) and the *lesion volume* (MD = 1.21pp, 0.76-1.67, p<0.0001 and MD = 2.55pp, 2.09-3.00, p<0.0001). The *neuroscore* outperformed the prediction accuracy of the *weight loss* (MD = 1.33pp, 0.88-1.79, p<0.0001). When used for the prediction of the *residual deficit* in the *testing cohort*, *PE* was 29.25pp (29.15-29.34, Q_1: 13.25, Q_3: 45.32, IQR: 32.07) for the *neuroscore* and 30.14pp (29.95-30.34, Q_1: 10.58, Q_3: 45.26, IQR: 34.67) for the *weight loss* (Figure S11A and S11B). One-way ANOVA showed predictors differed significantly (F(4,245) = 2285, p<0.0001). After multiple comparisons were corrected using Tukey's test, the *PEs* of both the *weight loss* and the *neuroscore* were significantly higher than *PEs* of the *segmented MRI* (MD = 7.47pp, 7.09-7.85, p<0.0001 and MD = 6.57pp, 6.19-6.95, p<0.0001, respectively) and the *lesion volume* (MD = 4.19pp, 3.81-4.57, p<0.0001 and MD = 3.29pp, 2.91-3.67, p<0.0001). The *subacute* deficit clearly outperformed both, the *weight loss* (MD = 11.25pp, 10.87-11.63, p<0.0001) and the *neuroscore* (MD = 10.36pp, 9.98-10.74, p<0.0001) as predictor of the *residual deficit*. Again, the *neuroscore* outperformed the prediction accuracy of the *weight loss* (MD = 0.90pp, 0.52-1.28, p<0.0001). In the *replication cohort*, *PE* was 24.37pp (23.89-24.86, Q_1:17.52, Q_3: 33.37, IQR: 15.86) for the *neuroscore* and 24.63pp (24.38-24.88, Q_1: 16.59, Q_3: 36.30, IQR: 19.71) for the *weight loss* (Figure S11C and S11D) when used for the prediction of the *residual deficit*. One-way ANOVA showed predictors differed significantly (F(4,245) = 494.4, p<0.0001). After multiple comparisons were corrected using Tukey's test, the *PEs* of both the *weight loss* and the *neuroscore* were significantly higher than the *PE* of the *subacute deficit* (MD = 13.87pp, 12.83-14.91, p<0.0001 and MD = 13.61pp, 12.58-14.65, p<0.0001, respectively). *PEs* of both the *weight loss* and the *neuroscore* were significantly higher than the *PEs* of the *segmented MRI* (MD = 1.68pp, 0.64-2.71, p<0.0001 and MD = 1.42pp, 0.38-2.45, p = 0.0021, respectively), but not significantly different from the PE of the *lesion volume* (MD = 0.70pp, 0.34-1.74, p = 0.347, and MD = 0.44pp, 0.60-1.48, p = 0.773).
In conclusion, for the prediction of the *subacute deficit*, in both the *testing cohort* and the *replication cohort*, *PEs* of the parameters we used as proxies for general well-being were significantly higher than our imaging-based predictors. Additionally, the *subacute deficit* and the segmented MRI outperformed the *weight loss* and the *neuroscore* as predictors of the *residual deficit*, underscoring the meaningfulness of analyzing the early functional outcome in mice.

Prediction Accuracy Depends on Severity Grade

Prediction errors were examined to better understand the relationship between severity grade and model accuracy (Figure S15). For the assessment of the prediction accuracy of the *subacute deficit* from *lesion volume* or from *segmented MRI*, severity grades were based on the subacute deficit. Two-Way ANOVA was performed to test whether severity grades and prediction model had an effect on the *prediction error*. Two-Way-ANOVA showed a statistically significant interaction between prediction model and severity grade (F(4, 490) = 682.3, p<0.0001). Simple main effect analysis revealed that severity grade (F(4, 490) = 2822, p<0.0001) and model (F(1, 490) = 47.49, p < 0.0001) had a statistically significant effect on the prediction error. For a full statistical report,

see Table S6. For the assessment of the prediction accuracy of *residual deficit*, severity grades were based on the residual deficit. Besides the two imaging based parameters, the degree of *subacute deficit* was included in the analysis. Two-Way-ANOVA showed a statistically significant interaction between predictor and severity grade ($F(8, 735) = 214.5$, $p<0.0001$). Simple main effect analysis revealed that severity grade ($F(4, 735) = 1819$, $p<0.0001$) and predictor ($F(2, 735) = 1241$, $p < 0.0001$) had a statistically significant effect on the prediction error. For a full statistical report, see Table S7. We repeated these analyses using the same models on an independent *replication cohort*. Two-Way ANOVA was performed to test whether severity grades and prediction model had an effect on the *prediction error*. Two-Way-ANOVA showed a statistically significant interaction between prediction model and severity grade ($F(4, 490) = 1231$, $p < 0.0001$). Simple main effect analysis revealed that severity grade ($F(4, 490) = 3227$, $p<0.0001$) and model ($F(1, 490) = 152.3$, $p<0.0001$) had a statistically significant effect on the prediction error. For a full statistical report, see Table S8. For the assessment of the prediction accuracy of *residual deficit*, severity grades were based on the residual deficit. Besides the two imaging based parameters, the degree of *subacute deficit* was included in the analysis. Two-Way-ANOVA showed a statistically significant interaction between predictor and severity grade ($F(8, 735) = 380.2$, $p<0.0001$). Simple main effect analysis revealed that severity grade ($F(4, 735) = 1678$, $p < 0.0001$) and predictor ($F(2, 735) = 614.5$, $p < 0.0001$) had a statistically significant effect on the prediction error. For a full statistical report, see Table S9.

SUPPLEMENTAL FIGURES

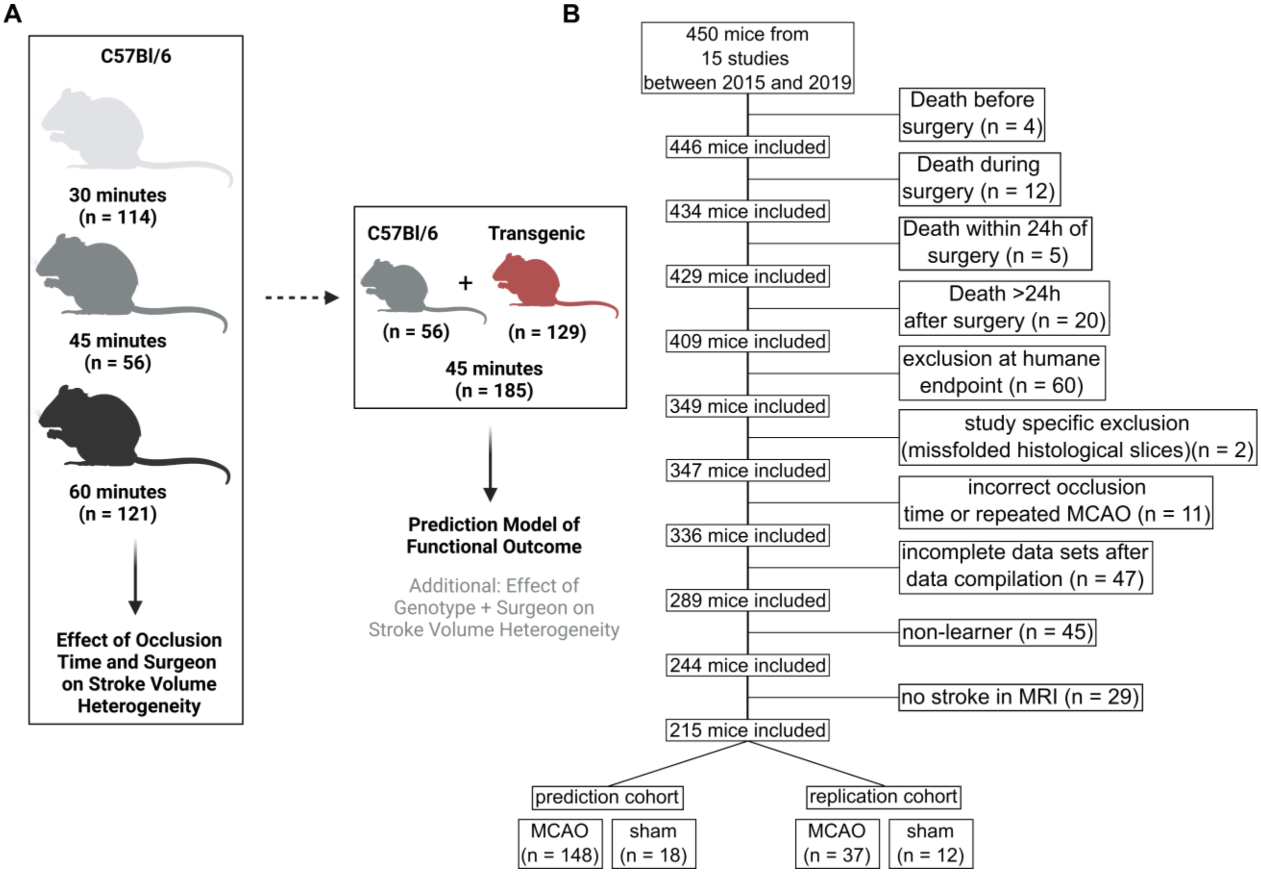

Figure S1 Overview of the study design and the inclusion/exclusion of mice after combining data from 15 preclinical studies. A, Imaging data were collected from a total of 291 C57Bl/6 mice that underwent different occlusion times. The effect of occlusion time and surgeon on the heterogeneity of the stroke volume was assessed. Next, we continued with the 45-minute MCAO occlusion time, added another 129 transgenic mice, and built a prediction tool for the functional outcome. We additionally assessed the influence of the genotype and the surgeon on the heterogeneity of the stroke volumes. **B**, Multiple exclusion criteria were applied to a cohort of 450 mice undergoing 45 minutes of MCAO. After the exclusion process, behavioral data for a total of 185 MCAO and 30 sham mice were available. (Figure Created with BioRender.com)

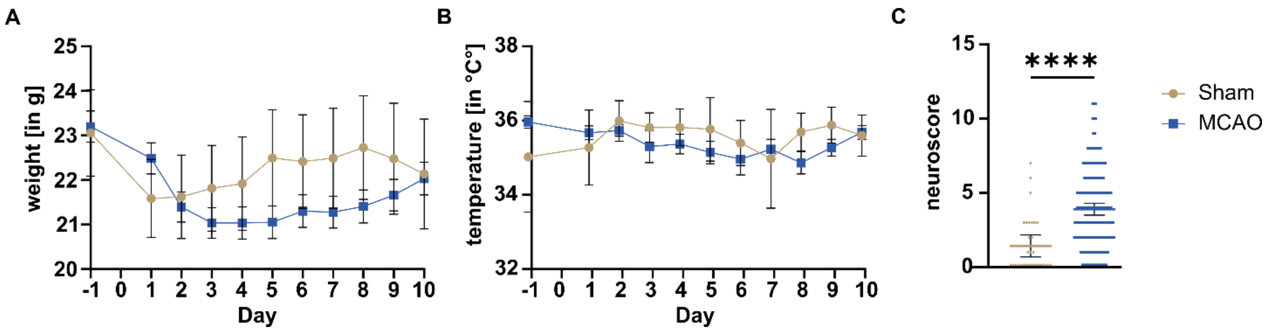

Figure S2. Weight and Temperature of MCAO and sham animals after surgery.
A, Weights and **B**, temperature of sham and MCAO animals within the first 10 days of surgery. Error bars indicate the 95% confidence interval. Days -1 to 8 (sham: n = 30; MCAO: n = 185) and days 9 to 10 (sham: n = 25-26; MCAO: n = 182-183). **C**, results of the modified DeSimoni neuroscore for the assessment of general deficits on day 2. Error bars indicate a 95% confidence interval (sham: n = 30; MCAO: n = 174).

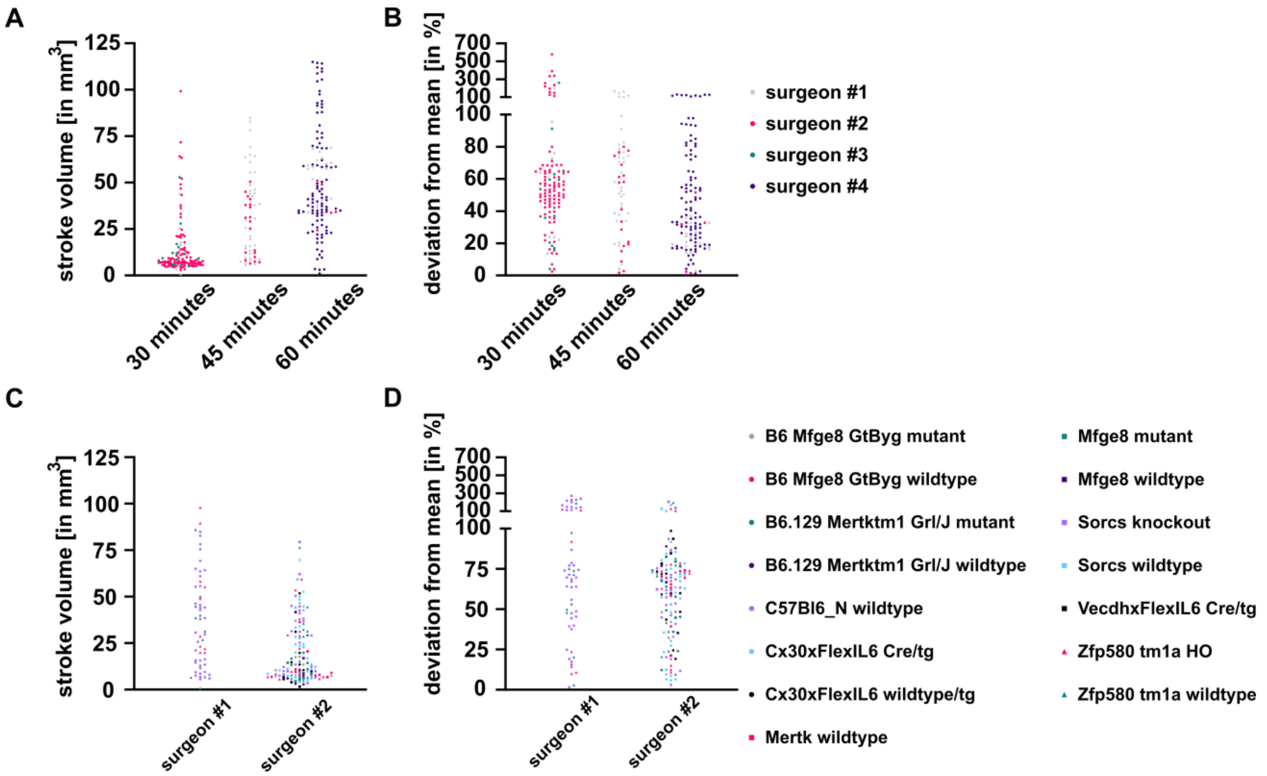

Figure S3 Stroke Volumes and Deviation from Mean by Surgeon and Occlusion Time. A and **B**, Linear regression model showed that the occlusion time, but not surgeon had a statistically significant effect on the stroke volume in the cohort of 291 C57Bl/6 mice. **C** and **D**, In the 45 minutes MCAO cohort, two surgeons and 15 different genotypes were included. Here, both factors showed a significant effect on the stroke volume.

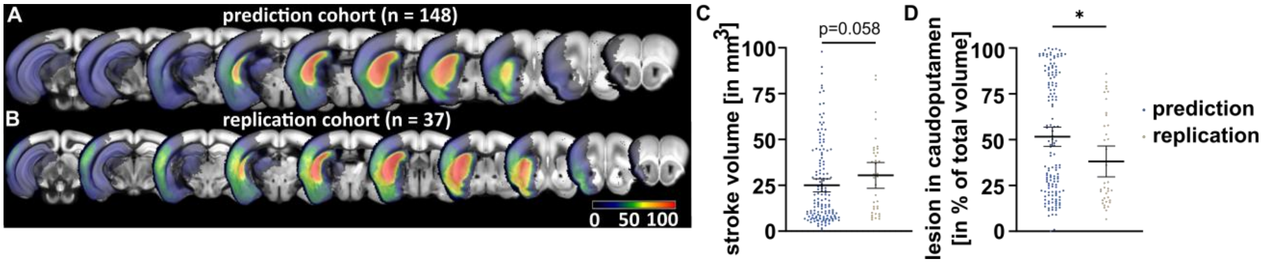

Figure S4 MRI characterization and lesion topology.
A, Lesion incidence maps of mice in the prediction and **B**, replication cohort. **C**, While stroke volumes were only trending towards being significantly larger in the replication cohort, **D** percentage of the total stroke volume that was within the caudoputamen was significantly smaller in the replication cohort. This indicates, that in the prediction cohort, cortical involvement was less frequent.

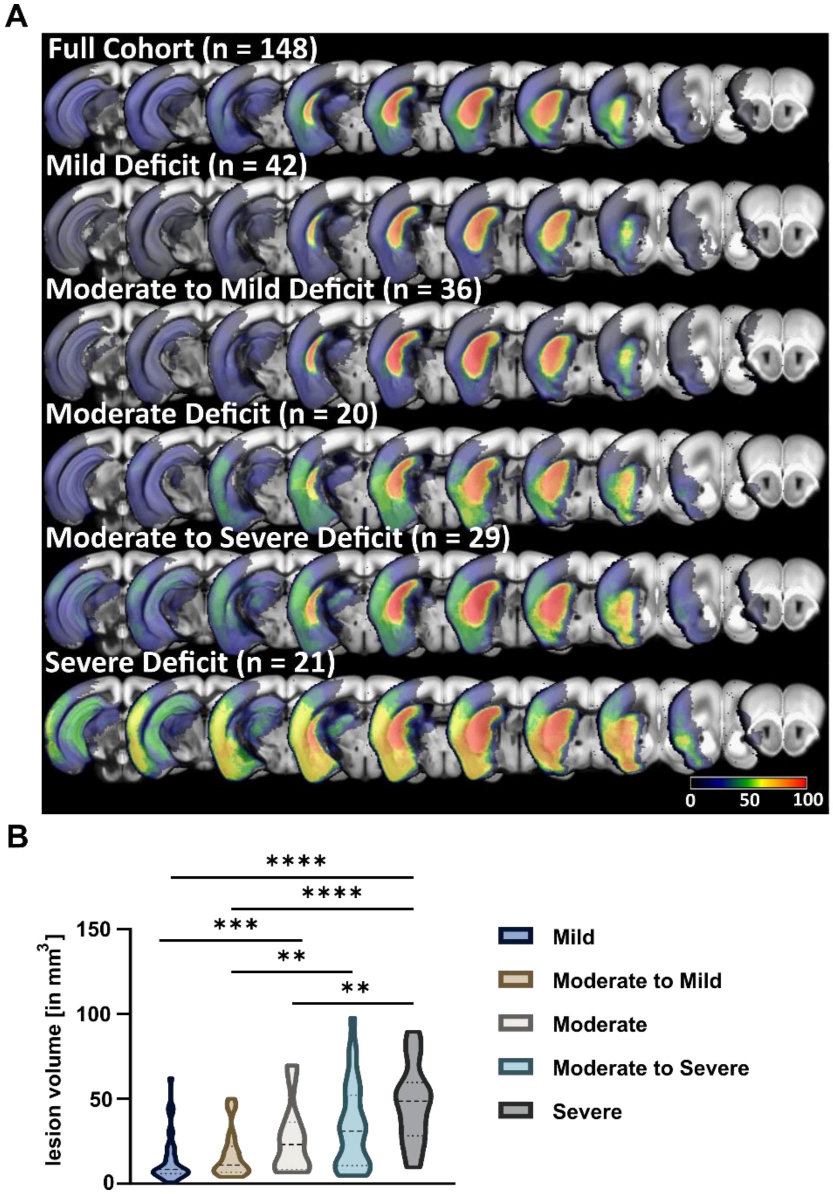

Figure S5. MRI characterization of lesion volume and location.

A, Coronal incidence maps of stroke lesions in the total cohort and the five different functional subgroups. Colours indicate percentage of mice with a lesion in a given region. Severity grades are based on degree of *residual deficit*. **B**, *lesion volume* by severity grade (based on degree of *residual deficit*). Great variability within functional subgroups could be observed and there was no significant difference when comparing *lesion volume* of consecutive severity grades, i.e. of mice with mild or moderate to mild *residual deficits*.

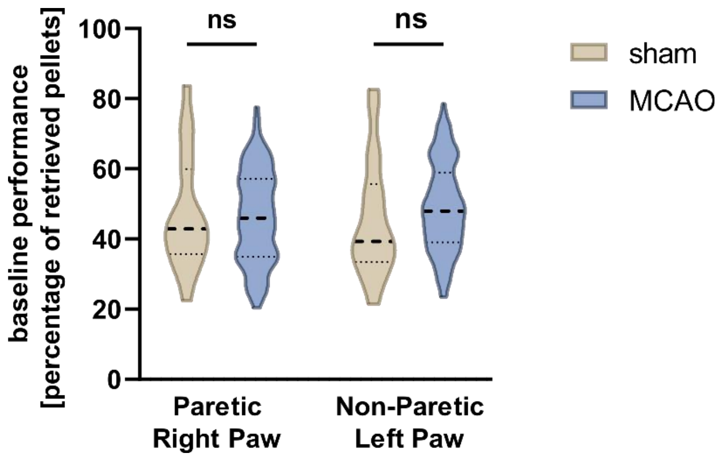

Figure S6 Comparison of baseline performance between sham and MCAO reveals no significant difference. Percentage of retrieved pellets during baseline testing (days -7 to -1). Mixed-effects model followed by Šidák’s post-hoc tests for multiple comparison were used to test effect of group and side. No statistically significant differences were found. Dashed lines indicate medians, dotted line indicate quartiles.

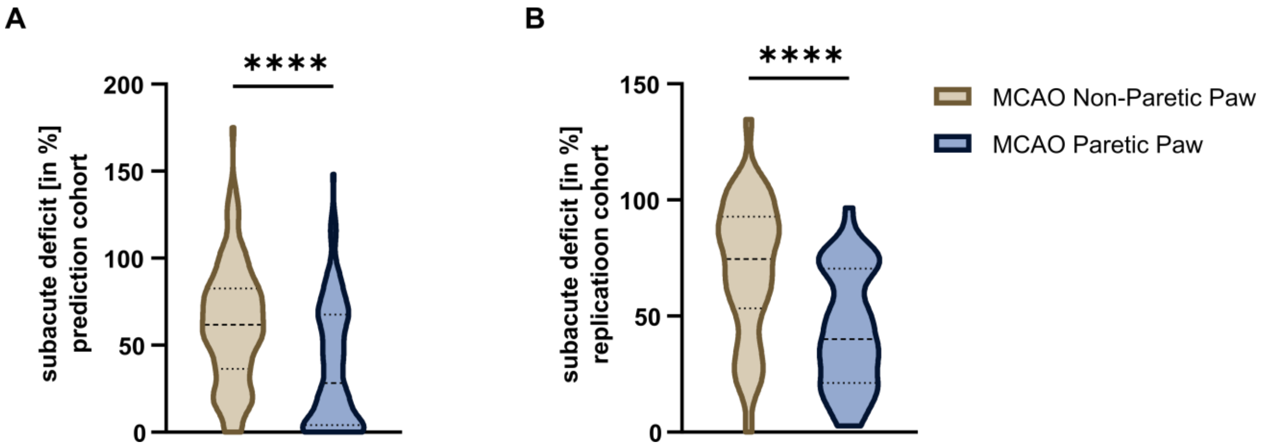

Figure S7 Comparison of the subacute deficit of non-paretic and paretic paw in the MCAO mice. A, Data on the subacute deficit are pooled from the training and testing cohorts. Paired t-test was performed to test for side-specific motor-functional deficits. Dashed lines indicate medians, dotted line indicate quartiles. **B**, Subacute deficit of the replication cohort. Again, a side specific decrease of the paretic paw was observed.

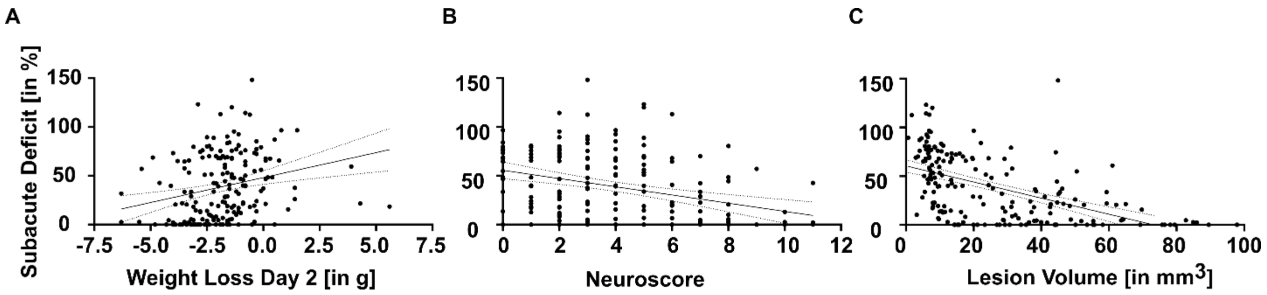

Figure S8. Assessment of the Effects of General Well-Being of Animals on Subacute Deficit
A, Simple linear regression analysis was performed for the weight loss on day 2 **B**, the neuroscore and **C**, the lesion volume to test whether these parameters correlate to the degree of the subacute deficit. Dotted line indicates 95% confidence band of the best-fit line.

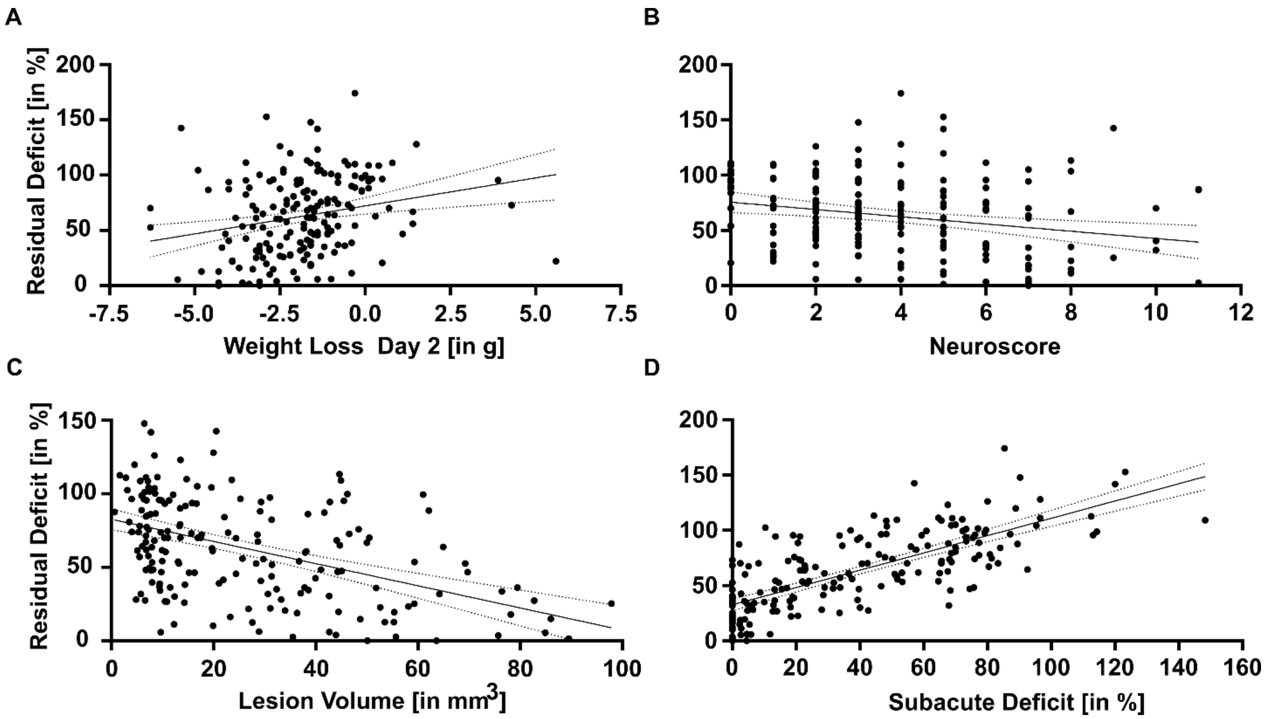

Figure S9. Assessment of the Effects of General Well-Being of Animals on Residual Deficit
A, Simple linear regression analysis was performed for the weight loss on day 2 **B**, the neuroscore **C**, the lesion volume and **D**, the subacute deficit to test whether these parameters correlate to the degree of the subacute deficit. Dotted line indicates 95% confidence band of the best-fit line.

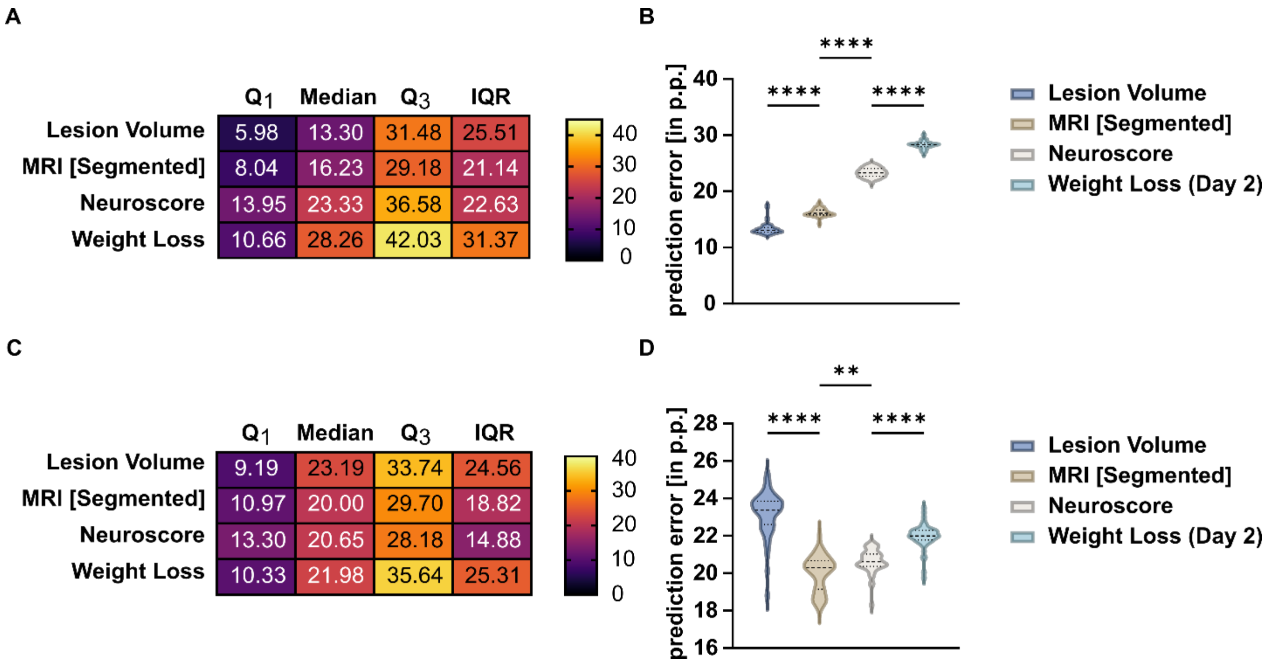

Figure S10. General well-being as predictor of subacute deficit

A, and **B**, *Prediction error* of the four models for the prediction of the *subacute deficit* in the testing cohort **C**, and **D** *Prediction error* of the four models for the prediction of the *subacute deficit* in the *replication cohort*.

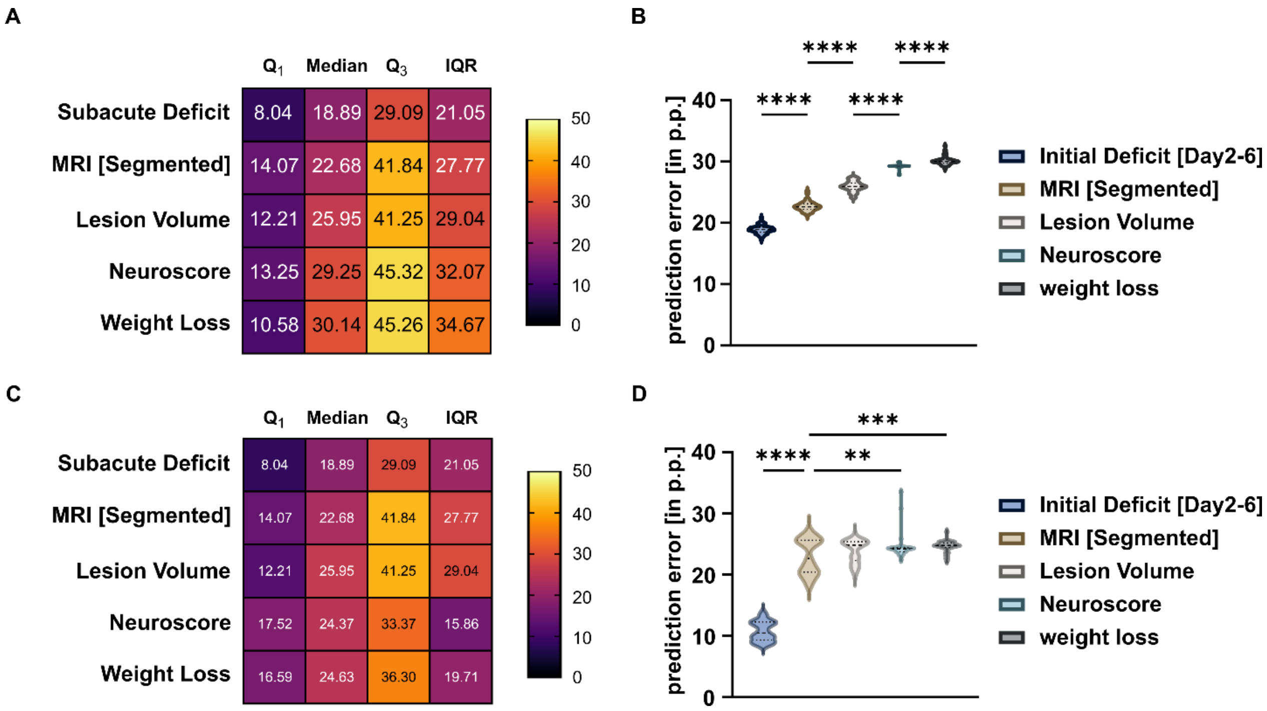

Figure S11. General well-being as predictor of residual deficit
A, and **B**, *Prediction error* of the four models for the prediction of the *residual deficit* in the testing cohort **C**, and **D** *Prediction error* of the four models for the prediction of the *residual deficit* in the *replication cohort*.

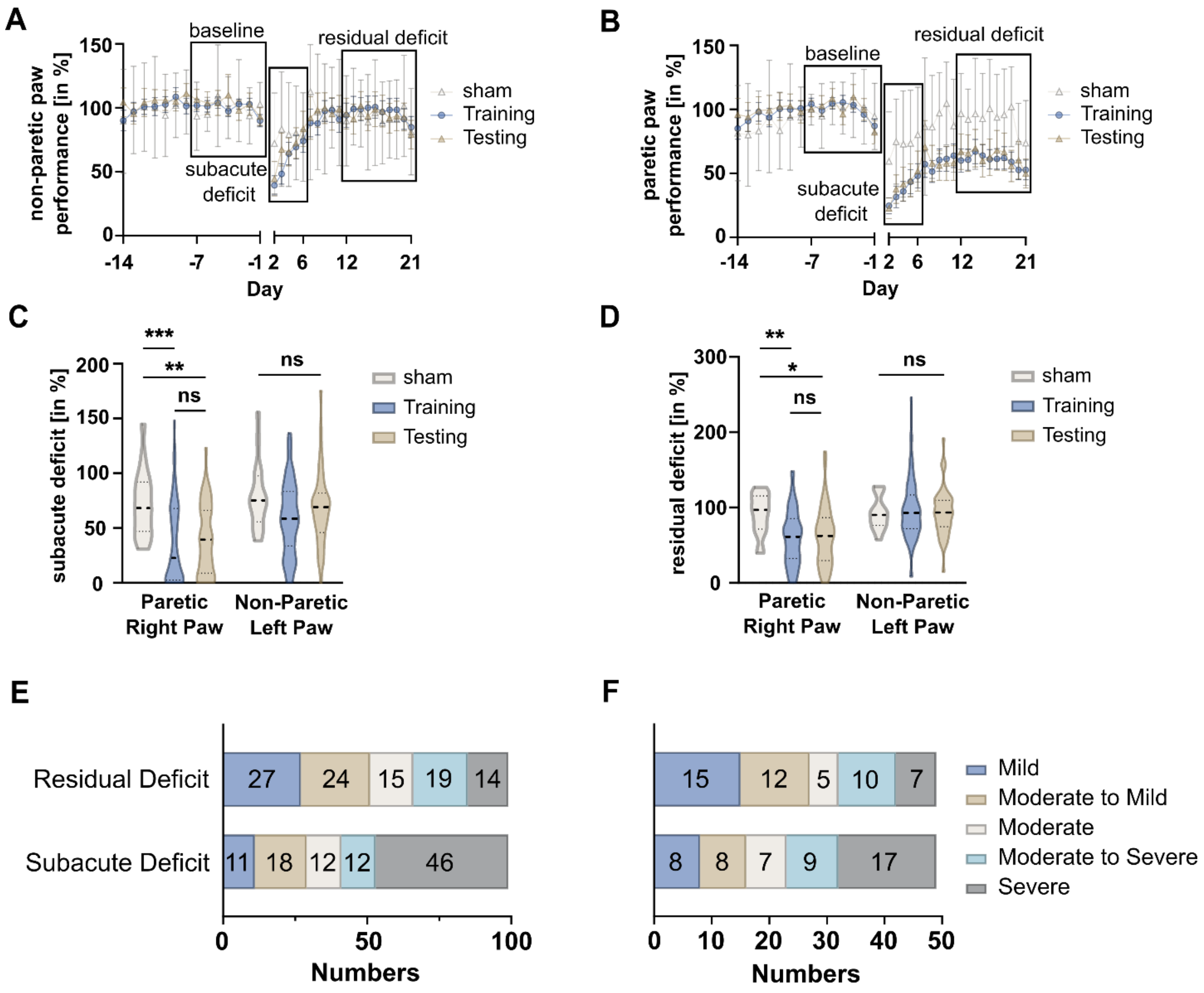

Figure S12. Analysis of the behavioral data of the training and testing cohort
A, Non-paretic left and **B**, paretic right paw performance over time, bars depict 95 % confidence interval. Performance on days -7 to -1 were averaged and taken as a baseline (first black rectangle). Data from days 2 to 6 were summarized and referred to as *subacute deficit* (second rectangle) while data from days 12 to 21 are referred to as *residual deficit* (third rectangle). **C**, *Subacute* and **D**, *residual deficit* of the non-paretic and paretic paw in sham (white plot), *training cohort* (blue plot) and *testing cohort* (brown plot). Dashed lines indicate medians, dotted line indicate quartiles. Mixed-effects model followed by Šidák's post-hoc tests for multiple comparison were used to test effect of group and side. **E**, Overview over sizes of functional subgroups in the *training cohort* and **F**, *testing cohort*. Groups are either based on the degree of *subacute* or *residual deficit*.

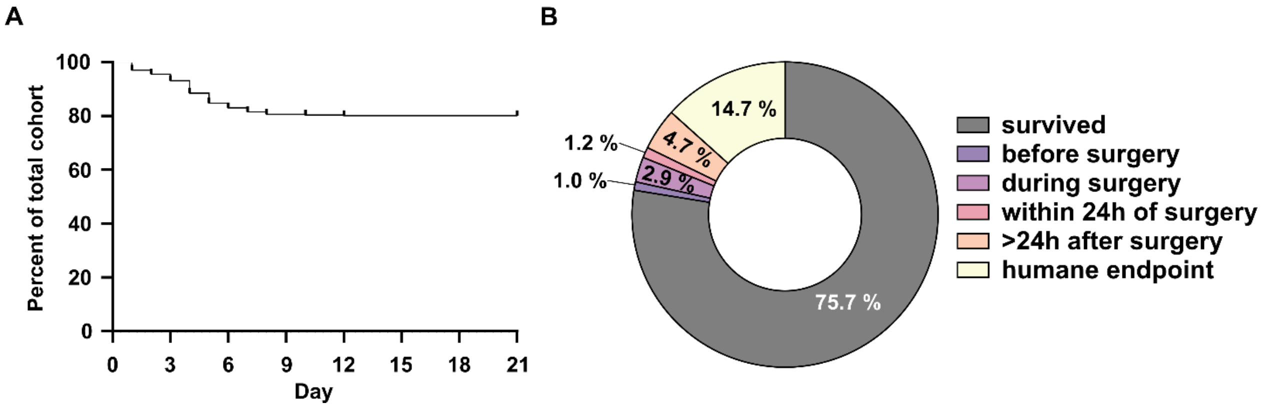

Figure S13. Overview of Mortality. A, Kaplan-Meier curve visualizing the survival of all animals undergoing sham or MCAO surgery. Most mice died within the first five days after the surgery. **B**, Mortality rates of the mice undergoing MCAO. Total mortality was 24.3%, which is in line with previous reports on longer MCA occlusion times. Surgery-associated death, e.g., rupture of vessels, was low, while most animals died or were euthanized after more than 24h after the surgery.

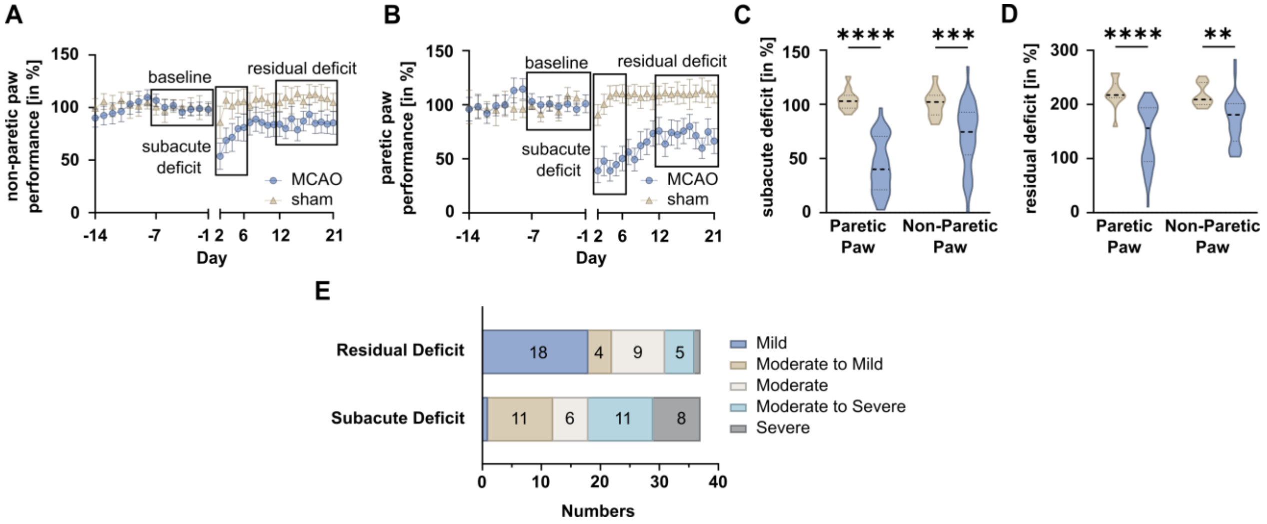

Figure S14. Assessment of motor-functional deficit and stroke composition

A, Non-paretic left and **B**, paretic right paw performance over time, bars depict 95 % confidence interval. Performance on days -7 to -1 were averaged and taken as a baseline (first black rectangle). Data from days 2 to 6 were summarized and referred to as *subacute deficit* (second rectangle) while data from days 12 to 21 are referred to as *residual deficit* (third rectangle). **C**, *Subacute* and **D**, *residual deficit* of the non-paretic and paretic paw in sham (brown plot) and MCAO (blue plot) animals. Dashed lines indicate medians, dotted line indicate quartiles. Mixed-effects model followed by Šidák's post-hoc tests for multiple comparison were used to test effect of group and side. **E**, Overview of sizes of functional subgroups. Groups are either based on the degree of *subacute* or *residual deficit*. In our replication cohort, only one mouse showed a mild *subacute* or severe *residual deficit* (not depicted). **F**, In the *replication cohort*, lesions in the caudoputamen accounted for a significantly smaller fraction when compared to the *testing cohort*.

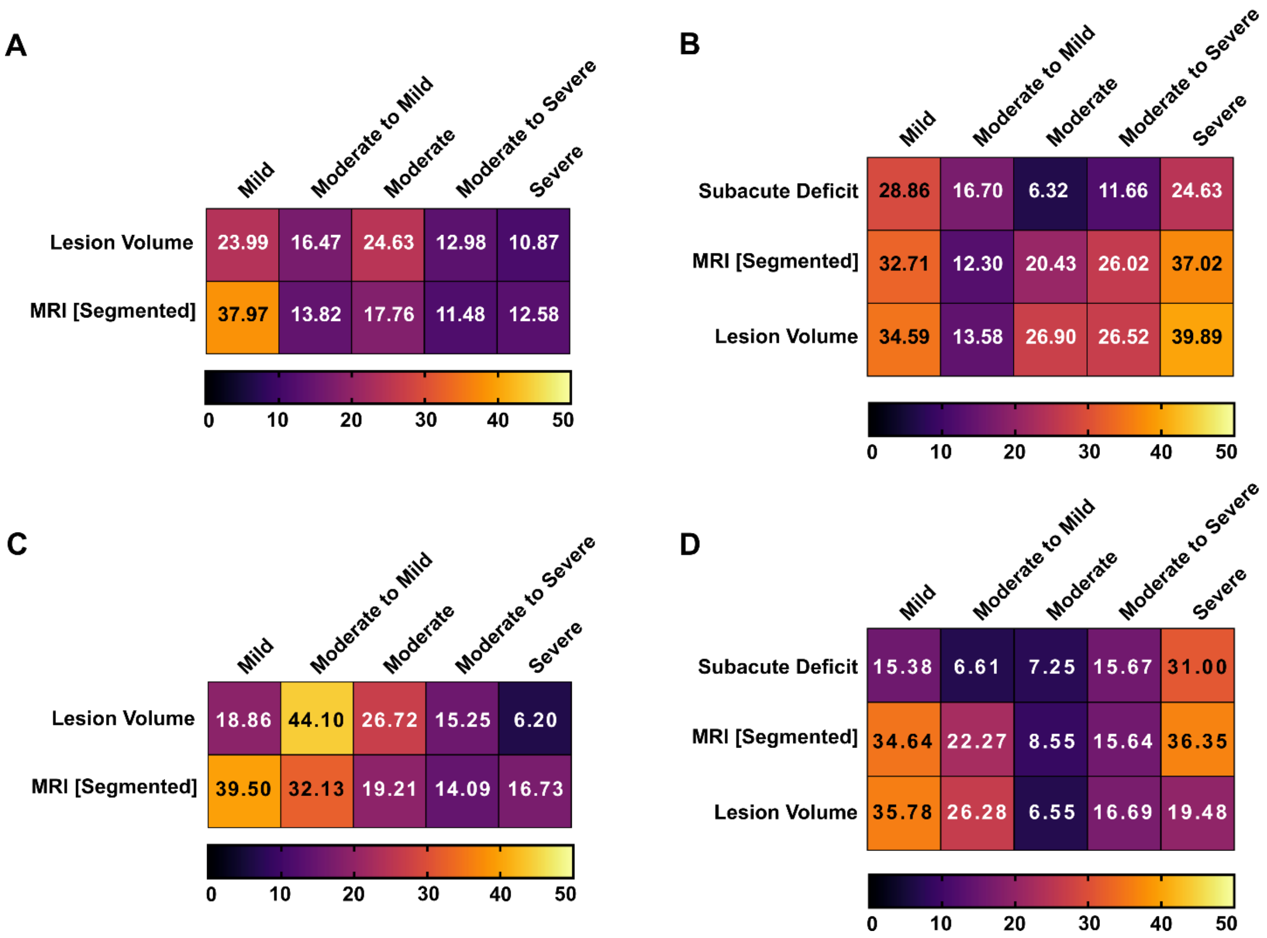

Figure S15 Comparison of severity grade dependent prediction accuracy.
A, *Prediction error* of the two predictors of the *subacute deficit* over the severity grades. Independent of the predictor, errors were higher in mice with mild compared to moderate to severe *subacute deficits*, indicating that the prediction models work especially well in those two crucial subgroups **B**, Prediction accuracy of the three predictors of the *residual deficit* over the severity grades. Again, a strong dependency of *prediction error* on the degree of deficit could be observed. **C**, *Prediction error* of the two predictors of the *subacute deficit* over the severity grades in the *replication cohort*. Again, *prediction errors* were smallest in mice with severe or moderate to severe subacute deficits. **D**, Prediction accuracy of the three predictors of the *residual deficit* over the severity grades in the *replication cohort*.

Experiment ID	sex	age at MCAO (in weeks)	Genotype	Genotype Activation	Therapeutic Treatment	MCAO vs Sham	cohort
2015-4	m	12	C57Bl6_N	none	none	MCAO	replication cohort
2015-4	m	12	C57Bl6_N	none	none	MCAO	replication cohort
2015-4	m	12	C57Bl6_N	none	none	MCAO	replication cohort
2015-4	m	12	C57Bl6_N	none	none	MCAO	replication cohort
2015-4	m	12	C57Bl6_N	none	none	MCAO	replication cohort
2015-4	m	12	C57Bl6_N	none	none	MCAO	replication cohort
2015-4	m	12	C57Bl6_N	none	none	MCAO	replication cohort
2015-4	m	12	C57Bl6_N	none	none	sham	replication cohort
2015-4	m	12	C57Bl6_N	none	none	sham	replication cohort
2015-4	m	12	C57Bl6_N	none	none	sham	replication cohort
2015-4	m	12	C57Bl6_N	none	none	sham	replication cohort
2015-4	m	12	C57Bl6_N	none	none	sham	replication cohort
2015-4	m	12	C57Bl6_N	none	none	sham	replication cohort
2015-4	m	12	C57Bl6_N	none	none	sham	replication cohort
2015-4	m	12	C57Bl6_N	none	none	sham	replication cohort
2015-4	m	12	C57Bl6_N	none	none	sham	replication cohort
2015-4	m	12	C57Bl6_N	none	none	sham	replication cohort
2015-4	m	12	C57Bl6_N	none	none	sham	replication cohort
2015-4	m	12	C57Bl6_N	none	none	sham	replication cohort
2015-8	m	13	C57Bl6_N	none	none	MCAO	replication cohort
2015-8	m	13	C57Bl6_N	none	none	MCAO	replication cohort
2015-8	m	13	C57Bl6_N	none	none	MCAO	replication cohort
2015-8	m	13	C57Bl6_N	none	none	MCAO	replication cohort

Continued Table S1

2015-8	m	13	C57Bl6_N	none	none	MCAO	replication cohort
2015-8	m	13	C57Bl6_N	none	none	MCAO	replication cohort
2015-8	m	13	C57Bl6_N	none	none	MCAO	replication cohort
2015-8	m	13	C57Bl6_N	none	none	MCAO	replication cohort
2015-8	m	13	C57Bl6_N	none	none	MCAO	replication cohort
2015-8	m	13	C57Bl6_N	none	none	MCAO	replication cohort
2015-8	m	13	C57Bl6_N	none	none	MCAO	replication cohort
2016-11	m	13	B6.129 Mertktm1 Grl/J mutant	none	none	MCAO	Training
2016-11	m	13	B6.129 Mertktm1 Grl/J wildtype	none	none	MCAO	Testing
2016-11	m	13	B6.129 Mertktm1 Grl/J mutant	none	none	MCAO	Training
2016-11	m	13	Mfge8 mutant	none	none	MCAO	Training
2016-11	m	14	Mfge8 mutant	none	none	MCAO	Training
2016-11	m	14	Mfge8 mutant	none	none	MCAO	Testing
2016-11	m	14	B6 Mfge8 GtByg wildtype	none	none	MCAO	Training
2016-11	m	14	B6.129 Mertktm1 Grl/J wildtype	none	none	MCAO	Training
2016-11	m	15	B6.129 Mertktm1 Grl/J wildtype	none	none	MCAO	Training
2016-11	m	15	B6 Mfge8 GtByg mutant	none	none	MCAO	Training
2016-11	m	16	Mfge8 mutant	none	none	MCAO	Training
2016-11	m	16	Mfge8 mutant	none	none	MCAO	Training
2016-11	m	17	B6 Mfge8 GtByg mutant	none	none	MCAO	Training
2016-11	m	17	B6 Mfge8 GtByg mutant	none	none	MCAO	Training
2016-11	m	17	B6 Mfge8 GtByg mutant	none	none	MCAO	Training
2016-11	m	17	B6.129 Mertktm1 Grl/J mutant	none	none	MCAO	Training

Continued Table S1

2016-11	m	18	B6 Mfge8 GtByg wildtype	none	none	MCAO	Training
2016-2	f	17	VecdhxFlexIL6 Cre/tg	Tamoxifen	none	sham	sham
2016-2	f	19	VecdhxFlexIL6 Cre/tg	Tamoxifen	none	MCAO	Training
2016-2	m	20	VecdhxFlexIL6 Cre/tg	Tamoxifen	none	MCAO	Testing
2016-2	m	21	VecdhxFlexIL6 Cre/tg	Tamoxifen	none	sham	sham
2016-2	f	21	VecdhxFlexIL6 Cre/tg	Tamoxifen	none	sham	sham
2016-2	f	23	VecdhxFlexIL6 Cre/tg	Tamoxifen	none	MCAO	Testing
2016-4	m	11	B6.129 Mertktm1 Grl/J mutant	none	none	sham	sham
2016-4	m	11	B6.129 Mertktm1 Grl/J mutant	none	none	MCAO	Training
2016-4	m	11	B6.129 Mertktm1 Grl/J mutant	none	none	MCAO	Testing
2016-4	m	11	B6.129 Mertktm1 Grl/J wildtype	none	none	sham	sham
2016-4	m	11	B6.129 Mertktm1 Grl/J wildtype	none	none	sham	sham
2016-9	m	15	Mertk wildtype	none	none	MCAO	Training
2016-9	m	16	B6 Mfge8 GtByg mutant	none	none	MCAO	Training
2016-9	m	16	B6.129 Mertktm1 Grl/J mutant	none	none	MCAO	Training
2016-9	m	16	B6.129 Mertktm1 Grl/J mutant	none	none	MCAO	Testing
2016-9	m	16	B6 Mfge8 GtByg wildtype	none	none	MCAO	Training
2016-9	m	16	B6 Mfge8 GtByg wildtype	none	none	MCAO	Training
2016-9	m	16	B6.129 Mertktm1 Grl/J mutant	none	none	MCAO	Testing

Continued Table S1

2016-9	m	16	B6.129 Mertktm1 Grl/J wildtype	none	none	MCAO	Training
2016-9	m	17	B6 Mfge8 GtByg wildtype	none	none	MCAO	Testing
2016-9	m	17	B6 Mfge8 GtByg mutant	none	none	MCAO	Training
2016-9	m	17	Mertk wildtype	none	none	MCAO	Training
2016-9	m	17	B6 Mfge8 GtByg wildtype	none	none	MCAO	Testing
2016-9	m	18	Mfge8 wildtype	none	none	MCAO	Testing
2016-9	m	19	B6 Mfge8 GtByg wildtype	none	none	MCAO	Training
2016-9	m	19	B6 Mfge8 GtByg wildtype	none	none	MCAO	Training
2016-9	m	20	B6.129 Mertktm1 Grl/J wildtype	none	none	MCAO	Training
2016-9	m	20	Mfge8 wildtype	none	none	MCAO	Training
2016-9	m	33	Mfge8 wildtype	none	none	MCAO	Training
2017-1	m	16	Cx30xFlexIL6 wildtype/tg	Tamoxifen	none	MCAO	Training
2017-1	m	16	Cx30xFlexIL6 wildtype/tg	Tamoxifen	none	sham	sham
2017-1	f	16	Cx30xFlexIL6 wildtype/tg	Tamoxifen	none	MCAO	Training
2017-1	m	16	Cx30xFlexIL6 wildtype/tg	Tamoxifen	none	MCAO	Training
2017-1	f	16	Cx30xFlexIL6 wildtype/tg	Tamoxifen	none	MCAO	Testing
2017-1	f	16	Cx30xFlexIL6 wildtype/tg	Tamoxifen	none	MCAO	Testing
2017-1	m	16	Cx30xFlexIL6 wildtype/tg	Tamoxifen	none	sham	sham
2017-1	m	17	Cx30xFlexIL6 Cre/tg	Tamoxifen	none	MCAO	Training
2017-1	f	17	Cx30xFlexIL6 Cre/tg	Tamoxifen	none	MCAO	Testing
2017-1	f	17	Cx30xFlexIL6 Cre/tg	Tamoxifen	none	MCAO	Testing
2017-1	f	17	Cx30xFlexIL6 Cre/tg	Tamoxifen	none	sham	sham

Continued Table S1

2017-1	f	17	Cx30xFlexIL6 Cre/tg	Tamoxifen	none	sham	sham
2017-1	f	18	Cx30xFlexIL6 Cre/tg	Tamoxifen	none	sham	sham
2017-1	m	18	Cx30xFlexIL6 Cre/tg	Tamoxifen	none	MCAO	Testing
2017-1	f	19	Cx30xFlexIL6 Cre/tg	Tamoxifen	none	sham	sham
2017-1	m	19	Cx30xFlexIL6 Cre/tg	Tamoxifen	none	MCAO	Testing
2017-1	m	21	Cx30xFlexIL6 wildtype/tg	Tamoxifen	none	MCAO	Training
2017-1	m	21	Cx30xFlexIL6 wildtype/tg	Tamoxifen	none	sham	sham
2017-1	f	22	Cx30xFlexIL6 wildtype/tg	Tamoxifen	none	MCAO	Testing
2017-1	m	24	Cx30xFlexIL6 cre/tg	Tamoxifen	none	sham	sham
2017-2	f	12	Cx30xFlexIL6 wildtype/tg	Tamoxifen	none	MCAO	Training
2017-2	f	12	Cx30xFlexIL6 cre/tg	Tamoxifen	none	MCAO	Training
2017-2	f	12	Cx30xFlexIL6 cre/tg	Tamoxifen	none	MCAO	Training
2017-2	m	13	Cx30xFlexIL6 cre/tg	Tamoxifen	none	MCAO	Training
2017-2	m	13	Cx30xFlexIL6 cre/tg	Tamoxifen	none	MCAO	Testing
2017-2	f	13	Cx30xFlexIL6 cre/tg	Tamoxifen	none	MCAO	Testing
2017-2	m	13	Cx30xFlexIL6 wildtype/tg	Tamoxifen	none	MCAO	Training
2017-2	f	13	Cx30xFlexIL6 wildtype/tg	Tamoxifen	none	MCAO	Training
2017-6	m	12	Zfp580 tm1a HO	none	none	sham	sham
2017-6	m	12	Zfp580 tm1a wildtype	none	none	MCAO	Testing
2017-6	m	12	Zfp580 tm1a wildtype	none	none	MCAO	Training
2017-6	m	12	Zfp580 tm1a wildtype	none	none	MCAO	Testing
2017-6	f	12	Zfp580 tm1a wildtype	none	none	MCAO	Training
2017-6	f	12	Zfp580 tm1a HO	none	none	MCAO	Training
2017-6	m	12	Zfp580 tm1a HO	none	none	MCAO	Training

Continued Table S1

2017-6	m	12	Zfp580 tm1a HO	none	none	MCAO	Training
2017-6	m	14	Zfp580 tm1a wildtype	none	none	MCAO	Testing
2017-6	m	15	Zfp580 tm1a HO	none	none	MCAO	Testing
2017-6	f	15	Zfp580 tm1a HO	none	none	MCAO	Testing
2017-6	m	17	Zfp580 tm1a HO	none	none	MCAO	Training
2017-6	m	17	Zfp580 tm1a wiltdype	none	none	MCAO	Testing
2017-6	f	17	Zfp580 tm1a wildtype	none	none	MCAO	Training
2017-6	f	17	Zfp580 tm1a HO	none	none	MCAO	Training
2017-6	f	17	Zfp580 tm1a wildtype	none	none	MCAO	Training
2017-6	m	17	Zfp580 tm1a HO	none	none	MCAO	Testing
2017-6	f	17	Zfp580 tm1a HO	none	none	MCAO	Testing
2017-6	f	19	Zfp580 tm1a wildtype	none	none	MCAO	Training
2017-6	f	19	Zfp580 tm1a HO	none	none	MCAO	Training
2017-6	f	19	Zfp580 tm1a HO	none	none	MCAO	Training
2017-6	f	19	Zfp580 tm1a wildtype	none	none	MCAO	Testing
2017-6	f	19	Zfp580 tm1a HO	none	none	MCAO	Training
2017-6	f	21	Zfp580 tm1a wildtype	none	none	MCAO	Training
2017-6	f	21	Zfp580 tm1a wildtype	none	none	sham	sham
2017-8	m	14	Cx30xFlexIL6 cre/tg	Tamoxifen	none	MCAO	Training
2017-8	m	14	Cx30xFlexIL6 cre/tg	Tamoxifen	none	MCAO	Training
2017-8	m	14	Cx30xFlexIL6 cre/tg	Tamoxifen	none	MCAO	Testing
2017-8	m	14	Cx30xFlexIL6 cre/tg	Vehikel	none	MCAO	Training
2017-8	m	14	Cx30xFlexIL6 cre/tg	Vehikel	none	MCAO	Training
2017-8	f	14	Cx30xFlexIL6 cre/tg	Vehikel	none	MCAO	Training

Continued Table S1

2017-8	m	15	Cx30xFlexIL6 cre/tg	Vehikel	none	MCAO	Training
2017-8	f	15	Cx30xFlexIL6 cre/tg	Tamoxifen	none	MCAO	Training
2017-8	m	15	Cx30xFlexIL6 cre/tg	Vehikel	none	MCAO	Training
2017-8	f	15	Cx30xFlexIL6 cre/tg	Tamoxifen	none	MCAO	Training
2017-8	m	15	Cx30xFlexIL6 cre/tg	Tamoxifen	none	MCAO	Training
2017-8	m	16	Cx30xFlexIL6 cre/tg	Tamoxifen	none	MCAO	Testing
2017-8	f	16	Cx30xFlexIL6 cre/tg	Tamoxifen	none	MCAO	Testing
2017-8	f	16	Cx30xFlexIL6 cre/tg	Vehikel	none	MCAO	Testing
2017-8	m	16	Cx30xFlexIL6 cre/tg	Tamoxifen	none	MCAO	Training
2017-8	f	16	Cx30xFlexIL6 cre/tg	Vehikel	none	MCAO	Training
2018-4	f	13	Zfp580 tm1a HO	none	none	MCAO	Testing
2018-4	m	13	Zfp580 tm1a HO	none	none	MCAO	Training
2018-4	f	14	Zfp580 tm1a wildtype	none	none	MCAO	Training
2018-4	m	14	Zfp580 tm1a wildtype	none	none	MCAO	Testing
2018-4	m	14	Zfp580 tm1a HO	none	none	MCAO	Training
2018-4	m	14	Zfp580 tm1a wildtype	none	none	MCAO	Training
2018-4	m	16	Zfp580 tm1a HO	none	none	MCAO	Testing
2018-4	f	17	Zfp580 tm1a HO	none	none	MCAO	Training
2018-4	m	17	Zfp580 tm1a HO	none	none	MCAO	Training
2018-4	m	18	Zfp580 tm1a HO	none	none	MCAO	Testing
2018-4	m	18	Zfp580 tm1a HO	none	none	MCAO	Training
2018-4	f	18	Zfp580 tm1a HO	none	none	MCAO	Training
2018-4	f	18	Zfp580 tm1a wildtype	none	none	MCAO	Training
2018-4	f	19	Zfp580 tm1a wildtype	none	none	MCAO	Training

Continued Table S1

2018-4	m	20	Zfp580 tm1a wildtype	none	none	MCAO	Training
2018-4	m	20	Zfp580 tm1a wildtype	none	none	MCAO	Training
2018-4	m	20	Zfp580 tm1a wildtype	none	none	MCAO	Training
2018-4	m	20	Zfp580 tm1a wildtype	none	none	MCAO	Training
2018-4	m	20	Zfp580 tm1a wildtype	none	none	MCAO	Training
2018-4	f	20	Zfp580 tm1a HO	none	none	MCAO	Training
2018-4	f	20	Zfp580 tm1a wildtype	none	none	sham	sham
2018-8	m	13	Sorcs wildtype	none	none	MCAO	Testing
2018-8	m	14	Sorcs knockout	none	none	MCAO	Testing
2018-8	m	14	Sorcs wildtype	none	none	sham	sham
2018-8	m	14	Sorcs knockout	none	none	MCAO	Training
2018-8	m	14	Sorcs knockout	none	none	MCAO	Training
2018-8	m	14	Sorcs wildtype	none	none	MCAO	Testing
2018-8	m	14	Sorcs knockout	none	none	MCAO	Training
2018-8	m	14	Sorcs wildtype	none	none	MCAO	Testing
2018-8	m	14	Sorcs knockout	none	none	MCAO	Testing
2018-8	m	17	Sorcs wildtype	none	none	MCAO	Testing
2018-8	m	17	Sorcs wildtype	none	none	MCAO	Training
2019-1	m	15	C57Bl6_N wildtype	none	Kontroll-Peptid	MCAO	Testing
2019-1	m	15	C57Bl6_N wildtype	none	Cycl (RADM)	MCAO	Training
2019-1	m	15	C57Bl6_N wildtype	none	Cycl (RADM)	MCAO	Testing
2019-1	m	15	C57Bl6_N wildtype	none	Kontroll-Peptid	MCAO	Testing
2019-1	m	15	C57Bl6_N wildtype	none	PBS	MCAO	Testing
2019-1	m	15	C57Bl6_N wildtype	none	PBS	MCAO	Training
2019-1	m	15	C57Bl6_N wildtype	none	Cycl (RADM)	MCAO	Training
2019-1	m	15	C57Bl6_N wildtype	none	Cilengitide	MCAO	Training
2019-1	m	15	C57Bl6_N wildtype	none	Cycl (RADM)	MCAO	Testing
2019-1	m	15	C57Bl6_N wildtype	none	Kontroll-Peptid	MCAO	Testing
2019-1	m	15	C57Bl6_N wildtype	none	Cilengitide	MCAO	Training

Continued Table S1

2019-1	m	15	C57Bl6_N wildtype	none	PBS	MCAO	Training
2019-1	m	15	C57Bl6_N wildtype	none	Cilengitide	MCAO	Training
2019-1	m	15	C57Bl6_N wildtype	none	Cycl (RADM)	MCAO	Training
2019-1	m	15	C57Bl6_N wildtype	none	Cycl (RADM)	MCAO	Training
2019-1	m	15	C57Bl6_N wildtype	none	PBS	MCAO	Training
2019-1	m	15	C57Bl6_N wildtype	none	Kontroll-Peptid	MCAO	Training
2019-1	m	15	C57Bl6_N wildtype	none	Cilengitide	MCAO	Testing
2019-1	m	15	C57Bl6_N wildtype	none	Kontroll-Peptid	MCAO	Training
2019-8	f	14	C57Bl6_N	none	metamizole	MCAO	replication cohort
2019-8	m	14	C57Bl6_N	none	none	MCAO	replication cohort
2019-8	m	14	C57Bl6_N	none	none	MCAO	replication cohort
2019-8	m	14	C57Bl6_N	none	metamizole	MCAO	replication cohort
2019-8	f	14	C57Bl6_N	none	metamizole	MCAO	replication cohort
2019-8	f	14	C57Bl6_N	none	none	MCAO	replication cohort
2019-8	f	14	C57Bl6_N	none	none	MCAO	replication cohort
2019-8	m	15	C57Bl6_N	none	none	MCAO	replication cohort
2019-8	m	15	C57Bl6_N	none	none	MCAO	replication cohort
2019-8	m	15	C57Bl6_N	none	none	MCAO	replication cohort
2019-8	f	15	C57Bl6_N	none	metamizole	MCAO	replication cohort
2019-8	f	15	C57Bl6_N	none	none	MCAO	replication cohort
2019-8	f	15	C57Bl6_N	none	none	MCAO	replication cohort
2019-8	m	15	C57Bl6_N	none	metamizole	MCAO	replication cohort
2019-8	m	15	C57Bl6_N	none	metamizole	MCAO	replication cohort
2019-8	m	15	C57Bl6_N	none	none	MCAO	replication cohort

Continued Table S1

2019-8	f	15	C57Bl6_N	none	metamizole	MCAO	replication cohort
2019-8	f	15	C57Bl6_N	none	none	MCAO	replication cohort
2019-8	f	15	C57Bl6_N	none	none	MCAO	replication cohort

Table S1 Overview over the genotypes, treatments and sex of the mice included in the study.

	Total	Female	Male
**MCAO**	185	30	135
Baseline age in weeks †	15.39 (15.00-15.78)	16.07 (15.34-16.79)	15.14 (14.68-15.60)
Baseline weight in grams	23.20 (22.85-23.55)	21.33 (20.81-21.85)	23.89 (23.51-24.27)
weight Day 1	22.48 (22.14-22.83)	20.63 (20.07-21.19)	23.17 (22.80-23.54)
weight Day 2	21.39 (21.05-21.73)	20.05 (19.42-20.68)	21.89 (21.52-22.26)
Neuroscore Day 2 *	3.90 (3.51-4.30)	2.84 (2.22-3.46)	4.33 (3.85-4.81)

Table S2: Overview of the basic characteristics of animals. † denotes age in weeks at the time of the surgery. * denotes the modified DeSimoni neuroscore for the assessment of general deficits. The neuroscore was available for a total of 174 MCAO mice, of which 124 were male and 50 were female.

Label	Lesion percentage	SEM
Caudoputamen	55.75	1.37
Claustrum	40.41	2.61
Agranular insular area, dorsal part, layer 6b	44.63	2.95
Fundus of striatum	44.15	2.99
Central amygdalar nucleus, lateral part	39.92	2.92
Visceral area, layer 6b	47.56	3.59
Globus pallidus, external segment	18.58	1.40
Agranular insular area, posterior part, layer 6a	48.21	3.71
Endopiriform nucleus, dorsal part	35.03	2.73
Olfactory tubercle, polymorph layer	25.42	2.01
Gustatory areas, layer 6b	34.47	2.75
Nucleus accumbens	14.22	1.14
Agranular insular area, ventral part, layer 6a	20.15	1.68
Substantia innominata	14.09	1.21
Basic cell groups and regions	7.77	0.67
Piriform area, polymorph layer	35.15	3.13
Agranular insular area, posterior part, layer 5	42.03	3.77
Agranular insular area, dorsal part, layer 6a	25.15	2.26
Piriform area, pyramidal layer	31.49	2.87
Supplemental somatosensory area, layer 6b	33.98	3.10
Lateral amygdalar nucleus	34.65	3.18
Central amygdalar nucleus, capsular part	35.22	3.30
Visceral area, layer 6a	39.98	3.75
Cerebral cortex	26.47	2.48
Central amygdalar nucleus, medial part	31.68	2.97
Islands of Calleja	12.27	1.17

Continued Table S3

Olfactory tubercle, pyramidal layer	15.37	1.47
Gustatory areas, layer 6a	29.37	2.82
Endopiriform nucleus, ventral part	34.82	3.38
Piriform area, molecular layer	27.52	2.67
Anterior amygdalar area	29.33	2.90
Agranular insular area, posterior part, layer 2/3	37.24	3.68
Striatum-like amygdalar nuclei	24.45	2.43
Agranular insular area, ventral part, layer 5	22.79	2.36
Olfactory areas	8.37	0.87
Supplemental somatosensory area, layer 6a	32.96	3.45
Intercalated amygdalar nucleus	32.68	3.42
Cortical amygdalar area, anterior part, layer 2	33.42	3.52
Visceral area, layer 5	35.08	3.69
Basomedial amygdalar nucleus, anterior part	34.00	3.62
Basolateral amygdalar nucleus, anterior part	32.45	3.47
Entorhinal area, lateral part, layer 4/5	33.22	3.55
Agranular insular area, posterior part, layer 1	31.56	3.39
Cortical amygdalar area, anterior part, layer 1	27.38	2.94
Supplemental somatosensory area, layer 5	32.09	3.59
Visceral area, layer 4	32.22	3.62
Nucleus of the lateral olfactory tract, pyramidal layer	30.72	3.47
Nucleus of the lateral olfactory tract, layer 3	31.28	3.54
Perirhinal area, layer 6a	24.33	2.77
Ventral auditory area, layer 6a	23.92	2.72
Visceral area, layer 2/3	31.17	3.56
Entorhinal area, lateral part, layer 2/3	28.74	3.28
Supplemental somatosensory area, layer 4	30.88	3.58

Continued Table S3

Supplemental somatosensory area, layer 2/3	29.81	3.47
Agranular insular area, ventral part, layer 2/3	23.77	2.77
Perirhinal area, layer 5	25.73	3.02
Ventral auditory area, layer 6b	19.09	2.26
Agranular insular area, dorsal part, layer 5	15.24	1.81
Gustatory areas, layer 5	25.25	3.01
Visceral area, layer 1	25.69	3.07
Piriform-amygdalar area, polymorph layer	29.81	3.57
Ventral auditory area, layer 5	25.86	3.11
Nucleus of the lateral olfactory tract, molecular layer	22.69	2.75
Olfactory tubercle, molecular layer	7.92	0.96
Perirhinal area, layer 2/3	25.04	3.04
Piriform-amygdalar area, pyramidal layer	28.17	3.43
Globus pallidus, internal segment	11.82	1.44
Magnocellular nucleus	10.24	1.25
Ventral auditory area, layer 4	26.55	3.26
Basomedial amygdalar nucleus, posterior part	27.55	3.38
Medial amygdalar nucleus, anterodorsal part	23.41	2.88
Basolateral amygdalar nucleus, ventral part	28.94	3.58
Piriform-amygdalar area, molecular layer	23.62	2.94
Cortical amygdalar area, posterior part, lateral zone, layer 2	25.40	3.21
Gustatory areas, layer 4	24.85	3.15
Agranular insular area, ventral part, layer 1	21.64	2.74
Ventral auditory area, layer 2/3	25.78	3.27
Entorhinal area, lateral part, layer 2	27.27	3.47
Primary somatosensory area, nose, layer 6b	22.08	2.81
Bed nucleus of the accessory olfactory tract	25.16	3.24

Continued Table S3

Perirhinal area, layer 1	18.09	2.34
Gustatory areas, layer 2/3	23.34	3.06
Perirhinal area, layer 6b	20.53	2.70
Medial amygdalar nucleus, anteroventral part	21.33	2.82
Temporal association areas, layer 6a	18.86	2.51
Medial amygdalar nucleus, posterodorsal part, sublayer b	22.60	3.01
Cortical amygdalar area, posterior part, lateral zone, layer 3	24.20	3.25
Gustatory areas, layer 1	20.62	2.77
Temporal association areas, layer 4	20.12	2.70
Temporal association areas, layer 5	20.15	2.71
Basolateral amygdalar nucleus, posterior part	23.52	3.17
Primary auditory area, layer 5	22.41	3.03
Supplemental somatosensory area, layer 1	17.86	2.42
Medial amygdalar nucleus, posterodorsal part, sublayer c	20.15	2.73
Primary auditory area, layer 2/3	23.53	3.20
Primary auditory area, layer 4	22.87	3.11
Temporal association areas, layer 2/3	19.22	2.61
Ectorhinal area/Layer 6a	17.78	2.43
Dorsal auditory area, layer 2/3	22.60	3.10
Dorsal auditory area, layer 4	22.77	3.13
Medial amygdalar nucleus, posterodorsal part, sublayer a	20.40	2.80
Primary somatosensory area, barrel field, layer 2/3	20.35	2.81
Primary somatosensory area, barrel field, layer 4	20.57	2.84
Primary somatosensory area, nose, layer 6a	23.64	3.27
Ectorhinal area/Layer 5	18.21	2.52
Dorsal auditory area, layer 5	21.18	2.94
Primary somatosensory area, nose, layer 5	24.30	3.39

Continued Table S3

Entorhinal area, lateral part, layer 6b	20.32	2.84
Cortical amygdalar area, posterior part, lateral zone, layer 1	18.02	2.53
Field CA3, stratum lacunosum-moleculare	23.62	3.32
Primary somatosensory area, nose, layer 2/3	22.58	3.17
Primary somatosensory area, nose, layer 4	23.85	3.35
Primary somatosensory area, barrel field, layer 5	18.79	2.65
Primary somatosensory area, barrel field, layer 6a	15.17	2.14
Temporal association areas, layer 1	13.81	1.95
Ventral auditory area, layer 1	18.92	2.67
Ventral posterolateral nucleus of the thalamus	13.02	1.85
Field CA2, stratum oriens	19.72	2.81
Field CA2, pyramidal layer	20.59	2.94
Ectorhinal area/Layer 2/3	16.48	2.36
Ectorhinal area/Layer 1	12.11	1.74
Dorsal auditory area, layer 1	18.15	2.61
Field CA3, pyramidal layer	18.47	2.66
Field CA2, stratum lacunosum-moleculare	20.87	3.00
Ectorhinal area/Layer 6b	17.53	2.52
Field CA2, stratum radiatum	19.88	2.86
Primary auditory area, layer 1	17.96	2.59
Field CA3, stratum oriens	15.61	2.26
Reticular nucleus of the thalamus	10.16	1.47
Primary auditory area, layer 6a	15.65	2.27
Field CA3, stratum lucidum	19.39	2.82
Field CA3, stratum radiatum	20.52	2.99
Agranular insular area, dorsal part, layer 2/3	12.03	1.75
Lateral dorsal nucleus of thalamus	18.67	2.72
Continued Table S3		

Postpiriform transition area, layers 1	18.92	2.80
Field CA1, stratum oriens	15.67	2.32
Agranular insular area, dorsal part, layer 1	10.44	1.55
Postpiriform transition area, layers 2	19.15	2.85
Postpiriform transition area, layers 3	19.92	2.98
Ventral posteromedial nucleus of the thalamus	12.40	1.85
Field CA1, stratum radiatum	18.52	2.78
Primary somatosensory area, barrel field, layer 6b	9.59	1.44
Dentate gyrus, polymorph layer	18.43	2.78
Cortical amygdalar area, posterior part, medial zone, layer 3	17.45	2.66
Dentate gyrus, granule cell layer	15.11	2.32
Thalamus	4.84	0.74
Medial amygdalar nucleus, posteroventral part	17.39	2.67
Entorhinal area, lateral part, layer 1	11.68	1.80
Field CA1, stratum lacunosum-moleculare	17.52	2.72
Field CA1, pyramidal layer	17.55	2.73
Primary somatosensory area, mouth, layer 4	18.17	2.83
Primary somatosensory area, barrel field, layer 1	14.24	2.22
Posterior complex of the thalamus	10.46	1.64
Primary somatosensory area, mouth, layer 2/3	16.88	2.65
Temporal association areas, layer 6b	15.05	2.38
Entorhinal area, lateral part, layer 5	16.12	2.56
Dentate gyrus, molecular layer	12.53	2.00
Primary somatosensory area, mouth, layer 5	16.52	2.65
Primary somatosensory area, unassigned, layer 6b	5.98	0.96
Entorhinal area, lateral part, layer 3	15.37	2.48
Entorhinal area, lateral part, layer 4	16.22	2.62
Continued Table S3		

Dorsal auditory area, layer 6a	12.60	2.04
Posterior amygdalar nucleus	15.80	2.56
Diagonal band nucleus	1.06	0.17
Posterior parietal association areas, layer 4	7.37	1.20
Entorhinal area, lateral part, layer 2a	13.60	2.24
Cortical amygdalar area, posterior part, medial zone, layer 2	13.12	2.16
Posterior parietal association areas, layer 5	6.06	1.00
Posterior parietal association areas, layer 2/3	7.17	1.18
Primary somatosensory area, mouth, layer 6a	14.34	2.38
Primary somatosensory area, nose, layer 1	13.35	2.23
Primary somatosensory area, unassigned, layer 4	13.71	2.29
Entorhinal area, lateral part, layer 2b	14.28	2.41
Primary somatosensory area, unassigned, layer 2/3	14.05	2.38
Entorhinal area, lateral part, layer 6a	14.06	2.42
Primary somatosensory area, trunk, layer 6b	3.60	0.62
Anteroventral nucleus of thalamus	11.59	2.01
Anterolateral visual area, layer 2/3	14.50	2.52
Posterior parietal association areas, layer 6b	4.94	0.86
Posterior parietal association areas, layer 1	5.05	0.88
Primary somatosensory area, unassigned, layer 5	12.10	2.14
Anterolateral visual area, layer 1	10.64	1.89
Posterior auditory area, layer 2/3	16.26	2.89
Anterolateral visual area, layer 4	13.96	2.49
Posterior auditory area, layer 4	16.59	2.98
Primary somatosensory area, unassigned, layer 1	9.93	1.81
Cortical amygdalar area, posterior part, medial zone, layer 1	8.91	1.63

Continued Table S3

Ventral anterior-lateral complex of the thalamus	3.48	0.64
Central lateral nucleus of the thalamus	10.29	1.90
Primary somatosensory area, mouth, layer 6b	9.68	1.80
Primary somatosensory area, mouth, layer 1	9.46	1.77
Posterior auditory area, layer 1	12.68	2.38
Dorsal auditory area, layer 6b	8.55	1.61
Primary auditory area, layer 6b	9.83	1.85
Posterior auditory area, layer 5	15.08	2.85
Anterodorsal nucleus	9.10	1.72
Lateral visual area, layer 2/3	13.27	2.53
Lateral visual area, layer 4	14.46	2.76
Anterolateral visual area, layer 5	12.11	2.32
Primary somatosensory area, upper limb, layer 4	10.49	2.03
Primary somatosensory area, unassigned, layer 6a	7.76	1.50
Subiculum, dorsal part, stratum radiatum	8.57	1.67
Lateral visual area, layer 5	13.81	2.69
Subiculum, ventral part, stratum radiatum	12.26	2.39
Subiculum, ventral part, molecular layer	12.56	2.45
Primary somatosensory area, upper limb, layer 2/3	10.29	2.03
Lateral hypothalamic area	0.48	0.09
Posterior parietal association areas, layer 6a	3.24	0.65
Primary somatosensory area, upper limb, layer 5	9.10	1.83
Hypothalamus	0.20	0.04
Orbital area, lateral part, layer 5	2.17	0.44
Subiculum, ventral part, pyramidal layer	10.88	2.21
Dorsal part of the lateral geniculate complex	8.80	1.82
Subiculum, dorsal part, molecular layer	8.07	1.68

Continued Table S3

Lateral visual area, layer 1	6.25	1.32
Posterolateral visual area, layer 4	11.78	2.49
Lateral posterior nucleus of the thalamus	8.41	1.78
Primary visual area, layer 2/3	7.00	1.48
Subiculum, dorsal part, pyramidal layer	6.45	1.37
Primary visual area, layer 4	7.74	1.66
Medial geniculate complex, ventral part	6.47	1.40
Posterior auditory area, layer 6a	12.64	2.73
Posterolateral visual area, layer 5	11.62	2.51
Presubiculum, layer 1	7.53	1.63
Lateral visual area, layer 6a	10.91	2.36
Orbital area, lateral part, layer 2/3	2.30	0.50
Posterior auditory area, layer 6b	12.52	2.73
Primary visual area, layer 5	7.45	1.63
Mediodorsal nucleus of the thalamus, lateral part	4.70	1.03
Supraoptic nucleus	1.54	0.34
Posterolateral visual area, layer 2/3	9.52	2.11
Primary somatosensory area, upper limb, layer 6a	6.64	1.50
Presubiculum, layer 2	8.25	1.88
Orbital area, ventrolateral part, layer 5	0.40	0.09
Posterolateral visual area, layer 6a	10.51	2.41
Anterolateral visual area, layer 6a	8.13	1.88
Entorhinal area, medial part, dorsal zone, layer 6	8.59	1.99
Parasubiculum, layer 2	8.48	1.96
Parasubiculum, layer 3	9.39	2.18
Primary motor area, Layer 2/3	4.48	1.05
Primary visual area, layer 1	3.95	0.93
Continued Table S3		

Entorhinal area, medial part, dorsal zone, layer 5	8.42	1.98
Primary somatosensory area, upper limb, layer 1	6.35	1.50
posteromedial visual area, layer 6a	8.07	1.93
Entorhinal area, medial part, dorsal zone, layer 4	8.44	2.02
Primary visual area, layer 6a	6.15	1.48
Ventral part of the lateral geniculate complex	5.05	1.22
Posterolateral visual area, layer 6b	9.44	2.27
Midbrain	1.18	0.29
Superior colliculus, motor related, intermediate gray layer, sublayer b	6.38	1.54
Medial geniculate complex, dorsal part	6.40	1.55
Postsubiculum, layer 2	6.62	1.60
Parasubiculum, layer 1	6.86	1.66
Presubiculum, layer 3	7.37	1.79
Primary motor area, Layer 1	3.38	0.82
Postsubiculum, layer 3	6.56	1.62
Zona incerta	1.93	0.48
posteromedial visual area, layer 5	6.88	1.71
Lateral visual area, layer 6b	8.82	2.21
Entorhinal area, medial part, dorsal zone, layer 3	7.28	1.83
Paracentral nucleus	1.94	0.49
Superior colliculus, motor related, intermediate white layer	6.25	1.57
posteromedial visual area, layer 2/3	4.01	1.01
Primary somatosensory area, trunk, layer 2/3	4.02	1.02
Orbital area, lateral part, layer 6a	0.82	0.21
Retrosplenial area, dorsal part, layer 5	2.18	0.56
Entorhinal area, medial part, dorsal zone, layer 2	6.28	1.60
Anterolateral visual area, layer 6b	5.79	1.48

Continued Table S3

Entorhinal area, medial part, dorsal zone, layer 1	4.12	1.06
Superior colliculus, motor related, intermediate gray layer, sublayer a	7.54	1.94
Primary somatosensory area, trunk, layer 6a	1.61	0.41
Suprageniculate nucleus	6.12	1.58
Anterior pretectal nucleus	4.88	1.26
posteromedial visual area, layer 4	5.54	1.43
Primary somatosensory area, trunk, layer 4	3.81	0.99
Retrosplenial area, dorsal part, layer 6a	2.88	0.75
Nucleus of the optic tract	6.80	1.77
posteromedial visual area, layer 6b	7.07	1.84
Superior colliculus, motor related, intermediate gray layer, sublayer c	4.25	1.11
Retrosplenial area, dorsal part, layer 2/3	1.36	0.36
Entorhinal area, medial part, ventral zone, layer 2	5.95	1.57
Peripeduncular nucleus	2.71	0.72
Posterolateral visual area, layer 1	3.55	0.95
Primary visual area, layer 6b	4.10	1.10
Posterior limiting nucleus of the thalamus	3.44	0.92
Intergeniculate leaflet of the lateral geniculate complex	5.25	1.41
Lateral habenula	2.96	0.80
Superior colliculus, motor related, deep gray layer	3.32	0.90
Superior colliculus, motor related, intermediate gray layer	5.38	1.47
Entorhinal area, medial part, ventral zone, layer 5/6	7.88	2.16
Anterior olfactory nucleus, posteroventral part	1.30	0.36
Primary motor area, Layer 5	2.33	0.64
Subthalamic nucleus	1.62	0.45
Posterior pretectal nucleus	6.73	1.85

Continued Table S3

Olivary pretectal nucleus	5.61	1.56
Primary somatosensory area, trunk, layer 1	3.14	0.87
Postsubiculum, layer 1	4.57	1.28
Mediodorsal nucleus of the thalamus, central part	3.40	0.95
Entorhinal area, medial part, ventral zone, layer 3	6.13	1.72
Primary somatosensory area, trunk, layer 5	2.69	0.76
Primary somatosensory area, lower limb, layer 4	2.71	0.77
Primary somatosensory area, upper limb, layer 6b	3.58	1.02
Medial geniculate complex, medial part	2.88	0.82
posteromedial visual area, layer 1	2.48	0.71
Retrosplenial area, dorsal part, layer 6b	1.41	0.41
Subparafascicular nucleus, parvicellular part	1.12	0.33
Primary somatosensory area, lower limb, layer 2/3	2.63	0.77
Entorhinal area, medial part, ventral zone, layer 1	2.37	0.69
Retrosplenial area, dorsal part, layer 1	0.77	0.22
Entorhinal area, medial part, dorsal zone, layer 2b	6.28	1.85
Superior colliculus, optic layer	4.35	1.30
Superior colliculus, motor related, deep white layer	2.25	0.68
Retrosplenial area, lateral agranular part, layer 6a	2.74	0.84
Orbital area, lateral part, layer 1	1.08	0.33
Retrosplenial area, lateral agranular part, layer 5	2.39	0.73
Parafascicular nucleus	0.25	0.08
Primary motor area, Layer 6b	0.29	0.09
Orbital area, ventrolateral part, layer 6a	0.62	0.19
Primary somatosensory area, lower limb, layer 5	2.12	0.66
Anteromedial nucleus, dorsal part	1.40	0.44
Retrosplenial area, ventral part, layer 2/3	2.05	0.65

Continued Table S3

Primary somatosensory area, lower limb, layer 1	1.62	0.52
Superior colliculus, zonal layer	3.55	1.14
Retrosplenial area, ventral part, layer 5	1.75	0.56
Superior colliculus, superficial gray layer	4.28	1.38
Medial habenula	0.53	0.17
Lateral terminal nucleus of the accessory optic tract	1.19	0.39
Interanterodorsal nucleus of the thalamus	1.82	0.60
Inferior colliculus, external nucleus	1.02	0.34
Retrosplenial area, ventral part, layer 1	1.98	0.66
Retrosplenial area, lateral agranular part, layer 6b	2.08	0.70
Periaqueductal gray	0.36	0.12
Retrosplenial area, ventral part, layer 2	1.91	0.64
Substantia nigra, reticular part	0.76	0.26
Midbrain reticular nucleus	0.11	0.04
Retrosplenial area, ventral part, layer 6a	2.02	0.71
Entorhinal area, medial part, dorsal zone, layer 2a	3.93	1.38
Anteromedial visual area, layer 4	1.55	0.55
Anteromedial visual area, layer 5	0.96	0.34
Retrosplenial area, lateral agranular part, layer 2/3	1.30	0.46
Medial pretectal area	1.17	0.42
Primary somatosensory area, lower limb, layer 6a	1.47	0.53
Fasciola cinerea	1.27	0.47
Anteromedial visual area, layer 6b	0.46	0.17
Primary motor area, Layer 6a	0.76	0.29
Mediodorsal nucleus of the thalamus, medial part	0.87	0.33
Simple lobule, molecular layer	0.18	0.07
Nucleus of the posterior commissure	0.62	0.24
Continued Table S3		

Bed nuclei of the stria terminalis, anterior division, anterolateral area	0.85	0.33
Lateral septal nucleus, caudal (caudodorsal) part	0.64	0.25
Primary somatosensory area, lower limb, layer 6b	0.69	0.27
Subgeniculate nucleus	2.65	1.04
Substantia nigra, compact part	0.75	0.30
Secondary motor area, layer 1	0.33	0.14
Secondary motor area, layer 2/3	0.34	0.14
Orbital area, ventrolateral part, layer 2/3	0.24	0.10
Retrosplenial area, ventral part, layer 6b	1.02	0.43
Anteromedial visual area, layer 2/3	1.57	0.66
Precommissural nucleus	0.05	0.02
Flocculus, molecular layer	0.09	0.04
Anteromedial visual area, layer 6a	0.41	0.19
Cerebellum	0.01	0.00
Bed nuclei of the stria terminalis, anterior division, oval nucleus	1.31	0.60
Paraventricular nucleus of the thalamus	0.34	0.16
Pons	0.03	0.01
Nucleus of the brachium of the inferior colliculus	1.50	0.71
Lateral preoptic area	0.41	0.19
Lateral septal nucleus, rostral (rostroventral) part	0.12	0.06
Simple lobule, granular layer	0.09	0.04
Lobules IV-V, molecular layer	0.03	0.01
Bed nuclei of the stria terminalis, anterior division, juxtacapsular nucleus	2.05	1.02
Retrosplenial area, lateral agranular part, layer 1	0.39	0.20

Table S3 Regions with increased T2-intensities in MRI.
Edema/damage is presented as lesion percentage, meaning percentage of a given region that showed a lesion in tT2w-MR images.

**Label**	**Rank**	**Importance**
Caudoputamen	1	0.32308629
genu of corpus callosum	2	0.2332126
Magnocellular nucleus	3	0.21865599
Primary somatosensory area, mouth, layer 6b	4	0.18925805
cranial nerves	5	0.18675973
Substantia innominata	6	0.1851075
Basic cell groups and regions	7	0.17853399
Striatum-like amygdalar nuclei	8	0.16087243
Primary somatosensory area, barrel field, layer 6b	9	0.15391005
Basolateral amygdalar nucleus, anterior part	10	0.15348099
Central amygdalar nucleus, capsular part	11	0.1428494
Islands of Calleja	12	0.13133924
Gustatory areas, layer 6b	13	0.12603834
fiber tracts	14	0.12539283
Primary somatosensory area, nose, layer 6b	15	0.11755629
supra-callosal cerebral white matter	16	0.11395881
corpus callosum body	17	0.11391755
Fundus of striatum	18	0.10824129
Olfactory tubercle, polymorph layer	19	0.10756712
Agranular insular area, dorsal part, layer 6a	20	0.10229244
Entorhinal area, lateral part, layer 6b	21	0.10175451
Supplemental somatosensory area, layer 2/3	22	0.09889486
anterior commissure temporal limb	23	0.09201019
Primary somatosensory area, nose, layer 6a	24	0.09112598
corpus callosum	25	0.08295043

Continued Table S4

Intercalated amygdalar nucleus	26	0.07695747
Claustrum	27	0.07074763
lateral forebrain bundle system	28	0.06814444
external capsule	29	0.06672324
Gustatory areas, layer 1	30	0.06626382
Ventral auditory area, layer 2/3	31	0.06552788
Primary somatosensory area, unassigned, layer 6a	32	0.06303901
Agranular insular area, ventral part, layer 5	33	0.06251169
Olfactory tubercle, pyramidal layer	34	0.06074569
Visceral area, layer 4	35	0.06024039
Ventral auditory area, layer 4	36	0.06023796
Primary somatosensory area, barrel field, layer 6a	37	0.0600351
cerebrum related	38	0.05941571
Gustatory areas, layer 6a	39	0.05928908
Entorhinal area, lateral part, layer 6a	40	0.05552282
Agranular insular area, dorsal part, layer 6b	41	0.05478822
Anterior amygdalar area	42	0.05442298
Ectorhinal area/Layer 5	43	0.05199313
corticospinal tract	44	0.05029466
Agranular insular area, posterior part, layer 1	45	0.04948235
Temporal association areas, layer 1	46	0.04874207
medial forebrain bundle system	47	0.04870997
Supplemental somatosensory area, layer 4	48	0.04857274
Agranular insular area, dorsal part, layer 5	49	0.04831752
Supplemental somatosensory area, layer 6b	50	0.04770422

Continued Table S4

amygdalar capsule	51	0.04683343
Anterolateral visual area, layer 2/3	52	0.04644172
Visceral area, layer 2/3	53	0.04627575
Visceral area, layer 1	54	0.0451319
Endopiriform nucleus, ventral part	55	0.045062
Gustatory areas, layer 5	56	0.04502237
Visceral area, layer 6b	57	0.04488031
corpus callosum anterior forceps	58	0.04487091
Diagonal band nucleus	59	0.04485613
Agranular insular area, posterior part, layer 6a	60	0.04373156
Temporal association areas, layer 4	61	0.04304704
Supplemental somatosensory area, layer 1	62	0.04086983
alveus	63	0.03928596
internal capsule	64	0.03884521
Ectorhinal area/Layer 6b	65	0.03723898
Primary motor area, Layer 5	66	0.03679916
Agranular insular area, ventral part, layer 6a	67	0.03597981
Agranular insular area, dorsal part, layer 2/3	68	0.03149397
Piriform area, pyramidal layer	69	0.02824495
Field CA3, stratum oriens	70	0.02097705
Primary somatosensory area, unassigned, layer 5	71	0.02072315
Agranular insular area, posterior part, layer 2/3	72	0.01995215
Field CA3, pyramidal layer	73	0.01334997
Globus pallidus, internal segment	74	0.01334802
Agranular insular area, dorsal part, layer 1	75	0.01082569
Central amygdalar nucleus, medial part	76	0.01056376

Continued Table S4

Central amygdalar nucleus, lateral part	77	0.00766024
Nucleus accumbens	78	0.00516727
optic tract	79	0.00468865
Ventral auditory area, layer 5	80	0.00264418
Olfactory tubercle, molecular layer	81	0.00103826

Table S4 Regions with out-of-bag importance above 0.

A

Tukey's multiple comparisons test	Mean Diff.	95% CI of Diff.	Adjusted P Value
Mild vs. Moderate	-12.26	-26.31 to 1.794	0.1185
Mild vs. Moderate to Mild	-3.136	-14.88 to 8.613	0.9474
Mild vs. Severe	-33.2	-47.03 to -19.38	<0.0001
Mild vs. Severe to Moderate	-19.59	-32.07 to -7.097	0.0003
Moderate vs. Moderate to Mild	9.124	-5.302 to 23.55	0.4085
Moderate vs. Severe	-20.94	-37.10 to -4.780	0.0042
Moderate vs. Severe to Moderate	-7.326	-22.36 to 7.708	0.6628
Moderate to Mild vs. Severe	-30.06	-44.27 to -15.86	<0.0001
Moderate to Mild vs. Severe to Moderate	-16.45	-29.36 to -3.543	0.0051
Severe vs. Severe to Moderate	13.61	-1.207 to 28.44	0.088

B

Test details	Mean 1	Mean 2	Mean Diff.	SE of Diff.
Mild vs. Moderate	14.03	26.29	-12.26	5.086
Mild vs. Moderate to Mild	14.03	17.17	-3.136	4.252
Mild vs. Severe	14.03	47.23	-33.2	5.004
Mild vs. Severe to Moderate	14.03	33.62	-19.59	4.52
Moderate vs. Moderate to Mild	26.29	17.17	9.124	5.221
Moderate vs. Severe	26.29	47.23	-20.94	5.849
Moderate vs. Severe to Moderate	26.29	33.62	-7.326	5.442
Moderate to Mild vs. Severe	17.17	47.23	-30.06	5.141
Moderate to Mild vs. Severe to Moderate	17.17	33.62	-16.45	4.671
Severe vs. Severe to Moderate	47.23	33.62	13.61	5.364

Table S5 Statistical report on the analysis of severity grades and stroke lesion volumes (A,B)

**Šídák's multiple comparisons test**	**Mean Diff.**	**95% CI of Diff.**	**Adjusted P Value**
Mild: Lesion Volume vs. Mild: MRI [Segmented]	-13.99	-14.98 to -12.99	<0.0001
Mild: Lesion Volume vs. Moderate to Mild: Lesion Volume	7.513	6.521 to 8.505	<0.0001
Mild: Lesion Volume vs. Moderate to Mild: MRI [Segmented]	10.17	9.181 to 11.17	<0.0001
Mild: Lesion Volume vs. Moderate: Lesion Volume	-0.6437	-1.636 to 0.3482	0.7907
Mild: Lesion Volume vs. Moderate: MRI [Segmented]	6.225	5.233 to 7.217	<0.0001
Mild: Lesion Volume vs. Moderate to Severe: Lesion Volume	11.01	10.02 to 12.00	<0.0001
Mild: Lesion Volume vs. Moderate to Severe: MRI [Segmented]	12.51	11.51 to 13.50	<0.0001
Mild: Lesion Volume vs. Severe: Lesion Volume	13.12	12.12 to 14.11	<0.0001
Mild: Lesion Volume vs. Severe: MRI [Segmented]	11.4	10.41 to 12.40	<0.0001
Mild: MRI [Segmented] vs. Moderate to Mild: Lesion Volume	21.5	20.51 to 22.49	<0.0001
Mild: MRI [Segmented] vs. Moderate to Mild: MRI [Segmented]	24.16	23.17 to 25.15	<0.0001
Mild: MRI [Segmented] vs. Moderate: Lesion Volume	13.34	12.35 to 14.33	<0.0001
Mild: MRI [Segmented] vs. Moderate: MRI [Segmented]	20.21	19.22 to 21.20	<0.0001
Mild: MRI [Segmented] vs. Moderate to Severe: Lesion Volume	24.99	24.00 to 25.99	<0.0001
Mild: MRI [Segmented] vs. Moderate to Severe: MRI [Segmented]	26.49	25.50 to 27.48	<0.0001
Mild: MRI [Segmented] vs. Severe: Lesion Volume	27.1	26.11 to 28.09	<0.0001
Mild: MRI [Segmented] vs. Severe: MRI [Segmented]	25.39	24.40 to 26.38	<0.0001
Moderate to Mild: Lesion Volume vs. Moderate to Mild: MRI [Segmented]	2.66	1.668 to 3.652	<0.0001
Moderate to Mild: Lesion Volume vs. Moderate: Lesion Volume	-8.157	-9.149 to -7.165	<0.0001
Moderate to Mild: Lesion Volume vs. Moderate: MRI [Segmented]	-1.289	-2.281 to -0.2968	0.0011
Moderate to Mild: Lesion Volume vs. Moderate to Severe: Lesion Volume	3.494	2.502 to 4.486	<0.0001

Continued Table S6

Moderate to Mild: Lesion Volume vs. Moderate to Severe: MRI [Segmented]	4.992	4.000 to 5.984	<0.0001
Moderate to Mild: Lesion Volume vs. Severe: Lesion Volume	5.602	4.610 to 6.594	<0.0001
Moderate to Mild: Lesion Volume vs. Severe: MRI [Segmented]	3.891	2.899 to 4.883	<0.0001
Moderate to Mild: MRI [Segmented] vs. Moderate: Lesion Volume	-10.82	-11.81 to -9.825	<0.0001
Moderate to Mild: MRI [Segmented] vs. Moderate: MRI [Segmented]	-3.949	-4.941 to -2.957	<0.0001
Moderate to Mild: MRI [Segmented] vs. Moderate to Severe: Lesion Volume	0.834	-0.1579 to 1.826	0.2421
Moderate to Mild: MRI [Segmented] vs. Moderate to Severe: MRI [Segmented]	2.332	1.340 to 3.324	<0.0001
Moderate to Mild: MRI [Segmented] vs. Severe: Lesion Volume	2.942	1.950 to 3.934	<0.0001
Moderate to Mild: MRI [Segmented] vs. Severe: MRI [Segmented]	1.231	0.2395 to 2.223	0.0025
Moderate: Lesion Volume vs. Moderate: MRI [Segmented]	6.868	5.876 to 7.860	<0.0001
Moderate: Lesion Volume vs. Moderate to Severe: Lesion Volume	11.65	10.66 to 12.64	<0.0001
Moderate: Lesion Volume vs. Moderate to Severe: MRI [Segmented]	13.15	12.16 to 14.14	<0.0001
Moderate: Lesion Volume vs. Severe: Lesion Volume	13.76	12.77 to 14.75	<0.0001
Moderate: Lesion Volume vs. Severe: MRI [Segmented]	12.05	11.06 to 13.04	<0.0001
Moderate: MRI [Segmented] vs. Moderate to Severe: Lesion Volume	4.783	3.791 to 5.775	<0.0001
Moderate: MRI [Segmented] vs. Moderate to Severe: MRI [Segmented]	6.281	5.289 to 7.273	<0.0001
Moderate: MRI [Segmented] vs. Severe: Lesion Volume	6.891	5.899 to 7.883	<0.0001
Moderate: MRI [Segmented] vs. Severe: MRI [Segmented]	5.18	4.188 to 6.172	<0.0001
Moderate to Severe: Lesion Volume vs. Moderate to Severe: MRI [Segmented]	1.498	0.5063 to 2.490	<0.0001
Moderate to Severe: Lesion Volume vs. Severe: Lesion Volume	2.108	1.116 to 3.100	<0.0001
Continued Table S6			

Moderate to Severe: Lesion Volume vs. Severe: MRI [Segmented]	0.3974	-0.5945 to 1.389	>0.9999
Moderate to Severe: MRI [Segmented] vs. Severe: Lesion Volume	0.6101	-0.3819 to 1.602	0.8721
Moderate to Severe: MRI [Segmented] vs. Severe: MRI [Segmented]	-1.101	-2.093 to -0.1088	0.0139
Severe: Lesion Volume vs. Severe: MRI [Segmented]	-1.711	-2.703 to -0.7189	<0.0001

**Test details**	**Mean 1**	**Mean 2**	**Mean Diff.**	**SE of Diff.**	**t**	**DF**
Mild: Lesion Volume vs. Mild: MRI [Segmented]	23.99	37.97	-13.99	0.3031	46.15	490
Mild: Lesion Volume vs. Moderate to Mild: Lesion Volume	23.99	16.47	7.513	0.3031	24.79	490
Mild: Lesion Volume vs. Moderate to Mild: MRI [Segmented]	23.99	13.82	10.17	0.3031	33.57	490
Mild: Lesion Volume vs. Moderate: Lesion Volume	23.99	24.63	-0.6437	0.3031	2.124	490
Mild: Lesion Volume vs. Moderate: MRI [Segmented]	23.99	17.76	6.225	0.3031	20.54	490
Mild: Lesion Volume vs. Moderate to Severe: Lesion Volume	23.99	12.98	11.01	0.3031	36.32	490
Mild: Lesion Volume vs. Moderate to Severe: MRI [Segmented]	23.99	11.48	12.51	0.3031	41.26	490
Mild: Lesion Volume vs. Severe: Lesion Volume	23.99	10.87	13.12	0.3031	43.28	490
Mild: Lesion Volume vs. Severe: MRI [Segmented]	23.99	12.58	11.4	0.3031	37.63	490
Mild: MRI [Segmented] vs. Moderate to Mild: Lesion Volume	37.97	16.47	21.5	0.3031	70.94	490
Mild: MRI [Segmented] vs. Moderate to Mild: MRI [Segmented]	37.97	13.82	24.16	0.3031	79.72	490
Mild: MRI [Segmented] vs. Moderate: Lesion Volume	37.97	24.63	13.34	0.3031	44.02	490
Mild: MRI [Segmented] vs. Moderate: MRI [Segmented]	37.97	17.76	20.21	0.3031	66.69	490

Continued Table S6

Mild: MRI [Segmented] vs. Moderate to Severe: Lesion Volume	37.97	12.98	24.99	0.3031	82.47	490
Mild: MRI [Segmented] vs. Moderate to Severe: MRI [Segmented]	37.97	11.48	26.49	0.3031	87.41	490
Mild: MRI [Segmented] vs. Severe: Lesion Volume	37.97	10.87	27.1	0.3031	89.42	490
Mild: MRI [Segmented] vs. Severe: MRI [Segmented]	37.97	12.58	25.39	0.3031	83.78	490
Moderate to Mild: Lesion Volume vs. Moderate to Mild: MRI [Segmented]	16.47	13.82	2.66	0.3031	8.777	490
Moderate to Mild: Lesion Volume vs. Moderate: Lesion Volume	16.47	24.63	-8.157	0.3031	26.92	490
Moderate to Mild: Lesion Volume vs. Moderate: MRI [Segmented]	16.47	17.76	-1.289	0.3031	4.252	490
Moderate to Mild: Lesion Volume vs. Moderate to Severe: Lesion Volume	16.47	12.98	3.494	0.3031	11.53	490
Moderate to Mild: Lesion Volume vs. Moderate to Severe: MRI [Segmented]	16.47	11.48	4.992	0.3031	16.47	490
Moderate to Mild: Lesion Volume vs. Severe: Lesion Volume	16.47	10.87	5.602	0.3031	18.49	490
Moderate to Mild: Lesion Volume vs. Severe: MRI [Segmented]	16.47	12.58	3.891	0.3031	12.84	490
Moderate to Mild: MRI [Segmented] vs. Moderate: Lesion Volume	13.82	24.63	-10.82	0.3031	35.69	490
Moderate to Mild: MRI [Segmented] vs. Moderate: MRI [Segmented]	13.82	17.76	-3.949	0.3031	13.03	490
Moderate to Mild: MRI [Segmented] vs. Moderate to Severe: Lesion Volume	13.82	12.98	0.834	0.3031	2.752	490
Moderate to Mild: MRI [Segmented] vs. Moderate to Severe: MRI [Segmented]	13.82	11.48	2.332	0.3031	7.696	490

Continued Table S6

Moderate to Mild: MRI [Segmented] vs. Severe: Lesion Volume	13.82	10.87	2.942	0.3031	9.708	490
Moderate to Mild: MRI [Segmented] vs. Severe: MRI [Segmented]	13.82	12.58	1.231	0.3031	4.063	490
Moderate: Lesion Volume vs. Moderate: MRI [Segmented]	24.63	17.76	6.868	0.3031	22.66	490
Moderate: Lesion Volume vs. Moderate to Severe: Lesion Volume	24.63	12.98	11.65	0.3031	38.44	490
Moderate: Lesion Volume vs. Moderate to Severe: MRI [Segmented]	24.63	11.48	13.15	0.3031	43.39	490
Moderate: Lesion Volume vs. Severe: Lesion Volume	24.63	10.87	13.76	0.3031	45.4	490
Moderate: Lesion Volume vs. Severe: MRI [Segmented]	24.63	12.58	12.05	0.3031	39.76	490
Moderate: MRI [Segmented] vs. Moderate to Severe: Lesion Volume	17.76	12.98	4.783	0.3031	15.78	490
Moderate: MRI [Segmented] vs. Moderate to Severe: MRI [Segmented]	17.76	11.48	6.281	0.3031	20.72	490
Moderate: MRI [Segmented] vs. Severe: Lesion Volume	17.76	10.87	6.891	0.3031	22.74	490
Moderate: MRI [Segmented] vs. Severe: MRI [Segmented]	17.76	12.58	5.18	0.3031	17.09	490
Moderate to Severe: Lesion Volume vs. Moderate to Severe: MRI [Segmented]	12.98	11.48	1.498	0.3031	4.943	490
Moderate to Severe: Lesion Volume vs. Severe: Lesion Volume	12.98	10.87	2.108	0.3031	6.956	490
Moderate to Severe: Lesion Volume vs. Severe: MRI [Segmented]	12.98	12.58	0.3974	0.3031	1.311	490
Moderate to Severe: MRI [Segmented] vs. Severe: Lesion Volume	11.48	10.87	0.6101	0.3031	2.013	490
Continued Table S6						

Moderate to Severe: MRI [Segmented] vs. Severe: MRI [Segmented]	11.48	12.58	-1.101	0.3031	3.632	490
Severe: Lesion Volume vs. Severe: MRI [Segmented]	10.87	12.58	-1.711	0.3031	5.645	490

Table S6 Full statistical report on the analysis of prediction accuracy and severity grade for the subacute deficit.

**Šídák's multiple comparisons test**	**Mean Diff.**	**95% CI of Diff.**	**Adjusted P Value**
Mild: Subacute Deficit vs. Mild: MRI [Segmented]	-3.853	-5.602 to -2.105	<0.0001
Mild: Subacute Deficit vs. Mild: Lesion Volume	-5.732	-7.480 to -3.984	<0.0001
Mild: Subacute Deficit vs. Moderate to Mild: Subacute Deficit	12.16	10.41 to 13.90	<0.0001
Mild: Subacute Deficit vs. Moderate to Mild: MRI [Segmented]	16.56	14.81 to 18.31	<0.0001
Mild: Subacute Deficit vs. Moderate to Mild: Lesion Volume	15.28	13.53 to 17.03	<0.0001
Mild: Subacute Deficit vs. Moderate: Subacute Deficit	22.54	20.79 to 24.28	<0.0001
Mild: Subacute Deficit vs. Moderate: MRI [Segmented]	8.424	6.676 to 10.17	<0.0001
Mild: Subacute Deficit vs. Moderate: Lesion Volume	1.963	0.2143 to 3.711	0.0096
Mild: Subacute Deficit vs. Moderate to Severe: Subacute Deficit	17.2	15.45 to 18.94	<0.0001
Mild: Subacute Deficit vs. Moderate to Severe: MRI [Segmented]	2.834	1.085 to 4.582	<0.0001
Mild: Subacute Deficit vs. Moderate to Severe: Lesion Volume	2.337	0.5887 to 4.085	0.0004
Mild: Subacute Deficit vs. Severe: Subacute Deficit	4.231	2.483 to 5.980	<0.0001
Mild: Subacute Deficit vs. Severe: MRI [Segmented]	-8.166	-9.914 to -6.417	<0.0001
Mild: Subacute Deficit vs. Severe: Lesion Volume	-11.03	-12.78 to -9.283	<0.0001
Mild: MRI [Segmented] vs. Mild: Lesion Volume	-1.879	-3.627 to -0.1305	0.0188
Mild: MRI [Segmented] vs. Moderate to Mild: Subacute Deficit	16.01	14.26 to 17.76	<0.0001
Mild: MRI [Segmented] vs. Moderate to Mild: MRI [Segmented]	20.41	18.67 to 22.16	<0.0001
Mild: MRI [Segmented] vs. Moderate to Mild: Lesion Volume	19.13	17.38 to 20.88	<0.0001
Mild: MRI [Segmented] vs. Moderate: Subacute Deficit	26.39	24.64 to 28.14	<0.0001
Mild: MRI [Segmented] vs. Moderate: MRI [Segmented]	12.28	10.53 to 14.03	<0.0001
Mild: MRI [Segmented] vs. Moderate: Lesion Volume	5.816	4.068 to 7.564	<0.0001

Continued Table S7

Mild: MRI [Segmented] vs. Moderate to Severe: Subacute Deficit	21.05	19.30 to 22.80	<0.0001
Mild: MRI [Segmented] vs. Moderate to Severe: MRI [Segmented]	6.687	4.939 to 8.435	<0.0001
Mild: MRI [Segmented] vs. Moderate to Severe: Lesion Volume	6.19	4.442 to 7.939	<0.0001
Mild: MRI [Segmented] vs. Severe: Subacute Deficit	8.085	6.336 to 9.833	<0.0001
Mild: MRI [Segmented] vs. Severe: MRI [Segmented]	-4.312	-6.061 to -2.564	<0.0001
Mild: MRI [Segmented] vs. Severe: Lesion Volume	-7.178	-8.927 to -5.430	<0.0001
Mild: Lesion Volume vs. Moderate to Mild: Subacute Deficit	17.89	16.14 to 19.64	<0.0001
Mild: Lesion Volume vs. Moderate to Mild: MRI [Segmented]	22.29	20.54 to 24.04	<0.0001
Mild: Lesion Volume vs. Moderate to Mild: Lesion Volume	21.01	19.26 to 22.76	<0.0001
Mild: Lesion Volume vs. Moderate: Subacute Deficit	28.27	26.52 to 30.02	<0.0001
Mild: Lesion Volume vs. Moderate: MRI [Segmented]	14.16	12.41 to 15.90	<0.0001
Mild: Lesion Volume vs. Moderate: Lesion Volume	7.695	5.946 to 9.443	<0.0001
Mild: Lesion Volume vs. Moderate to Severe: Subacute Deficit	22.93	21.18 to 24.68	<0.0001
Mild: Lesion Volume vs. Moderate to Severe: MRI [Segmented]	8.566	6.818 to 10.31	<0.0001
Mild: Lesion Volume vs. Moderate to Severe: Lesion Volume	8.069	6.321 to 9.817	<0.0001
Mild: Lesion Volume vs. Severe: Subacute Deficit	9.963	8.215 to 11.71	<0.0001
Mild: Lesion Volume vs. Severe: MRI [Segmented]	-2.434	-4.182 to -0.6853	0.0001
Mild: Lesion Volume vs. Severe: Lesion Volume	-5.3	-7.048 to -3.551	<0.0001
Moderate to Mild: Subacute Deficit vs. Moderate to Mild: MRI [Segmented]	4.406	2.657 to 6.154	<0.0001
Moderate to Mild: Subacute Deficit vs. Moderate to Mild: Lesion Volume	3.122	1.374 to 4.870	<0.0001
Moderate to Mild: Subacute Deficit vs. Moderate: Subacute Deficit	10.38	8.631 to 12.13	<0.0001
Moderate to Mild: Subacute Deficit vs. Moderate: MRI [Segmented]	-3.731	-5.480 to -1.983	<0.0001

Continued Table S7

Moderate to Mild: Subacute Deficit vs. Moderate: Lesion Volume	-10.19	-11.94 to -8.445	<0.0001
Moderate to Mild: Subacute Deficit vs. Moderate to Severe: Subacute Deficit	5.04	3.292 to 6.789	<0.0001
Moderate to Mild: Subacute Deficit vs. Moderate to Severe: MRI [Segmented]	-9.322	-11.07 to -7.574	<0.0001
Moderate to Mild: Subacute Deficit vs. Moderate to Severe: Lesion Volume	-9.819	-11.57 to -8.070	<0.0001
Moderate to Mild: Subacute Deficit vs. Severe: Subacute Deficit	-7.924	-9.672 to -6.176	<0.0001
Moderate to Mild: Subacute Deficit vs. Severe: MRI [Segmented]	-20.32	-22.07 to -18.57	<0.0001
Moderate to Mild: Subacute Deficit vs. Severe: Lesion Volume	-23.19	-24.94 to -21.44	<0.0001
Moderate to Mild: MRI [Segmented] vs. Moderate to Mild: Lesion Volume	-1.284	-3.032 to 0.4647	0.6635
Moderate to Mild: MRI [Segmented] vs. Moderate: Subacute Deficit	5.974	4.226 to 7.722	<0.0001
Moderate to Mild: MRI [Segmented] vs. Moderate: MRI [Segmented]	-8.137	-9.885 to -6.389	<0.0001
Moderate to Mild: MRI [Segmented] vs. Moderate: Lesion Volume	-14.6	-16.35 to -12.85	<0.0001
Moderate to Mild: MRI [Segmented] vs. Moderate to Severe: Subacute Deficit	0.6347	-1.114 to 2.383	>0.9999
Moderate to Mild: MRI [Segmented] vs. Moderate to Severe: MRI [Segmented]	-13.73	-15.48 to -11.98	<0.0001
Moderate to Mild: MRI [Segmented] vs. Moderate to Severe: Lesion Volume	-14.22	-15.97 to -12.48	<0.0001
Moderate to Mild: MRI [Segmented] vs. Severe: Subacute Deficit	-12.33	-14.08 to -10.58	<0.0001
Moderate to Mild: MRI [Segmented] vs. Severe: MRI [Segmented]	-24.73	-26.48 to -22.98	<0.0001
Moderate to Mild: MRI [Segmented] vs. Severe: Lesion Volume	-27.59	-29.34 to -25.84	<0.0001
Moderate to Mild: Lesion Volume vs. Moderate: Subacute Deficit	7.258	5.509 to 9.006	<0.0001

Continued Table S7

Moderate to Mild: Lesion Volume vs. Moderate: MRI [Segmented]	-6.853	-8.602 to -5.105	<0.0001
Moderate to Mild: Lesion Volume vs. Moderate: Lesion Volume	-13.32	-15.06 to -11.57	<0.0001
Moderate to Mild: Lesion Volume vs. Moderate to Severe: Subacute Deficit	1.918	0.1700 to 3.667	0.0138
Moderate to Mild: Lesion Volume vs. Moderate to Severe: MRI [Segmented]	-12.44	-14.19 to -10.70	<0.0001
Moderate to Mild: Lesion Volume vs. Moderate to Severe: Lesion Volume	-12.94	-14.69 to -11.19	<0.0001
Moderate to Mild: Lesion Volume vs. Severe: Subacute Deficit	-11.05	-12.79 to -9.298	<0.0001
Moderate to Mild: Lesion Volume vs. Severe: MRI [Segmented]	-23.44	-25.19 to -21.70	<0.0001
Moderate to Mild: Lesion Volume vs. Severe: Lesion Volume	-26.31	-28.06 to -24.56	<0.0001
Moderate: Subacute Deficit vs. Moderate: MRI [Segmented]	-14.11	-15.86 to -12.36	<0.0001
Moderate: Subacute Deficit vs. Moderate: Lesion Volume	-20.57	-22.32 to -18.82	<0.0001
Moderate: Subacute Deficit vs. Moderate to Severe: Subacute Deficit	-5.339	-7.088 to -3.591	<0.0001
Moderate: Subacute Deficit vs. Moderate to Severe: MRI [Segmented]	-19.7	-21.45 to -17.95	<0.0001
Moderate: Subacute Deficit vs. Moderate to Severe: Lesion Volume	-20.2	-21.95 to -18.45	<0.0001
Moderate: Subacute Deficit vs. Severe: Subacute Deficit	-18.3	-20.05 to -16.56	<0.0001
Moderate: Subacute Deficit vs. Severe: MRI [Segmented]	-30.7	-32.45 to -28.95	<0.0001
Moderate: Subacute Deficit vs. Severe: Lesion Volume	-33.57	-35.32 to -31.82	<0.0001
Moderate: MRI [Segmented] vs. Moderate: Lesion Volume	-6.462	-8.210 to -4.713	<0.0001
Moderate: MRI [Segmented] vs. Moderate to Severe: Subacute Deficit	8.772	7.023 to 10.52	<0.0001
Moderate: MRI [Segmented] vs. Moderate to Severe: MRI [Segmented]	-5.591	-7.339 to -3.842	<0.0001
Continued Table S7			

Moderate: MRI [Segmented] vs. Moderate to Severe: Lesion Volume	-6.087	-7.836 to -4.339	<0.0001
Moderate: MRI [Segmented] vs. Severe: Subacute Deficit	-4.193	-5.941 to -2.445	<0.0001
Moderate: MRI [Segmented] vs. Severe: MRI [Segmented]	-16.59	-18.34 to -14.84	<0.0001
Moderate: MRI [Segmented] vs. Severe: Lesion Volume	-19.46	-21.20 to -17.71	<0.0001
Moderate: Lesion Volume vs. Moderate to Severe: Subacute Deficit	15.23	13.49 to 16.98	<0.0001
Moderate: Lesion Volume vs. Moderate to Severe: MRI [Segmented]	0.8712	-0.8771 to 2.619	0.9999
Moderate: Lesion Volume vs. Moderate to Severe: Lesion Volume	0.3745	-1.374 to 2.123	>0.9999
Moderate: Lesion Volume vs. Severe: Subacute Deficit	2.269	0.5205 to 4.017	0.0007
Moderate: Lesion Volume vs. Severe: MRI [Segmented]	-10.13	-11.88 to -8.380	<0.0001
Moderate: Lesion Volume vs. Severe: Lesion Volume	-12.99	-14.74 to -11.25	<0.0001
Moderate to Severe: Subacute Deficit vs. Moderate to Severe: MRI [Segmented]	-14.36	-16.11 to -12.61	<0.0001
Moderate to Severe: Subacute Deficit vs. Moderate to Severe: Lesion Volume	-14.86	-16.61 to -13.11	<0.0001
Moderate to Severe: Subacute Deficit vs. Severe: Subacute Deficit	-12.96	-14.71 to -11.22	<0.0001
Moderate to Severe: Subacute Deficit vs. Severe: MRI [Segmented]	-25.36	-27.11 to -23.61	<0.0001
Moderate to Severe: Subacute Deficit vs. Severe: Lesion Volume	-28.23	-29.98 to -26.48	<0.0001
Moderate to Severe: MRI [Segmented] vs. Moderate to Severe: Lesion Volume	-0.4967	-2.245 to 1.252	>0.9999
Moderate to Severe: MRI [Segmented] vs. Severe: Subacute Deficit	1.398	-0.3506 to 3.146	0.4241
Moderate to Severe: MRI [Segmented] vs. Severe: MRI [Segmented]	-11	-12.75 to -9.251	<0.0001
Moderate to Severe: MRI [Segmented] vs. Severe: Lesion Volume	-13.87	-15.61 to -12.12	<0.0001
Moderate to Severe: Lesion Volume vs. Severe: Subacute Deficit	1.894	0.1460 to 3.643	0.0166
Continued Table S7			

Moderate to Severe: Lesion Volume vs. Severe: MRI [Segmented]	-10.5	-12.25 to -8.754	<0.0001
Moderate to Severe: Lesion Volume vs. Severe: Lesion Volume	-13.37	-15.12 to -11.62	<0.0001
Severe: Subacute Deficit vs. Severe: MRI [Segmented]	-12.4	-14.15 to -10.65	<0.0001
Severe: Subacute Deficit vs. Severe: Lesion Volume	-15.26	-17.01 to -13.51	<0.0001
Severe: MRI [Segmented] vs. Severe: Lesion Volume	-2.866	-4.614 to -1.118	<0.0001

**Test details**	**Mean 1**	**Mean 2**	**Mean Diff.**	**SE of Diff.**	**N1**	**N2**	**t**	**DF**
Mild: Subacute Deficit vs. Mild: MRI [Segmented]	28.86	32.71	-3.853	0.4991	50	50	7.72	735
Mild: Subacute Deficit vs. Mild: Lesion Volume	28.86	34.59	-5.732	0.4991	50	50	11.48	735
Mild: Subacute Deficit vs. Moderate to Mild: Subacute Deficit	28.86	16.7	12.16	0.4991	50	50	24.35	735
Mild: Subacute Deficit vs. Moderate to Mild: MRI [Segmented]	28.86	12.3	16.56	0.4991	50	50	33.18	735
Mild: Subacute Deficit vs. Moderate to Mild: Lesion Volume	28.86	13.58	15.28	0.4991	50	50	30.61	735
Mild: Subacute Deficit vs. Moderate: Subacute Deficit	28.86	6.323	22.54	0.4991	50	50	45.15	735
Mild: Subacute Deficit vs. Moderate: MRI [Segmented]	28.86	20.43	8.424	0.4991	50	50	16.88	735
Mild: Subacute Deficit vs. Moderate: Lesion Volume	28.86	26.9	1.963	0.4991	50	50	3.932	735
Mild: Subacute Deficit vs. Moderate to Severe: Subacute Deficit	28.86	11.66	17.2	0.4991	50	50	34.45	735
Mild: Subacute Deficit vs. Moderate to Severe: MRI [Segmented]	28.86	26.02	2.834	0.4991	50	50	5.677	735
Mild: Subacute Deficit vs. Moderate to Severe: Lesion Volume	28.86	26.52	2.337	0.4991	50	50	4.682	735

Continued Table S7

Mild: Subacute Deficit vs. Severe: Subacute Deficit	28.86	24.63	4.231	0.4991	50	50	8.478	735
Mild: Subacute Deficit vs. Severe: MRI [Segmented]	28.86	37.02	-8.166	0.4991	50	50	16.36	735
Mild: Subacute Deficit vs. Severe: Lesion Volume	28.86	39.89	-11.03	0.4991	50	50	22.1	735
Mild: MRI [Segmented] vs. Mild: Lesion Volume	32.71	34.59	-1.879	0.4991	50	50	3.764	735
Mild: MRI [Segmented] vs. Moderate to Mild: Subacute Deficit	32.71	16.7	16.01	0.4991	50	50	32.07	735
Mild: MRI [Segmented] vs. Moderate to Mild: MRI [Segmented]	32.71	12.3	20.41	0.4991	50	50	40.9	735
Mild: MRI [Segmented] vs. Moderate to Mild: Lesion Volume	32.71	13.58	19.13	0.4991	50	50	38.33	735
Mild: MRI [Segmented] vs. Moderate: Subacute Deficit	32.71	6.323	26.39	0.4991	50	50	52.87	735
Mild: MRI [Segmented] vs. Moderate: MRI [Segmented]	32.71	20.43	12.28	0.4991	50	50	24.6	735
Mild: MRI [Segmented] vs. Moderate: Lesion Volume	32.71	26.9	5.816	0.4991	50	50	11.65	735
Mild: MRI [Segmented] vs. Moderate to Severe: Subacute Deficit	32.71	11.66	21.05	0.4991	50	50	42.17	735
Mild: MRI [Segmented] vs. Moderate to Severe: MRI [Segmented]	32.71	26.02	6.687	0.4991	50	50	13.4	735
Mild: MRI [Segmented] vs. Moderate to Severe: Lesion Volume	32.71	26.52	6.19	0.4991	50	50	12.4	735
Mild: MRI [Segmented] vs. Severe: Subacute Deficit	32.71	24.63	8.085	0.4991	50	50	16.2	735
Mild: MRI [Segmented] vs. Severe: MRI [Segmented]	32.71	37.02	-4.312	0.4991	50	50	8.64	735
Mild: MRI [Segmented] vs. Severe: Lesion Volume	32.71	39.89	-7.178	0.4991	50	50	14.38	735

Continued Table S7

Mild: Lesion Volume vs. Moderate to Mild: Subacute Deficit	34.59	16.7	17.89	0.4991	50	50	35.84	735
Mild: Lesion Volume vs. Moderate to Mild: MRI [Segmented]	34.59	12.3	22.29	0.4991	50	50	44.67	735
Mild: Lesion Volume vs. Moderate to Mild: Lesion Volume	34.59	13.58	21.01	0.4991	50	50	42.09	735
Mild: Lesion Volume vs. Moderate: Subacute Deficit	34.59	6.323	28.27	0.4991	50	50	56.63	735
Mild: Lesion Volume vs. Moderate: MRI [Segmented]	34.59	20.43	14.16	0.4991	50	50	28.36	735
Mild: Lesion Volume vs. Moderate: Lesion Volume	34.59	26.9	7.695	0.4991	50	50	15.42	735
Mild: Lesion Volume vs. Moderate to Severe: Subacute Deficit	34.59	11.66	22.93	0.4991	50	50	45.94	735
Mild: Lesion Volume vs. Moderate to Severe: MRI [Segmented]	34.59	26.02	8.566	0.4991	50	50	17.16	735
Mild: Lesion Volume vs. Moderate to Severe: Lesion Volume	34.59	26.52	8.069	0.4991	50	50	16.17	735
Mild: Lesion Volume vs. Severe: Subacute Deficit	34.59	24.63	9.963	0.4991	50	50	19.96	735
Mild: Lesion Volume vs. Severe: MRI [Segmented]	34.59	37.02	-2.434	0.4991	50	50	4.876	735
Mild: Lesion Volume vs. Severe: Lesion Volume	34.59	39.89	-5.3	0.4991	50	50	10.62	735
Moderate to Mild: Subacute Deficit vs. Moderate to Mild: MRI [Segmented]	16.7	12.3	4.406	0.4991	50	50	8.827	735
Moderate to Mild: Subacute Deficit vs. Moderate to Mild: Lesion Volume	16.7	13.58	3.122	0.4991	50	50	6.255	735
Moderate to Mild: Subacute Deficit vs. Moderate: Subacute Deficit	16.7	6.323	10.38	0.4991	50	50	20.8	735

Continued Table S7

Moderate to Mild: Subacute Deficit vs. Moderate: MRI [Segmented]	16.7	20.43	-3.731	0.4991	50	50	7.476	735
Moderate to Mild: Subacute Deficit vs. Moderate: Lesion Volume	16.7	26.9	-10.19	0.4991	50	50	20.42	735
Moderate to Mild: Subacute Deficit vs. Moderate to Severe: Subacute Deficit	16.7	11.66	5.04	0.4991	50	50	10.1	735
Moderate to Mild: Subacute Deficit vs. Moderate to Severe: MRI [Segmented]	16.7	26.02	-9.322	0.4991	50	50	18.68	735
Moderate to Mild: Subacute Deficit vs. Moderate to Severe: Lesion Volume	16.7	26.52	-9.819	0.4991	50	50	19.67	735
Moderate to Mild: Subacute Deficit vs. Severe: Subacute Deficit	16.7	24.63	-7.924	0.4991	50	50	15.88	735
Moderate to Mild: Subacute Deficit vs. Severe: MRI [Segmented]	16.7	37.02	-20.32	0.4991	50	50	40.71	735
Moderate to Mild: Subacute Deficit vs. Severe: Lesion Volume	16.7	39.89	-23.19	0.4991	50	50	46.46	735
Moderate to Mild: MRI [Segmented] vs. Moderate to Mild: Lesion Volume	12.3	13.58	-1.284	0.4991	50	50	2.572	735
Moderate to Mild: MRI [Segmented] vs. Moderate: Subacute Deficit	12.3	6.323	5.974	0.4991	50	50	11.97	735
Moderate to Mild: MRI [Segmented] vs. Moderate: MRI [Segmented]	12.3	20.43	-8.137	0.4991	50	50	16.3	735
Moderate to Mild: MRI [Segmented] vs. Moderate: Lesion Volume	12.3	26.9	-14.6	0.4991	50	50	29.25	735
Moderate to Mild: MRI [Segmented] vs. Moderate to Severe: Subacute Deficit	12.3	11.66	0.6347	0.4991	50	50	1.272	735

Continued Table S7

Moderate to Mild: MRI [Segmented] vs. Moderate to Severe: Lesion Volume	12.3	26.52	-14.22	0.4991	50	50	28.5	735
Moderate to Mild: MRI [Segmented] vs. Severe: Subacute Deficit	12.3	24.63	-12.33	0.4991	50	50	24.7	735
Moderate to Mild: MRI [Segmented] vs. Severe: MRI [Segmented]	12.3	37.02	-24.73	0.4991	50	50	49.54	735
Moderate to Mild: MRI [Segmented] vs. Severe: Lesion Volume	12.3	39.89	-27.59	0.4991	50	50	55.28	735
Moderate to Mild: Lesion Volume vs. Moderate: Subacute Deficit	13.58	6.323	7.258	0.4991	50	50	14.54	735
Moderate to Mild: Lesion Volume vs. Moderate: MRI [Segmented]	13.58	20.43	-6.853	0.4991	50	50	13.73	735
Moderate to Mild: Lesion Volume vs. Moderate: Lesion Volume	13.58	26.9	-13.32	0.4991	50	50	26.68	735
Moderate to Mild: Lesion Volume vs. Moderate to Severe: Subacute Deficit	13.58	11.66	1.918	0.4991	50	50	3.843	735
Moderate to Mild: Lesion Volume vs. Moderate to Severe: MRI [Segmented]	13.58	26.02	-12.44	0.4991	50	50	24.93	735
Moderate to Mild: Lesion Volume vs. Moderate to Severe: Lesion Volume	13.58	26.52	-12.94	0.4991	50	50	25.93	735
Moderate to Mild: Lesion Volume vs. Severe: Subacute Deficit	13.58	24.63	-11.05	0.4991	50	50	22.13	735
Moderate to Mild: Lesion Volume vs. Severe: MRI [Segmented]	13.58	37.02	-23.44	0.4991	50	50	46.97	735
Moderate to Mild: Lesion Volume vs. Severe: Lesion Volume	13.58	39.89	-26.31	0.4991	50	50	52.71	735

Continued Table S7

Moderate: Subacute Deficit vs. Moderate: MRI [Segmented]	6.323	20.43	-14.11	0.4991	50	50	28.27	735
Moderate: Subacute Deficit vs. Moderate: Lesion Volume	6.323	26.9	-20.57	0.4991	50	50	41.22	735
Moderate: Subacute Deficit vs. Moderate to Severe: Subacute Deficit	6.323	11.66	-5.339	0.4991	50	50	10.7	735
Moderate: Subacute Deficit vs. Moderate to Severe: MRI [Segmented]	6.323	26.02	-19.7	0.4991	50	50	39.47	735
Moderate: Subacute Deficit vs. Moderate to Severe: Lesion Volume	6.323	26.52	-20.2	0.4991	50	50	40.47	735
Moderate: Subacute Deficit vs. Severe: Subacute Deficit	6.323	24.63	-18.3	0.4991	50	50	36.67	735
Moderate: Subacute Deficit vs. Severe: MRI [Segmented]	6.323	37.02	-30.7	0.4991	50	50	61.51	735
Moderate: Subacute Deficit vs. Severe: Lesion Volume	6.323	39.89	-33.57	0.4991	50	50	67.25	735
Moderate: MRI [Segmented] vs. Moderate: Lesion Volume	20.43	26.9	-6.462	0.4991	50	50	12.95	735
Moderate: MRI [Segmented] vs. Moderate to Severe: Subacute Deficit	20.43	11.66	8.772	0.4991	50	50	17.57	735
Moderate: MRI [Segmented] vs. Moderate to Severe: MRI [Segmented]	20.43	26.02	-5.591	0.4991	50	50	11.2	735
Moderate: MRI [Segmented] vs. Moderate to Severe: Lesion Volume	20.43	26.52	-6.087	0.4991	50	50	12.2	735
Moderate: MRI [Segmented] vs. Severe: Subacute Deficit	20.43	24.63	-4.193	0.4991	50	50	8.401	735
Moderate: MRI [Segmented] vs. Severe: MRI [Segmented]	20.43	37.02	-16.59	0.4991	50	50	33.24	735
Moderate: MRI [Segmented] vs. Severe: Lesion Volume	20.43	39.89	-19.46	0.4991	50	50	38.98	735
Moderate: Lesion Volume vs. Moderate to Severe: Subacute Deficit	26.9	11.66	15.23	0.4991	50	50	30.52	735

Continued Table S7

Moderate: Lesion Volume vs. Moderate to Severe: MRI [Segmented]	26.9	26.02	0.8712	0.4991	50	50	1.745	735
Moderate: Lesion Volume vs. Moderate to Severe: Lesion Volume	26.9	26.52	0.3745	0.4991	50	50	0.7503	735
Moderate: Lesion Volume vs. Severe: Subacute Deficit	26.9	24.63	2.269	0.4991	50	50	4.546	735
Moderate: Lesion Volume vs. Severe: MRI [Segmented]	26.9	37.02	-10.13	0.4991	50	50	20.29	735
Moderate: Lesion Volume vs. Severe: Lesion Volume	26.9	39.89	-12.99	0.4991	50	50	26.03	735
Moderate to Severe: Subacute Deficit vs. Moderate to Severe: MRI [Segmented]	11.66	26.02	-14.36	0.4991	50	50	28.78	735
Moderate to Severe: Subacute Deficit vs. Moderate to Severe: Lesion Volume	11.66	26.52	-14.86	0.4991	50	50	29.77	735
Moderate to Severe: Subacute Deficit vs. Severe: Subacute Deficit	11.66	24.63	-12.96	0.4991	50	50	25.98	735
Moderate to Severe: Subacute Deficit vs. Severe: MRI [Segmented]	11.66	37.02	-25.36	0.4991	50	50	50.81	735
Moderate to Severe: Subacute Deficit vs. Severe: Lesion Volume	11.66	39.89	-28.23	0.4991	50	50	56.56	735
Moderate to Severe: MRI [Segmented] vs. Moderate to Severe: Lesion Volume	26.02	26.52	-0.4967	0.4991	50	50	0.9951	735
Moderate to Severe: MRI [Segmented] vs. Severe: Subacute Deficit	26.02	24.63	1.398	0.4991	50	50	2.8	735
Moderate to Severe: MRI [Segmented] vs. Severe: MRI [Segmented]	26.02	37.02	-11	0.4991	50	50	22.04	735
Moderate to Severe: MRI [Segmented] vs. Severe: Lesion Volume	26.02	39.89	-13.87	0.4991	50	50	27.78	735

Continued Table S7

Moderate to Severe: Lesion Volume vs. Severe: Subacute Deficit	26.52	24.63	1.894	0.4991	50	50	3.795	735
Moderate to Severe: Lesion Volume vs. Severe: MRI [Segmented]	26.52	37.02	-10.5	0.4991	50	50	21.04	735
Moderate to Severe: Lesion Volume vs. Severe: Lesion Volume	26.52	39.89	-13.37	0.4991	50	50	26.78	735
Severe: Subacute Deficit vs. Severe: MRI [Segmented]	24.63	37.02	-12.4	0.4991	50	50	24.84	735
Severe: Subacute Deficit vs. Severe: Lesion Volume	24.63	39.89	-15.26	0.4991	50	50	30.58	735
Severe: MRI [Segmented] vs. Severe: Lesion Volume	37.02	39.89	-2.866	0.4991	50	50	5.742	735

Table S7 Full statistical report on the analysis of prediction accuracy and severity grade for the residual deficit.

Šídák's multiple comparisons test	Mean Diff.	95.00% CI of Diff.	Adjusted P Value
Mild: Lesion Volume vs. Mild: MRI [Segmented]	-20.63	-21.88 to -19.39	<0.0001
Mild: Lesion Volume vs. Moderate to Mild: Lesion Volume	-25.24	-26.49 to -23.99	<0.0001
Mild: Lesion Volume vs. Moderate to Mild: MRI [Segmented]	-13.26	-14.51 to -12.02	<0.0001
Mild: Lesion Volume vs. Moderate: Lesion Volume	-7.857	-9.105 to -6.610	<0.0001
Mild: Lesion Volume vs. Moderate: MRI [Segmented]	-0.3505	-1.598 to 0.8970	>0.9999
Mild: Lesion Volume vs. Moderate to Severe: Lesion Volume	3.616	2.369 to 4.864	<0.0001
Mild: Lesion Volume vs. Moderate to Severe: MRI [Segmented]	4.773	3.526 to 6.021	<0.0001
Mild: Lesion Volume vs. Severe: Lesion Volume	12.66	11.41 to 13.91	<0.0001
Mild: Lesion Volume vs. Severe: MRI [Segmented]	2.137	0.8896 to 3.384	<0.0001
Mild:MRI [Segmented] vs. Moderate to Mild: Lesion Volume	-4.604	-5.851 to -3.356	<0.0001
Mild: MRI [Segmented] vs. Moderate to Mild: MRI [Segmented]	7.371	6.124 to 8.619	<0.0001
Mild: MRI [Segmented] vs. Moderate: Lesion Volume	12.78	11.53 to 14.02	<0.0001
Mild: MRI [Segmented] vs. Moderate: MRI [Segmented]	20.28	19.04 to 21.53	<0.0001
Mild: MRI [Segmented] vs. Moderate to Severe: Lesion Volume	24.25	23.00 to 25.50	<0.0001
Mild: MRI [Segmented] vs. Moderate to Severe: MRI [Segmented]	25.41	24.16 to 26.66	<0.0001
Mild: MRI [Segmented] vs. Severe: Lesion Volume	33.29	32.05 to 34.54	<0.0001
Mild: MRI [Segmented] vs. Severe: MRI [Segmented]	22.77	21.52 to 24.02	<0.0001
Moderate to Mild: Lesion Volume vs. Moderate to Mild: MRI [Segmented]	11.98	10.73 to 13.22	<0.0001
Moderate to Mild: Lesion Volume vs. Moderate: Lesion Volume	17.38	16.13 to 18.63	<0.0001
Moderate to Mild: Lesion Volume vs. Moderate: MRI [Segmented]	24.89	23.64 to 26.14	<0.0001
Moderate to Mild: Lesion Volume vs. Moderate to Severe: Lesion Volume	28.85	27.61 to 30.10	<0.0001
Moderate to Mild: Lesion Volume vs. Moderate to Severe: MRI [Segmented]	30.01	28.76 to 31.26	<0.0001
Moderate to Mild: Lesion Volume vs. Severe: Lesion Volume	37.9	36.65 to 39.15	<0.0001
Moderate to Mild: Lesion Volume vs. Severe: MRI [Segmented]	27.38	26.13 to 28.62	<0.0001
Moderate to Mild: MRI [Segmented] vs. Moderate: Lesion Volume	5.406	4.159 to 6.653	<0.0001
Moderate to Mild: MRI [Segmented] vs. Moderate: MRI [Segmented]	12.91	11.67 to 14.16	<0.0001
Moderate to Mild: MRI [Segmented] vs. Moderate to Severe: Lesion Volume	16.88	15.63 to 18.13	<0.0001
Continued Table S8			

Moderate to Mild: MRI [Segmented] vs. Moderate to Severe: MRI [Segmented]	18.04	16.79 to 19.28	<0.0001
Moderate to Mild: MRI [Segmented] vs. Severe: Lesion Volume	25.92	24.68 to 27.17	<0.0001
Moderate to Mild: MRI [Segmented] vs. Severe: MRI [Segmented]	15.4	14.15 to 16.65	<0.0001
Moderate: Lesion Volume vs. Moderate: MRI [Segmented]	7.507	6.259 to 8.754	<0.0001
Moderate: Lesion Volume vs. Moderate to Severe: Lesion Volume	11.47	10.23 to 12.72	<0.0001
Moderate: Lesion Volume vs. Moderate to Severe: MRI [Segmented]	12.63	11.38 to 13.88	<0.0001
Moderate: Lesion Volume vs. Severe: Lesion Volume	20.52	19.27 to 21.76	<0.0001
Moderate: Lesion Volume vs. Severe: MRI [Segmented]	9.994	8.747 to 11.24	<0.0001
Moderate: MRI [Segmented] vs. Moderate to Severe: Lesion Volume	3.967	2.719 to 5.214	<0.0001
Moderate: MRI [Segmented] vs. Moderate to Severe: MRI [Segmented]	5.124	3.876 to 6.371	<0.0001
Moderate: MRI [Segmented] vs. Severe: Lesion Volume	13.01	11.76 to 14.26	<0.0001
Moderate: MRI [Segmented] vs. Severe: MRI [Segmented]	2.487	1.240 to 3.735	<0.0001
Moderate to Severe: Lesion Volume vs. Moderate to Severe: MRI [Segmented]	1.157	-0.09038 to 2.404	0.1076
Moderate to Severe: Lesion Volume vs. Severe: Lesion Volume	9.043	7.796 to 10.29	<0.0001
Moderate to Severe: Lesion Volume vs. Severe: MRI [Segmented]	-1.479	-2.727 to -0.2317	0.0053
Moderate to Severe: MRI [Segmented] vs. Severe: Lesion Volume	7.886	6.639 to 9.134	<0.0001
Moderate to Severe: MRI [Segmented] vs. Severe: MRI [Segmented]	-2.636	-3.884 to -1.389	<0.0001
Severe: Lesion Volume vs. Severe: MRI [Segmented]	-10.52	-11.77 to -9.275	<0.0001

**Test details**	**Mean 1**	**Mean 2**	**Mean Diff.**	**SE of diff.**	**N1**	**N2**	**t**	**DF**
Mild: Lesion Volume vs. Mild: MRI [Segmented]	18.86	39.5	-20.63	0.3811	50	50	54.14	490
Mild: Lesion Volume vs. Moderate to Mild: Lesion Volume	18.86	44.1	-25.24	0.3811	50	50	66.22	490
Mild: Lesion Volume vs. Moderate to Mild: MRI [Segmented]	18.86	32.13	-13.26	0.3811	50	50	34.8	490
Mild: Lesion Volume vs. Moderate: Lesion Volume	18.86	26.72	-7.857	0.3811	50	50	20.62	490
Mild: Lesion Volume vs. Moderate: MRI [Segmented]	18.86	19.21	-0.3505	0.3811	50	50	0.9195	490
Mild: Lesion Volume vs. Moderate to Severe: Lesion Volume	18.86	15.25	3.616	0.3811	50	50	9.488	490

Continued Table S8

Mild: Lesion Volume vs. Moderate to Severe: MRI [Segmented]	18.86	14.09	4.773	0.3811	50	50	12.52	490
Mild: Lesion Volume vs. Severe: Lesion Volume	18.86	6.205	12.66	0.3811	50	50	33.22	490
Mild: Lesion Volume vs. Severe: MRI [Segmented]	18.86	16.73	2.137	0.3811	50	50	5.607	490
Mild: MRI [Segmented] vs. Moderate to Mild: Lesion Volume	39.5	44.1	-4.604	0.3811	50	50	12.08	490
Mild: MRI [Segmented] vs. Moderate to Mild: MRI [Segmented]	39.5	32.13	7.371	0.3811	50	50	19.34	490
Mild: MRI [Segmented] vs. Moderate: Lesion Volume	39.5	26.72	12.78	0.3811	50	50	33.53	490
Mild: MRI [Segmented] vs. Moderate: MRI [Segmented]	39.5	19.21	20.28	0.3811	50	50	53.22	490
Mild: MRI [Segmented] vs. Moderate to Severe: Lesion Volume	39.5	15.25	24.25	0.3811	50	50	63.63	490
Mild: MRI [Segmented] vs. Moderate to Severe: MRI [Segmented]	39.5	14.09	25.41	0.3811	50	50	66.67	490
Mild: MRI [Segmented] vs. Severe: Lesion Volume	39.5	6.205	33.29	0.3811	50	50	87.36	490
Mild: MRI [Segmented] vs. Severe: MRI [Segmented]	39.5	16.73	22.77	0.3811	50	50	59.75	490
Moderate to Mild: Lesion Volume vs. Moderate to Mild: MRI [Segmented]	44.1	32.13	11.98	0.3811	50	50	31.42	490
Moderate to Mild: Lesion Volume vs. Moderate: Lesion Volume	44.1	26.72	17.38	0.3811	50	50	45.61	490
Moderate to Mild: Lesion Volume vs. Moderate: MRI [Segmented]	44.1	19.21	24.89	0.3811	50	50	65.3	490
Moderate to Mild: Lesion Volume vs. Moderate to Severe: Lesion Volume	44.1	15.25	28.85	0.3811	50	50	75.71	490
Moderate to Mild: Lesion Volume vs. Moderate to Severe: MRI [Segmented]	44.1	14.09	30.01	0.3811	50	50	78.75	490
Moderate to Mild: Lesion Volume vs. Severe: Lesion Volume	44.1	6.205	37.9	0.3811	50	50	99.44	490
Moderate to Mild: Lesion Volume vs. Severe: MRI [Segmented]	44.1	16.73	27.38	0.3811	50	50	71.83	490
Moderate to Mild: MRI [Segmented] vs. Moderate: Lesion Volume	32.13	26.72	5.406	0.3811	50	50	14.18	490
Moderate to Mild: MRI [Segmented] vs. Moderate: MRI [Segmented]	32.13	19.21	12.91	0.3811	50	50	33.88	490

Continued Table S8

Moderate to Mild: MRI [Segmented] vs. Moderate to Severe: Lesion Volume	32.13	15.25	16.88	0.3811	50	50	44.29	490
Moderate to Mild: MRI [Segmented] vs. Moderate to Severe: MRI [Segmented]	32.13	14.09	18.04	0.3811	50	50	47.32	490
Moderate to Mild: MRI [Segmented] vs. Severe: Lesion Volume	32.13	6.205	25.92	0.3811	50	50	68.02	490
Moderate to Mild: MRI [Segmented] vs. Severe: MRI [Segmented]	32.13	16.73	15.4	0.3811	50	50	40.41	490
Moderate: Lesion Volume vs. Moderate: MRI [Segmented]	26.72	19.21	7.507	0.3811	50	50	19.7	490
Moderate: Lesion Volume vs. Moderate to Severe: Lesion Volume	26.72	15.25	11.47	0.3811	50	50	30.1	490
Moderate: Lesion Volume vs. Moderate to Severe: MRI [Segmented]	26.72	14.09	12.63	0.3811	50	50	33.14	490
Moderate: Lesion Volume vs. Severe: Lesion Volume	26.72	6.205	20.52	0.3811	50	50	53.83	490
Moderate: Lesion Volume vs. Severe: MRI [Segmented]	26.72	16.73	9.994	0.3811	50	50	26.22	490
Moderate: MRI [Segmented] vs. Moderate to Severe: Lesion Volume	19.21	15.25	3.967	0.3811	50	50	10.41	490
Moderate: MRI [Segmented] vs. Moderate to Severe: MRI [Segmented]	19.21	14.09	5.124	0.3811	50	50	13.44	490
Moderate: MRI [Segmented] vs. Severe: Lesion Volume	19.21	6.205	13.01	0.3811	50	50	34.14	490
Moderate: MRI [Segmented] vs. Severe: MRI [Segmented]	19.21	16.73	2.487	0.3811	50	50	6.527	490
Moderate to Severe: Lesion Volume vs. Moderate to Severe: MRI [Segmented]	15.25	14.09	1.157	0.3811	50	50	3.036	490
Moderate to Severe: Lesion Volume vs. Severe: Lesion Volume	15.25	6.205	9.043	0.3811	50	50	23.73	490
Moderate to Severe: Lesion Volume vs. Severe: MRI [Segmented]	15.25	16.73	-1.479	0.3811	50	50	3.881	490
Moderate to Severe: MRI [Segmented] vs. Severe: Lesion Volume	14.09	6.205	7.886	0.3811	50	50	20.69	490
Moderate to Severe: MRI [Segmented] vs. Severe: MRI [Segmented]	14.09	16.73	-2.636	0.3811	50	50	6.917	490
Severe: Lesion Volume vs. Severe: MRI [Segmented]	6.205	16.73	-10.52	0.3811	50	50	27.61	490

Table S8 Full statistical report on the analysis of prediction accuracy and severity grade for the subacute deficit in the replication cohort.

Šídák's multiple comparisons test	Mean Diff.	95% CI of Diff.	Adjusted P Value
Mild: MRI [Segmented] vs. Mild: Lesion Volume	-1.146	-3.049 to 0.7569	0.9769
Mild: MRI [Segmented] vs. Mild: Initial Deficit [Day 2-6]	19.25	17.35 to 21.16	<0.0001
Mild: MRI [Segmented] vs. Moderate to Mild: MRI [Segmented]	12.36	10.46 to 14.27	<0.0001
Mild: MRI [Segmented] vs. Moderate to Mild: Lesion Volume	8.36	6.457 to 10.26	<0.0001
Mild: MRI [Segmented] vs. Moderate to Mild: Initial Deficit [Day 2-6]	28.03	26.12 to 29.93	<0.0001
Mild: MRI [Segmented] vs. Moderate: MRI [Segmented]	26.09	24.18 to 27.99	<0.0001
Mild: MRI [Segmented] vs. Moderate: Lesion Volume	28.09	26.19 to 29.99	<0.0001
Mild: MRI [Segmented] vs. Moderate: Initial Deficit [Day 2-6]	27.39	25.48 to 29.29	<0.0001
Mild: MRI [Segmented] vs. Moderate to Severe: MRI [Segmented]	19	17.09 to 20.90	<0.0001
Mild: MRI [Segmented] vs. Moderate to Severe: Lesion Volume	17.94	16.04 to 19.85	<0.0001
Mild: MRI [Segmented] vs. Moderate to Severe: Initial Deficit [Day 2-6]	18.97	17.06 to 20.87	<0.0001
Mild: MRI [Segmented] vs. Severe: MRI [Segmented]	-1.716	-3.618 to 0.1870	0.1593
Mild: MRI [Segmented] vs. Severe: Lesion Volume	15.16	13.25 to 17.06	<0.0001
Mild: MRI [Segmented] vs. Severe: Initial Deficit [Day 2-6]	3.641	1.738 to 5.543	<0.0001
Mild: Lesion Volume vs. Mild: Initial Deficit [Day 2-6]	20.4	18.50 to 22.30	<0.0001
Mild: Lesion Volume vs. Moderate to Mild: MRI [Segmented]	13.51	11.61 to 15.41	<0.0001
Mild: Lesion Volume vs. Moderate to Mild: Lesion Volume	9.506	7.603 to 11.41	<0.0001
Mild: Lesion Volume vs. Moderate to Mild: Initial Deficit [Day 2-6]	29.17	27.27 to 31.07	<0.0001
Mild: Lesion Volume vs. Moderate: MRI [Segmented]	27.23	25.33 to 29.13	<0.0001
Mild: Lesion Volume vs. Moderate: Lesion Volume	29.24	27.33 to 31.14	<0.0001
Mild: Lesion Volume vs. Moderate: Initial Deficit [Day 2-6]	28.53	26.63 to 30.44	<0.0001
Mild: Lesion Volume vs. Moderate to Severe: MRI [Segmented]	20.14	18.24 to 22.05	<0.0001

Continued Table S9

Mild: Lesion Volume vs. Moderate to Severe: Lesion Volume	19.09	17.19 to 20.99	<0.0001
Mild: Lesion Volume vs. Moderate to Severe: Initial Deficit [Day 2-6]	20.11	18.21 to 22.02	<0.0001
Mild: Lesion Volume vs. Severe: MRI [Segmented]	-0.5699	-2.473 to 1.333	>0.9999
Mild: Lesion Volume vs. Severe: Lesion Volume	16.3	14.40 to 18.20	<0.0001
Mild: Lesion Volume vs. Severe: Initial Deficit [Day 2-6]	4.786	2.884 to 6.689	<0.0001
Mild: Initial Deficit [Day 2-6] vs. Moderate to Mild: MRI [Segmented]	-6.889	-8.791 to -4.986	<0.0001
Mild: Initial Deficit [Day 2-6] vs. Moderate to Mild: Lesion Volume	-10.89	-12.80 to -8.990	<0.0001
Mild: Initial Deficit [Day 2-6] vs. Moderate to Mild: Initial Deficit [Day 2-6]	8.773	6.870 to 10.68	<0.0001
Mild: Initial Deficit [Day 2-6] vs. Moderate: MRI [Segmented]	6.833	4.930 to 8.736	<0.0001
Mild: Initial Deficit [Day 2-6] vs. Moderate: Lesion Volume	8.838	6.935 to 10.74	<0.0001
Mild: Initial Deficit [Day 2-6] vs. Moderate: Initial Deficit [Day 2-6]	8.135	6.232 to 10.04	<0.0001
Mild: Initial Deficit [Day 2-6] vs. Moderate to Severe: MRI [Segmented]	-0.2558	-2.159 to 1.647	>0.9999
Mild: Initial Deficit [Day 2-6] vs. Moderate to Severe: Lesion Volume	-1.308	-3.211 to 0.5948	0.8219
Mild: Initial Deficit [Day 2-6] vs. Moderate to Severe: Initial Deficit [Day 2-6]	-0.2855	-2.188 to 1.617	>0.9999
Mild: Initial Deficit [Day 2-6] vs. Severe: MRI [Segmented]	-20.97	-22.87 to -19.07	<0.0001
Mild: Initial Deficit [Day 2-6] vs. Severe: Lesion Volume	-4.098	-6.000 to -2.195	<0.0001
Mild: Initial Deficit [Day 2-6] vs. Severe: Initial Deficit [Day 2-6]	-15.61	-17.51 to -13.71	<0.0001
Moderate to Mild: MRI [Segmented] vs. Moderate to Mild: Lesion Volume	-4.004	-5.907 to -2.101	<0.0001
Moderate to Mild: MRI [Segmented] vs. Moderate to Mild: Initial Deficit [Day 2-6]	15.66	13.76 to 17.56	<0.0001
Moderate to Mild: MRI [Segmented] vs. Moderate: MRI [Segmented]	13.72	11.82 to 15.62	<0.0001
Moderate to Mild: MRI [Segmented] vs. Moderate: Lesion Volume	15.73	13.82 to 17.63	<0.0001

Continued Table S9

Moderate to Mild: MRI [Segmented] vs. Moderate: Initial Deficit [Day 2-6]	15.02	13.12 to 16.93	<0.0001
Moderate to Mild: MRI [Segmented] vs. Moderate to Severe: MRI [Segmented]	6.633	4.730 to 8.536	<0.0001
Moderate to Mild: MRI [Segmented] vs. Moderate to Severe: Lesion Volume	5.581	3.678 to 7.484	<0.0001
Moderate to Mild: MRI [Segmented] vs. Moderate to Severe: Initial Deficit [Day 2-6]	6.603	4.701 to 8.506	<0.0001
Moderate to Mild: MRI [Segmented] vs. Severe: MRI [Segmented]	-14.08	-15.98 to -12.18	<0.0001
Moderate to Mild: MRI [Segmented] vs. Severe: Lesion Volume	2.791	0.8884 to 4.694	<0.0001
Moderate to Mild: MRI [Segmented] vs. Severe: Initial Deficit [Day 2-6]	-8.723	-10.63 to -6.821	<0.0001
Moderate to Mild: Lesion Volume vs. Moderate to Mild: Initial Deficit [Day 2-6]	19.67	17.76 to 21.57	<0.0001
Moderate to Mild: Lesion Volume vs. Moderate: MRI [Segmented]	17.73	15.82 to 19.63	<0.0001
Moderate to Mild: Lesion Volume vs. Moderate: Lesion Volume	19.73	17.83 to 21.63	<0.0001
Moderate to Mild: Lesion Volume vs. Moderate: Initial Deficit [Day 2-6]	19.03	17.12 to 20.93	<0.0001
Moderate to Mild: Lesion Volume vs. Moderate to Severe: MRI [Segmented]	10.64	8.734 to 12.54	<0.0001
Moderate to Mild: Lesion Volume vs. Moderate to Severe: Lesion Volume	9.585	7.682 to 11.49	<0.0001
Moderate to Mild: Lesion Volume vs. Moderate to Severe: Initial Deficit [Day 2-6]	10.61	8.704 to 12.51	<0.0001
Moderate to Mild: Lesion Volume vs. Severe: MRI [Segmented]	-10.08	-11.98 to -8.173	<0.0001
Moderate to Mild: Lesion Volume vs. Severe: Lesion Volume	6.795	4.892 to 8.698	<0.0001
Moderate to Mild: Lesion Volume vs. Severe: Initial Deficit [Day 2-6]	-4.72	-6.622 to -2.817	<0.0001
Moderate to Mild: Initial Deficit [Day 2-6] vs. Moderate: MRI [Segmented]	-1.94	-3.842 to -0.03692	0.039
Moderate to Mild: Initial Deficit [Day 2-6] vs. Moderate: Lesion Volume	0.06519	-1.838 to 1.968	>0.9999
Continued Table S9			

Moderate to Mild: Initial Deficit [Day 2-6] vs. Moderate: Initial Deficit [Day 2-6]	-0.6379	-2.541 to 1.265	>0.9999
Moderate to Mild: Initial Deficit [Day 2-6] vs. Moderate to Severe: MRI [Segmented]	-9.028	-10.93 to -7.126	<0.0001
Moderate to Mild: Initial Deficit [Day 2-6] vs. Moderate to Severe: Lesion Volume	-10.08	-11.98 to -8.178	<0.0001
Moderate to Mild: Initial Deficit [Day 2-6] vs. Moderate to Severe: Initial Deficit [Day 2-6]	-9.058	-10.96 to -7.156	<0.0001
Moderate to Mild: Initial Deficit [Day 2-6] vs. Severe: MRI [Segmented]	-29.74	-31.64 to -27.84	<0.0001
Moderate to Mild: Initial Deficit [Day 2-6] vs. Severe: Lesion Volume	-12.87	-14.77 to -10.97	<0.0001
Moderate to Mild: Initial Deficit [Day 2-6] vs. Severe: Initial Deficit [Day 2-6]	-24.38	-26.29 to -22.48	<0.0001
Moderate: MRI [Segmented] vs. Moderate: Lesion Volume	2.005	0.1021 to 3.908	0.0249
Moderate: MRI [Segmented] vs. Moderate: Initial Deficit [Day 2-6]	1.302	-0.6009 to 3.204	0.8313
Moderate: MRI [Segmented] vs. Moderate to Severe: MRI [Segmented]	-7.089	-8.992 to -5.186	<0.0001
Moderate: MRI [Segmented] vs. Moderate to Severe: Lesion Volume	-8.141	-10.04 to -6.238	<0.0001
Moderate: MRI [Segmented] vs. Moderate to Severe: Initial Deficit [Day 2-6]	-7.119	-9.021 to -5.216	<0.0001
Moderate: MRI [Segmented] vs. Severe: MRI [Segmented]	-27.8	-29.70 to -25.90	<0.0001
Moderate: MRI [Segmented] vs. Severe: Lesion Volume	-10.93	-12.83 to -9.028	<0.0001
Moderate: MRI [Segmented] vs. Severe: Initial Deficit [Day 2-6]	-22.45	-24.35 to -20.54	<0.0001
Moderate: Lesion Volume vs. Moderate: Initial Deficit [Day 2-6]	-0.7031	-2.606 to 1.200	>0.9999
Moderate: Lesion Volume vs. Moderate to Severe: MRI [Segmented]	-9.094	-11.00 to -7.191	<0.0001
Moderate: Lesion Volume vs. Moderate to Severe: Lesion Volume	-10.15	-12.05 to -8.243	<0.0001
Moderate: Lesion Volume vs. Moderate to Severe: Initial Deficit [Day 2-6]	-9.123	-11.03 to -7.221	<0.0001

Continued Table S9

Moderate: Lesion Volume vs. Severe: MRI [Segmented]	-29.81	-31.71 to -27.90	<0.0001
Moderate: Lesion Volume vs. Severe: Lesion Volume	-12.94	-14.84 to -11.03	<0.0001
Moderate: Lesion Volume vs. Severe: Initial Deficit [Day 2-6]	-24.45	-26.35 to -22.55	<0.0001
Moderate: Initial Deficit [Day 2-6] vs. Moderate to Severe: MRI [Segmented]	-8.391	-10.29 to -6.488	<0.0001
Moderate: Initial Deficit [Day 2-6] vs. Moderate to Severe: Lesion Volume	-9.443	-11.35 to -7.540	<0.0001
Moderate: Initial Deficit [Day 2-6] vs. Moderate to Severe: Initial Deficit [Day 2-6]	-8.42	-10.32 to -6.518	<0.0001
Moderate: Initial Deficit [Day 2-6] vs. Severe: MRI [Segmented]	-29.1	-31.01 to -27.20	<0.0001
Moderate: Initial Deficit [Day 2-6] vs. Severe: Lesion Volume	-12.23	-14.14 to -10.33	<0.0001
Moderate: Initial Deficit [Day 2-6] vs. Severe: Initial Deficit [Day 2-6]	-23.75	-25.65 to -21.84	<0.0001
Moderate to Severe: MRI [Segmented] vs. Moderate to Severe: Lesion Volume	-1.052	-2.955 to 0.8506	0.9968
Moderate to Severe: MRI [Segmented] vs. Moderate to Severe: Initial Deficit [Day 2-6]	-0.02972	-1.932 to 1.873	>0.9999
Moderate to Severe: MRI [Segmented] vs. Severe: MRI [Segmented]	-20.71	-22.62 to -18.81	<0.0001
Moderate to Severe: MRI [Segmented] vs. Severe: Lesion Volume	-3.842	-5.745 to -1.939	<0.0001
Moderate to Severe: MRI [Segmented] vs. Severe: Initial Deficit [Day 2-6]	-15.36	-17.26 to -13.45	<0.0001
Moderate to Severe: Lesion Volume vs. Moderate to Severe: Initial Deficit [Day 2-6]	1.022	-0.8803 to 2.925	0.9985
Moderate to Severe: Lesion Volume vs. Severe: MRI [Segmented]	-19.66	-21.56 to -17.76	<0.0001
Moderate to Severe: Lesion Volume vs. Severe: Lesion Volume	-2.79	-4.692 to -0.8870	<0.0001
Moderate to Severe: Lesion Volume vs. Severe: Initial Deficit [Day 2-6]	-14.3	-16.21 to -12.40	<0.0001
Moderate to Severe: Initial Deficit [Day 2-6] vs. Severe: MRI [Segmented]	-20.68	-22.59 to -18.78	<0.0001

Continued Table S9

Moderate to Severe: Initial Deficit [Day 2-6] vs. Severe: Lesion Volume	-3.812	-5.715 to -1.909	<0.0001
Moderate to Severe: Initial Deficit [Day 2-6] vs. Severe: Initial Deficit [Day 2-6]	-15.33	-17.23 to -13.42	<0.0001
Severe: MRI [Segmented] vs. Severe: Lesion Volume	16.87	14.97 to 18.77	<0.0001
Severe: MRI [Segmented] vs. Severe: Initial Deficit [Day 2-6]	5.356	3.454 to 7.259	<0.0001
Severe: Lesion Volume vs. Severe: Initial Deficit [Day 2-6]	-11.51	-13.42 to -9.612	<0.0001

**Test details**	**Mean 1**	**Mean 2**	**Mean Diff.**	**SE of Diff.**	**N1**	**N2**	**t**	**DF**
Mild: Subacute Deficit vs. Mild: MRI [Segmented]	15.38	34.64	-19.25	0.5432	50	50	35.44	735
Mild: Subacute Deficit vs. Mild: Lesion Volume	15.38	35.78	-20.4	0.5432	50	50	37.55	735
Mild: Subacute Deficit vs. Moderate to Mild: Subacute Deficit	15.38	6.611	8.773	0.5432	50	50	16.15	735
Mild: Subacute Deficit vs. Moderate to Mild: MRI [Segmented]	15.38	22.27	-6.889	0.5432	50	50	12.68	735
Mild: Subacute Deficit vs. Moderate to Mild: Lesion Volume	15.38	26.28	-10.89	0.5432	50	50	20.05	735
Mild: Subacute Deficit vs. Moderate: Subacute Deficit	15.38	7.248	8.135	0.5432	50	50	14.98	735
Mild: Subacute Deficit vs. Moderate: MRI [Segmented]	15.38	8.55	6.833	0.5432	50	50	12.58	735
Mild: Subacute Deficit vs. Moderate: Lesion Volume	15.38	6.545	8.838	0.5432	50	50	16.27	735
Mild: Subacute Deficit vs. Moderate to Severe: Subacute Deficit	15.38	15.67	-0.2855	0.5432	50	50	0.5256	735
Mild: Subacute Deficit vs. Moderate to Severe: MRI [Segmented]	15.38	15.64	-0.2558	0.5432	50	50	0.4709	735
Mild: Subacute Deficit vs. Moderate to Severe: Lesion Volume	15.38	16.69	-1.308	0.5432	50	50	2.408	735
Mild: Subacute Deficit vs. Severe: Subacute Deficit	15.38	31	-15.61	0.5432	50	50	28.74	735
Mild: Subacute Deficit vs. Severe: MRI [Segmented]	15.38	36.35	-20.97	0.5432	50	50	38.6	735
Mild: Subacute Deficit vs. Severe: Lesion Volume	15.38	19.48	-4.098	0.5432	50	50	7.543	735

Continued Table S9

Mild: MRI [Segmented] vs. Mild: Lesion Volume	34.64	35.78	-1.146	0.5432	50	50	2.109	735
Mild: MRI [Segmented] vs. Moderate to Mild: Subacute Deficit	34.64	6.611	28.03	0.5432	50	50	51.59	735
Mild: MRI [Segmented] vs. Moderate to Mild: MRI [Segmented]	34.64	22.27	12.36	0.5432	50	50	22.76	735
Mild: MRI [Segmented] vs. Moderate to Mild: Lesion Volume	34.64	26.28	8.36	0.5432	50	50	15.39	735
Mild: MRI [Segmented] vs. Moderate: Subacute Deficit	34.64	7.248	27.39	0.5432	50	50	50.42	735
Mild: MRI [Segmented] vs. Moderate: MRI [Segmented]	34.64	8.55	26.09	0.5432	50	50	48.02	735
Mild: MRI [Segmented] vs. Moderate: Lesion Volume	34.64	6.545	28.09	0.5432	50	50	51.71	735
Mild: MRI [Segmented] vs. Moderate to Severe: Subacute Deficit	34.64	15.67	18.97	0.5432	50	50	34.92	735
Mild: MRI [Segmented] vs. Moderate to Severe: MRI [Segmented]	34.64	15.64	19	0.5432	50	50	34.97	735
Mild: MRI [Segmented] vs. Moderate to Severe: Lesion Volume	34.64	16.69	17.94	0.5432	50	50	33.03	735
Mild: MRI [Segmented] vs. Severe: Subacute Deficit	34.64	31	3.641	0.5432	50	50	6.702	735
Mild: MRI [Segmented] vs. Severe: MRI [Segmented]	34.64	36.35	-1.716	0.5432	50	50	3.158	735
Mild: MRI [Segmented] vs. Severe: Lesion Volume	34.64	19.48	15.16	0.5432	50	50	27.9	735
Mild: Lesion Volume vs. Moderate to Mild: Subacute Deficit	35.78	6.611	29.17	0.5432	50	50	53.7	735
Mild: Lesion Volume vs. Moderate to Mild: MRI [Segmented]	35.78	22.27	13.51	0.5432	50	50	24.87	735
Mild: Lesion Volume vs. Moderate to Mild: Lesion Volume	35.78	26.28	9.506	0.5432	50	50	17.5	735
Mild: Lesion Volume vs. Moderate: Subacute Deficit	35.78	7.248	28.53	0.5432	50	50	52.53	735
Mild: Lesion Volume vs. Moderate: MRI [Segmented]	35.78	8.55	27.23	0.5432	50	50	50.13	735
Mild: Lesion Volume vs. Moderate: Lesion Volume	35.78	6.545	29.24	0.5432	50	50	53.82	735

Continued Table S9

Mild: Lesion Volume vs. Moderate to Severe: Subacute Deficit	35.78	15.67	20.11	0.5432	50	50	37.03	735
Mild: Lesion Volume vs. Moderate to Severe: MRI [Segmented]	35.78	15.64	20.14	0.5432	50	50	37.08	735
Mild: Lesion Volume vs. Moderate to Severe: Lesion Volume	35.78	16.69	19.09	0.5432	50	50	35.14	735
Mild: Lesion Volume vs. Severe: Subacute Deficit	35.78	31	4.786	0.5432	50	50	8.811	735
Mild: Lesion Volume vs. Severe: MRI [Segmented]	35.78	36.35	-0.5699	0.5432	50	50	1.049	735
Mild: Lesion Volume vs. Severe: Lesion Volume	35.78	19.48	16.3	0.5432	50	50	30.01	735
Moderate to Mild: Subacute Deficit vs. Moderate to Mild: MRI [Segmented]	6.611	22.27	-15.66	0.5432	50	50	28.83	735
Moderate to Mild: Subacute Deficit vs. Moderate to Mild: Lesion Volume	6.611	26.28	-19.67	0.5432	50	50	36.2	735
Moderate to Mild: Subacute Deficit vs. Moderate: Subacute Deficit	6.611	7.248	-0.6379	0.5432	50	50	1.174	735
Moderate to Mild: Subacute Deficit vs. Moderate: MRI [Segmented]	6.611	8.55	-1.94	0.5432	50	50	3.571	735
Moderate to Mild: Subacute Deficit vs. Moderate: Lesion Volume	6.611	6.545	0.06519	0.5432	50	50	0.12	735
Moderate to Mild: Subacute Deficit vs. Moderate to Severe: Subacute Deficit	6.611	15.67	-9.058	0.5432	50	50	16.68	735

Table S9 Full statistical report on the analysis of prediction accuracy and severity grade for the residual deficit in the replication cohort.
